# Specific Recognition of β-Galactofuranose-Containing Glycans of Synthetic Neoglycoproteins by Sera of Chronic Chagas Disease Patients

**DOI:** 10.3390/molecules27020411

**Published:** 2022-01-09

**Authors:** Alba L. Montoya, Eileni R. Gil, Emily L. Heydemann, Igor L. Estevao, Bianca E. Luna, Cameron C. Ellis, Sohan R. Jankuru, Belkisyolé Alarcón de Noya, Oscar Noya, Maria Paola Zago, Igor C. Almeida, Katja Michael

**Affiliations:** 1 Department of Chemistry and Biochemistry, Border Biomedical Research Center, University of Texas at El Paso, El Paso, TX 79968, USA; Alba.Montoya@pharm.utah.edu (A.L.M.); erodriguez102@miners.utep.edu (E.R.G.); heydemann@livemail.uthscsa.edu (E.L.H.); srdesilva@utep.edu (S.R.J.); 2 Department of Biological Sciences, Border Biomedical Research Center, University of Texas at El Paso, El Paso, TX 79968, USA; ilestevaoda@utep.edu (I.L.E.); beluna2@miners.utep.edu (B.E.L.); ccellis@miners.utep.edu (C.C.E.); 3 Sección de Inmunología, Instituto de Medicina Tropical, Facultad de Medicina, Universidad Central de Venezuela, Caracas 1041-A, Venezuela; belkisyole@gmail.com (B.A.d.N.); noyaoo@yahoo.com (O.N.); 4 Centro para Estudios Sobre Malaria, Instituto de Altos Estudios “Dr. Arnoldo Gabaldón”, Instituto Nacional de Higiene Rafael Rangel, Ministerio del Poder Popular para la Salud, Caracas 1041-A, Venezuela; 5 Instituto de Patología Experimental, Facultad de Ciencias de la Salud, Universidad Nacional de Salta (UNSa)-Consejo Nacional de Investigaciones Científicas y Técnicas (CONICET), Salta 4400, Argentina; mpzago@conicet.gov.ar

**Keywords:** biomarker, Chagas disease, chemiluminescent enzyme-linked immunosorbent assay, galactofuranose, neoglycoproteins, oligosaccharide synthesis

## Abstract

Chagas disease (CD) can be accurately diagnosed by detecting *Trypanosoma cruzi* in patients’ blood using polymerase chain reaction (PCR). However, parasite-derived biomarkers are of great interest for the serological diagnosis and early evaluation of chemotherapeutic efficacy when PCR may fail, owing to a blood parasite load below the method’s limit of detection. Previously, we focused on the detection of specific anti-α-galactopyranosyl (α-Gal) antibodies in chronic CD (CCD) patients elicited by α-Gal glycotopes copiously expressed on insect-derived and mammal-dwelling infective parasite stages. Nevertheless, these stages also abundantly express cell surface glycosylphosphatidylinositol (GPI)-anchored glycoproteins and glycoinositolphospholipids (GIPLs) bearing nonreducing terminal β-galactofuranosyl (β-Gal*f*) residues, which are equally foreign to humans and, therefore, highly immunogenic. Here we report that CCD patients’ sera react specifically with synthetic β-Gal*f*-containing glycans. We took a reversed immunoglycomics approach that entailed: (a) Synthesis of *T. cruzi* GIPL-derived Gal*f*β1,3Man*p*α-(CH_2_)_3_SH (glycan **G29_SH_**) and Gal*f*β1,3Man*p*α1,2-[Gal*f*β1,3]Man*p*α-(CH_2_)_3_SH (glycan **G32_SH_**); and (b) preparation of neoglycoproteins **NGP29b** and **NGP32b**, and their evaluation in a chemiluminescent immunoassay. Receiver-operating characteristic analysis revealed that **NGP32b** can distinguish CCD sera from sera of healthy individuals with 85.3% sensitivity and 100% specificity. This suggests that Gal*f*β1,3Man*p*α1,2-[Gal*f*β1,3]Man*p*α is an immunodominant glycotope and that **NGP32b** could potentially be used as a novel CCD biomarker.

## 1. Introduction

The protozoan parasite *Trypanosoma cruzi* is the causative agent of Chagas disease (CD), which is endemic throughout Latin America, where millions of people are affected. The disease is associated with significant morbidity in 30–40% of patients, who may suffer from severe and life-threatening conditions, including cardiomyopathy and megasyndromes (megacolon and megaesophagus) [1]. There are approximately 12,000 CD-related deaths annually [2]. Two chemotherapy drugs are available (i.e., benznidazole and nifurtimox), which are >90% effective in the acute stage and 60–80% effective in the chronic stage of the disease [3]. CD can be accurately diagnosed by detecting parasite DNA in patient’s blood using polymerase chain reaction (PCR) [4]. However, a negative PCR test does not necessarily mean cure, as the concentration of circulating parasites may have decreased below the limit of detection (LOD) of the assay, and/or parasites may also still reside in the tissues [5]. Detection of anti-parasitic antibodies (Abs) in chronic CD (CCD) may be more suitable for evaluating treatment efficacy because the continuous stimulation of the immune system even by very few bloodstream or tissue-residing parasites, or circulating *T. cruzi*-derived antigens results in high levels of Ab titers [6].

While serological immunoassays using parasite lysates as antigenic preparation have been well-established, these lysates are highly heterogeneous mixtures to which Ab responses are complex, including specific and nonspecific Ab binding to different antigens, as well as undesired cross-reactivities with Abs elicited to other pathogens such as *Leishmania* spp. [7,8,9]. Therefore, specific parasite-derived serological biomarkers (BMKs) are of great interest, not only for the development of diagnostic assays, such as enzyme-linked immunosorbent assay (ELISA) and lateral flow assay (LFA), but particularly for the early evaluation of chemotherapy efficacy of CD [6]. The mammal-dwelling *T. cruzi* trypomastigote (TCT) life-cycle stage or form is known to copiously express glycosylphosphatidylinositol (GPI)-anchored mucins (tGPI-mucins), which are heavily *O*-glycosylated. Many of the glycans have terminal nonreducing α-galactopyranosyl (α-Gal*p*) residues, which elicit *T. cruzi*-specific, lytic anti-α-Gal Abs (or Ch anti-α-Gal Abs), present at high levels in all chronic CCD patients [7,10,11,12,13]. It has been shown that the Ch anti-α-Gal Abs in children with early CCD undergo seroconversion in 58% of patients following three years of benznidazole (BZN) treatment. In adults, the kinetics of Ch anti-α-Gal Ab seroconversion seems to be slower, as observed after one-year follow-up in two recent clinical trials [14,15]. However, a long-term (three-year) follow-up of treated patients is currently being performed in an adult population using tGPI-mucins and synthetic α-Gal-based antigens to evaluate the seroconversion kinetics in adults with CCD [16]. Taken together, these studies suggest that the seroconversion of Abs against parasite-derived carbohydrate antigens could by useful for determining cure following chemotherapy.

**Figure 1:**
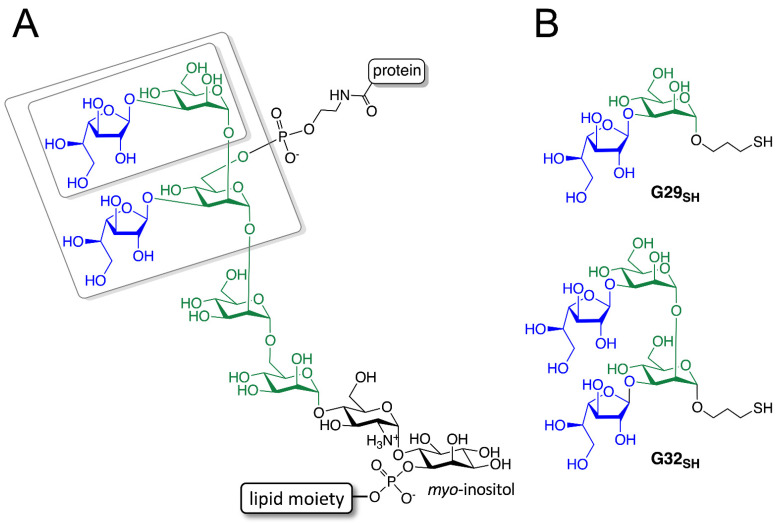
(**A**) Proposed β-galactofuranosylated GPI anchor of the tGPI-mucins. Potential β-Gal*f*- and α-Man-containing glycotopes are highlighted in the boxes. (**B**) The glycans **G29_SH_** and **G32_SH_** synthesized and further explored in this study are derived from the proposed tGPI-mucin GPI structure shown.

One aspect that somewhat complicates the serological detection of Ch anti-α-Gal Abs is that normal human serum (NHS) also contains anti-α-Gal Abs (NHS anti-α-Gal Abs). However, the two types of anti-α-Gal Abs differ in their concentration and binding strength to α-Gal-containing *T. cruzi* antigens. While the NHS anti-α-Gal Abs are highly abundant and constitute 1% of circulating IgG [17], in patients with CCD, Ch anti-α-Gal Abs are even more abundant and react specifically with *T. cruzi*-derived α-Gal-containing antigens with greater binding strength [12,13]. However, NHS anti-α-Gal Abs are responsible for a small amount of cross-reactivity observable by CL-ELISA. To avoid the cross-reactivity with NHS anti-α-Gal Abs, there is an interest in identifying other *T. cruzi* serological BMKs that have a different specificity profile. Potential candidates are *T. cruzi*-derived β-galactofuranose (β-Gal*f*)-containing antigens. β-Gal*f* is completely absent in humans, and such glycans elicit a strong immune response in CD patients [18,19,20]. Furthermore, unlike anti-α-Gal Abs, NHS does not normally contain significant amounts of anti-β-Gal*f* Abs unless an individual had been recently infected with a microorganism that expresses β-Gal*f* (e.g., certain *Leishmania* species [21], certain pathogenic bacteria [22,23], or fungi [24]). Terminal nonreducing β-Gal*f* residues are components of the major GIPL (formerly, lipopeptidoglycan, LPPG) species from *T. cruzi* of all strains thus far studied [25,26,27]. Analysis of the primary structure of the GIPLs of the epimastigote form of *T. cruzi* revealed that the conserved oligosaccharide core, Manα1,2Manα1,6Manα1,4GlcN, may be extended with another mannose residue, and with one or two galactofuranose residues, resulting in microheterogeneity [28,29]. The most abundant GIPL found had the structure Gal*f*β1,3Manα1,2[Gal*f*β1,3]Manα1,2Manα1,6Manα1,4[AEP6]GlcNα1,6PI (AEP = aminoethylphosphonate; PI = phosphatidylinositol) [29]. Furthermore, some *T. cruzi* strains, i.e., Colombiana, Dm28c, and Tulahuén, express mucin *O*-glycans with terminal nonreducing β-Gal*f* residues [30]. Studies on the GPI anchors of the TCT-derived mucins (tGPI-mucins) of the Y-strain have shown that they consist of four to eight hexoses with a mannose/galactose ratio of 4:2.5 [31], also pointing to microheterogeneity. Treatment of the GPI moiety of tGPI-mucins with hydrofluoric acid releases up to four galactose units per GPI, which suggests that one or more of these galactose moieties may exist in their furanoid forms [Almeida, I.C. unpublished data]. These data suggest that the galactose/mannose ratio in the GPIs increases on average when epimastigotes develop into trypomastigotes. Previous studies have shown that the sera of CCD patients contain not only anti-α-Gal Abs, but also anti-β-Gal*f* Abs [18,20,32]. The major molecular targets of anti-β-Gal*f* Abs in CD are most likely protein-free GIPLs and β-Gal*f*-containing *O*-glycans on GPI-anchored glycoproteins (e.g., mucins) of the infective insect-vector-derived metacyclic trypomastigote (Meta) and mammal-dwelling TCT forms [20,26,27,33,34,35]. Moreover, given that all *T. cruzi* glycoprotein GPI moieties and GIPLs described so far are based on the (Manα1,2)Manα1,2Manα1,6Manα1,4GlcNα1,6PI structure [25,26,27,29,34,36,37,38,39], one of several possible TCT-derived GPI-anchor structures is proposed in Figure 1. 

Here we show the synthesis of two β-Gal*f*-containing glycans, which could potentially be terminal partial structures of a TCT GPI anchor (Figure 1A). The glycans were conjugated to bovine serum albumin (BSA), and the resulting NGPs were immunologically evaluated using sera of CCD patients as well as sera of healthy individuals as a negative control group.

## 2. Results and Discussion

**Scheme 1:**
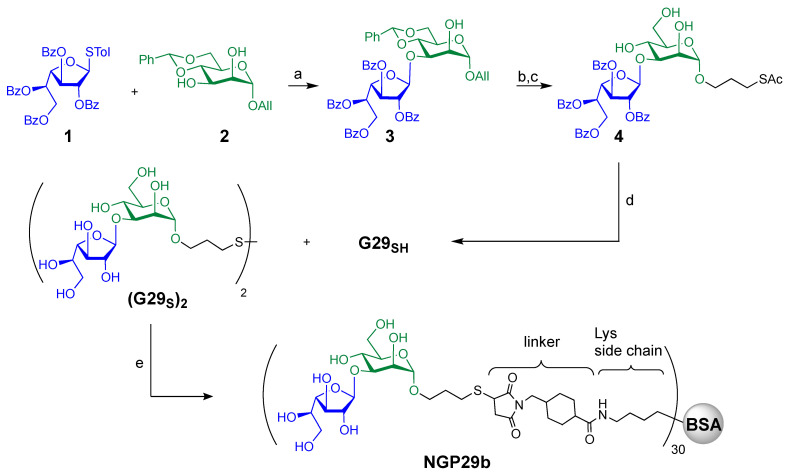
Synthesis of disaccharide **G29_SH_** and its conjugation to BSA affording **NGP29b**: (a) NIS, AgOTf, DCM 0 °C to rt, 70%; (b) TFA, H_2_O, rt, 53%; (c) AcSH, AIBN, THF, UV light (λ = 350 nm), 79%; (d) NaOMe, MeOH, rt, quant; and (e) TCEP, maleimide-activated BSA, phosphate buffer pH 7.2.

We hypothesized that a β-Gal*f*-based glycotope could be identified and exploited as a new specific BMK for CD which, unlike previously discovered glycan-based BMKs for CD, is free of α-Gal, and therefore shows less cross-reactivity with NHS. To address this hypothesis, we took a reversed immunoglycomics approach [40] that began with the synthesis of Gal*f*β1,3Man*p*α-CH_2_CH_2_CH_2_SH (**G29_SH_**) and Gal*f*β1,3Man*p*α1,2-[Gal*f*β1,3]Man*p*α-CH_2_CH_2_CH_2_SH (**G32_SH_**) (Figure 1B). These target compounds contain terminal disaccharide and tetrasaccharide partial structures of a TCT GPI-anchor (Figure 1A) [31,33], respectively, and a 3-thiopropyl moiety allowing for conjugation to maleimide-derivatized BSA. Derivatives of Gal*f*β1,3Man*p*α [41,42] and Gal*f*β1,3Man*p*α1,2-[Gal*f*β1,3]Man*p*α [41], as well as the terminal trisaccharide of a *Leishmania* type-2 GIPL [40,42], and a *Leishmania* lipophosphoglycan (LPG) core heptasaccharyl *myo*-inositol [43], and a *T. cruzi* LPPG heptasaccharyl *myo*-inositol [44] have previously been synthesized. Our synthesis of disaccharide **G29_SH_** and its conjugation to commercially available maleimide-activated BSA is illustrated in Scheme 1. The benzoylated thiogalactofuranoside donor **1** [45] was used to glycosylate allyl mannoside acceptor **2** [46] to produce disaccharide **3**. The regioselectivity using this or a similar mannosyl acceptor had previously been shown [40,42]. Hydrolysis of the benzylidene protecting group and radical addition of thioacetic acid to the terminal alkene afforded thioester **4**. Global removal of all acyl groups under Zemplén conditions gave the target disaccharide **G29_SH_**, which oxidized to **(G29_S_)_2_** on air. Upon reduction of the disulfide with TCEP, **G29_SH_** was conjugated to commercial maleimide-derivatized BSA to give rise to the neoglycoprotein (NGP) **NGP29b** with a high payload of 30 units of disaccharide per BSA molecule, as observed by matrix-assisted laser desorption/ionization time-of-flight mass spectrometry (MALDI-TOF-MS) (see Supplementary Material).

**Scheme 2:**
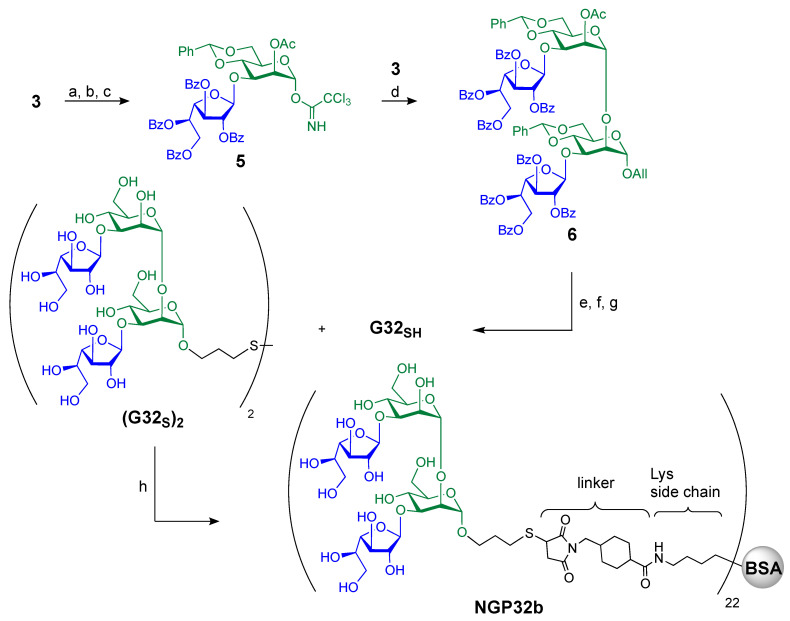
Synthesis of tetrasaccharide **G32_SH_** and its conjugation to BSA affording **NGP32b**: (a) Ac_2_O, DMAP, pyr, 0 °C to rt, 82%; (b) PdCl_2_, MeOH, rt, 80%; (c) Cl_3_CCN, DBU, DCM, 0 °C to rt, 87%; (d) BF_3_-Et_2_O, DCM, 0 °C to rt, 75%; (e) TFA/H_2_O/DCM 1:1:10, rt, 93%; (f) AcSH, DPAP, DCM, UV light (λ = 350 nm), 92%; (g) NaOMe, MeOH, rt, 75%.; and (h) TCEP, maleimide-activated BSA, phosphate buffer pH 7.2.

Scheme 2 shows the synthesis of tetrasaccharide **G32_SH_** and its conjugation to maleimide-derivatized BSA to produce **NGP32b**. First, previously synthesized disaccharide **3** was acetylated, then the anomeric allyl group was removed under PdCl_2_ catalysis, and the resulting hemiacetal was converted to trichloroacetimidate **5**. This donor was used to glycosylate disaccharide acceptor **3** to furnish the fully protected tetrasaccharide **6** in good yield. Hydrolysis of the benzylidene acetal followed by radical addition of thioacetic acid to the terminal alkene gave the thioester **S5** (see Supplementary Material). Global deprotection of all ester groups under Zemplén conditions afforded **G32_SH_**, which oxidized to **(G32_S_)_2_** on air. The disulfide was reduced with TCEP followed by conjugation to commercial maleimide-derivatized BSA to furnish **NGP32b**, as observed by MALDI-TOF-MS (see Supplementary Material).

**Figure 2:**
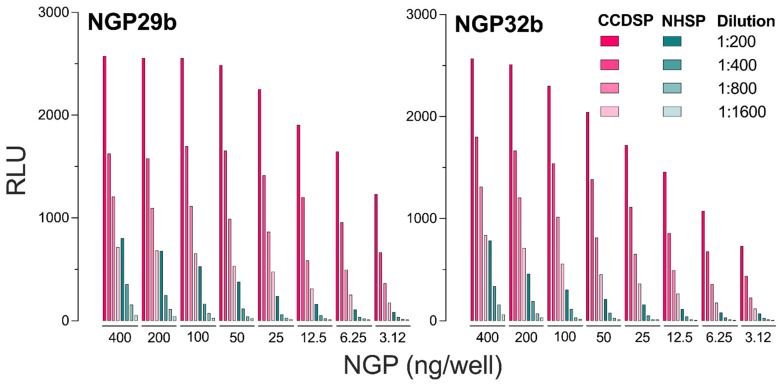
Antigen and serum cross-titration by CL-ELISA of **NGP29b** and **NGP32b**. This experiment aimed to identify CL-ELISA conditions that give the largest differential reactivity between CCD sera (red) and NHS control group (green). Both NGPs were titrated at different concentrations (3.12, 6.25, 12.5, 25, 50, 100, 200, or 400 ng/well) with serum pools from ten CCD patients and from ten healthy individuals, both diluted at 1:200, 1:400, 1:800, or 1:1600. The CL-ELISA was performed as described in the Supplementary Material. CCDSP, CCD serum pool; NHSP, normal human serum pool; RLU, relative luminescence units.

With the **NGP29b** and **NGP32b** in hand, IgG Ab responses using sera from CCD patients could now be measured by CL-ELISA, as previously described [40,47,48,49]. First, to identify the most suitable CL-ELISA conditions, sera from 10 patients with CCD from an endemic region (Venezuela) and 10 sera from healthy individuals from a nonendemic region (USA) were pooled. Figure 2 shows the CL-ELISA cross-titrations of varying pooled serum dilutions and NGP antigen loadings [ng/well]. Analysis of the data indicated that a serum dilution of 1:800 and 50 ng of NGP antigen per well gave the best differential IgG responses when comparing CCDSP vs. NHSP. Under these conditions, the difference of IgG reactivity between the CCDSP and the NHSP was a factor of 24 for **NGP29b**, and a factor of 32 for **NGP32b**. These differential reactivities are by a factor of roughly 2–3 greater when compared to Gal*p*α1,3Gal*p*β-containing NGPs observed in similar CL-ELISA studies [47,49]. Only one NGP that contained Gal*p*α1,3Gal*p*β1,4GlcNAcα (“**NGP24b**”) was able to reach an antibody differential reactivity of a factor of ~20 [48]. Our previous studies with CCD and cutaneous leishmaniasis patient sera had shown that the binding of serum components to BSA or the same crosslinker present in **NGP29b** and **NGP32b** was negligible [40,49]. The cross-titrations shown in Figure 2 already indicated that both, **NGP29b** and **NGP32b**, could potentially be BMKs for CD. However, to obtain sensitivity and specificity data, which determine their suitability as a BMK, CL-ELISAs of individual sera (not pooled sera) are necessary. 

**Figure 3:**
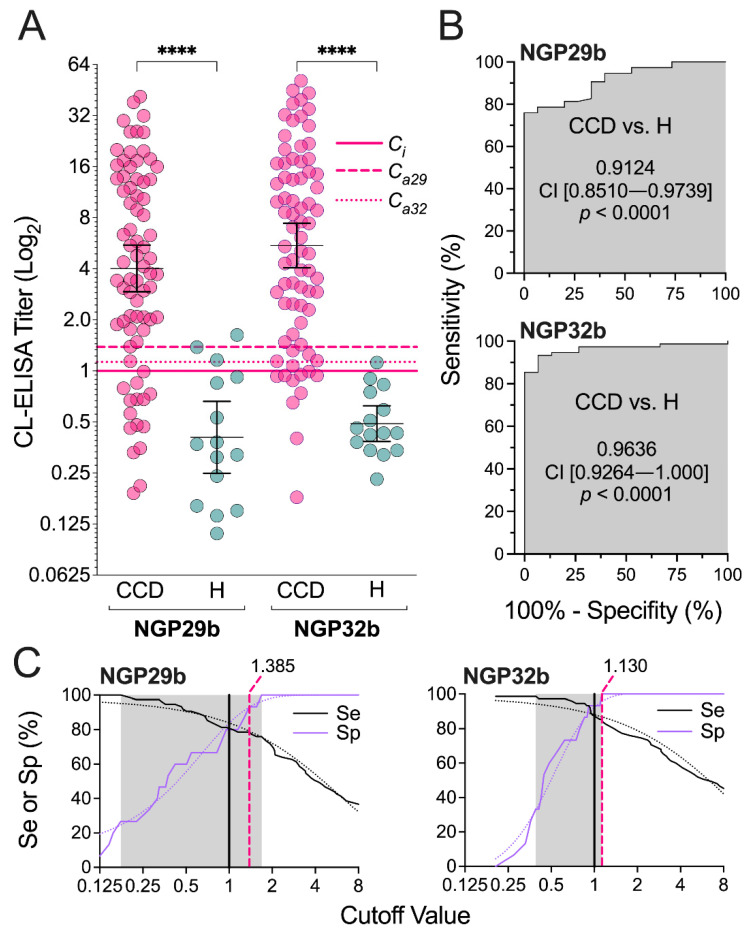
CL-ELISA reactivity of **NGP29b** and **NGP32b** with sera from individual patients with CCD, or from healthy individuals. Sera (at 1:800 dilution) from individual patients with CCD, or from healthy (H) individuals were evaluated against **NGP29b**, and **NGP32b**, each at 50 ng/well. CL-ELISA was performed as described in the Supplementary Material. (**A**) Grouped scatter plot analysis of sera from CCD patients, or H individuals with **NGP29b** or **NGP32b**. Solid red line, *C_i_*, initial cutoff value (titer = 1.000), calculated as described in the Supplementary Material. Discontinued red line (*C_a29_*), adjusted cutoff value for **NGP29b**; dotted red line (*C_a32_*), adjusted cutoff value for **NGP32b**; these cutoffs were calculated based on the ROC and TG-ROC curve analysis data in (**B**,**C**), below. Statistical analysis: Nonparametric Mann–Whitney test; significance level: *p* < 0.05. (**B**) ROC curves for **NGP29b**, and **NGP32b**, comparing the reactivity of sera from CCD infections with H individuals, using the data depicted in the scatter plots (**A**). AUC, area under the curve is indicated (grey area). In brackets, 95% confidence interval (CI) values are indicated. (**C**) TG-ROC curve analysis was performed by plotting the ROC data (**B**) for sensitivity (Se) and specificity (Sp), as described by Greiner et al. [51]. Shaded area indicates the cutoff value interval where Se or Sp could reach 100%. The Se (black) and Sp (purple) raw data points are represented as thick lines, whereas the fitted data are indicated as fine lines. Vertical black lines, initial titer cutoff value (*C_i_ =* 1.000); vertical dotted red line, adjusted cutoff values.

Individual CCD serum samples of 75 patients from the city of Salta, Argentina, and 15 sera from healthy individuals from the USA were assayed. Initially, CL-ELISA endpoint titers of individual samples were defined based on a meaningful statistical method [50], which was originated from each immunoassay microtiter plate by using nine technical replicates of NHSP as negative control to increase accuracy, with a confidence interval (CI) (1–α) ranging 95.0 to 99.9%. In our analyses, a CI = 99.5% with a standard deviation multiplier (*f*) of 3.537 were chosen to calculate the cutoff for each immunoassay plate (see Supplementary Material). Each individual sample was tested in triplicate. Based on the CL-ELISA data using individual CCD patient sera and individual NHS sera (Figure 3A), **NGP29b** diagnosed as positive 60/75 (80.0% sensitivity), whereas **NGP32b** diagnosed as positive 65/75 (86.7% sensitivity) (Table 1 and Table 2). Using a titer cut-off value of 1.000, **NGP29b** and **NGP32b** exhibited a specificity of 80.0% and 93.3%, respectively. To compare the sensitivities and specificities of **NGP29b** and **NGP32b**, receiver-operating characteristic (ROC) curves were plotted (Figure 3B). The area under curve (AUC) values of the ROC curves are 0.9124 and 0.9636 for **NGP29b** and **NGP32b**, respectively, indicating that **NGP32b** exhibited a higher sensitivity and specificity than **NGP29b**. Following a method developed by Greiner et al. [51], the initially selected titer cutoff value of 1.000 (C*_i_*; Figure 3A) for **NGP29b** and **NGP32b** was fine-tuned by performing a two-graph ROC (TG-ROC) analysis. The ROC data (Figure 3B) for sensitivity (Se) and specificity (Sp) were plotted against the cutoff value (Figure 3C). This TG-ROC plot allows the selection of a cutoff value that best matches the selectivity and specificity needs of the assay. In the case of BMK discovery for CD, there is a need for determining treatment efficacies by serology. A higher specificity of the assay is needed to distinguish antibody responses due to the presence from residual *T. cruzi* trypomastigotes from a weak serum cross-reactivity that is observed with NHS, and that is expected after CD patients have been completely cured.

For **NGP29b**, when the titer cutoff value is adjusted to 1.385, only 59/75 (78.7% sensitivity) CCD patients are correctly diagnosed, but the specificity of the assay increases to 93.3% (Table 1 and Table 2). In case of **NGP32b**, an adjustment of the CL-ELISA titer value to 1.130 correctly diagnosed 64/75 (85.3% sensitivity) CCD patients, and the specificity increases to 100% (Table 1 and Table 2). These data show that **NGP32b**, in particular, has an excellent potential as a diagnostic serological BMK. Moreover, the sensitivity and specificity performances of **NGP32b** suggest that the terminal Gal*f*β1,3Man*p*α1,2[Gal*f*β1,3]Man*p*α tetrasaccharide is an immunodominant glycotope present in *T. cruzi* trypomastigote cell surface glycoconjugates, most likely in the abundant GIPLs and GPI moieties of major glycoproteins like tGPI-mucins [27,52]. The structurally different β-Gal*f*-containing glycans present in the *O*-linked glycans of GPI-mucins from Meta forms and, potentially, TCT forms of certain *T. cruzi* strains, particularly those belonging to the genotype or discrete typing unit (DTU) Tc I [30,34,53], could also elicit anti-β-Gal*f* Abs. Such Abs would be expected to have a different specificity, but they could potentially cross-react with **NGP29b** and **NGP32b**, and contribute to the CL-ELISA signal. One explanation for **NGP32b**’s superior performance, in both sensitivity and specificity, over **NGP29b** could be that **NGP32b** comprises a larger glycotope with two appending β-Gal*f* units, which may be more immunogenic and antigenic than a glycotope that is only half its size. Consequently, the IgG Abs elicited may have a higher titer in the CCD patients’ sera, and/or bind more strongly to the tetrasaccharide glycotope present in **NGP32b**. Another possible explanation could be that CCD patient sera contain Abs that recognize the Gal*f*β1,3Man*p*α glycotope in a certain conformation that may be preferred in the molecular environment of the Gal*f*β1,3Man*p*α1,2[Gal*f*β1,3]Man*p*α tetrasaccharide. 

The CCD sera used in this study stemmed from patients of endemic regions that are geographically far apart. The pooled CCD sera were collected in Venezuela, and the individual CCD sera were collected in Argentina. Different *T. cruzi* DTUs predominate in these areas of sample collection (i.e., TcI in Venezuela and TcV in Argentina) [54]. Since the cross-titration results using pooled sera from Venezuela (Figure 2) perfectly match the CL-ELISA results using individual patient sera from Argentina (Figure 3A), one can assume that galactofuranosylated GPI anchors are common in different *T. cruzi* DTUs. If this proves to be true, new β-Gal*f*-based diagnostic and prognostic tools could potentially be universally applicable throughout Latin America, and even in nonendemic regions like the U.S. and Europe, whose parasite genotype makeup circulating in the *T. cruzi*-infected population usually reflects that of the country of origin in Latin America. For instance, in Spain the majority of CCD patients are originated from Bolivia and are infected with DTU TcV or TcIV [55].

The reversed immunoglycomics approach for BMK discovery, as used in this study, is at its best when exact structural data are absent and/or when the identity or exact size of a glycotope is not known [40]. Through a combination of synthetic chemistry and serology, this approach can provide support for the existence of certain glycotopes within a larger cell surface glycan expressed by an organism. Classical immunoglycomics is a top-down approach that can, in principle, also lead to the identification of glycotopes, but here, it would require the cultivation of *T. cruzi* trypomastigotes at a large scale; a number of glycoproteomics techniques, for example, enzymatic and/or chemical cleavage of glycans from a glycocalyx of enormous heterogeneity and complexity; fluorophore labeling; glycoprofiling by high-resolution mass spectrometry (HR-MS), tandem MS, and nuclear magnetic resonance (NMR) spectroscopy. On the other hand, reversed immunoglycomics is a bottom-up approach that combines the synthesis of suspected glycotopes with conjugating them to a carrier protein to produce NGP antigens, and probing them for antigenicity in serological assays using patient sera [40]. The purpose of protein conjugation is to generate antigens that adhere to the wells of microtiter plates. A strong antibody reactivity, and a large differential to negative control sera is indicative of the presence of a glycotope on the parasite cell surface and can lead to BMK discovery.

The presence of anti-β-Gal*f* Abs in the serum of patients with CCD was first observed by Schnaidman et al. [18]. Those authors showed that human CCD sera and a rabbit anti-*T. cruzi* epimastigote antiserum were able to precipitate galactomannans partially purified from *T. cruzi* epimastigotes (*Tc*-GM). The binding of both human CCD and anti-*T. cruzi* epimastigote rabbit sera to *T. cruzi* eGM could be completely abolished (100%) by incubation with 40 μM βGal*f*(1,3)-methyl-α-D-Man*p*, clearly indicating that the *T. cruzi* GM preparation contained the same βGal*f*(1,3)α-D-Man epitope found in the GIPLs (formerly, LLPG). That study also demonstrated that preparations of *Aspergillus fumigatus* GM (*Af*-GM) and galactoglucomannans from *Dactylium dendroides* (*Dd*-GGM), both known to contain various terminal nonreducing β-Gal*f* epitopes, could partially cross-react with human CCD sera and a rabbit anti-*T. cruzi* epimastigote antiserum [18,32]. Subsequently, Golgher et al. showed that a rabbit antiserum raised against purified LLPG (=GIPLs) could strongly bind to *T. cruzi* epimastigote lysates from ten different strains and clones (Y, G, CL-14, CA1, Tulahuén, Ana, Basileu, M226, DM28, and YuA), and at least to one lysate from TCTs of Y strain in western blot, indicating a widespread distribution of the β-Gal*f* epitopes in the parasites from distinct genotypes and geographical origins. The major bands recognized were LPPG/GIPLs and mucins (then known as bands ABC) in epimastigotes, and LPPG/GIPLs and GP80–90 kDa in TCTs. At the same time, Almeida et al. [33] showed that an organic extract (known as fraction F1), obtained from the TCT stage and enriched with βGal*f*-containing GIPLs (based on the reactivity to an antiserum from a rabbit immunized with purified LPPG from *T. cruzi* epimastigotes), was recognized by 100% (*n* = 115) of the sera from a cohort of untreated CCD patients, in a CL-ELISA. Interestingly, patients (*n* = 28) subjected to BZN chemotherapy but still positive for the conventional serology (CS) while negative for the trypanolytic antibody assay (TAA), thus named *dissociated* patients [56,57,58,59], showed much lower reactivity to F1, with 4/28 (14%) already negative for F1 by CL-ELISA. Meanwhile, all treated and cured patients (exhibiting both negative CS and TAA) (*n* = 5) were negative for F1, whereas all treated but not cured patients (*n* = 15) were positive for F1. Taken together, these previous studies confirmed the immunodominance of anti-βGal*f* Abs among CCD patients [12,18,20,60] and open the possibility of using purified TCT GIPLs or synthetic βGal*f*-containing NGPs for accurate diagnosis and early assessment of the chemotherapy of CD.

Finally, while similar reversed immunoglycomics approaches were previously applied by us and others to develop diagnostic and prognostic tools for CD, these research endeavors focused on NGPs that display *T. cruzi*-derived α-Gal*p* moieties [47,48,49,61,62]. However, an extensive validation of all potential BMKs discovered by this method is necessary using large patient sera panels and large panels of control sera of healthy individuals or patients with heterologous diseases.

## 3. Materials and Methods

### 3.1. Synthesis of Oligosaccharides

Allyl 2,3,5,6-tetra-*O*-benzoyl-β-D-galactofuranosyl-(1→3)-4,6-*O*-benzylidene-α-D-mannopyranoside (**3**). A solution of acceptor **2** (215 mg, 0.70 mmol, 3.25 equiv) and donor **1** (151 mg, 0.22 mmol, 1.0 equiv) in anhydrous DCM (25 mL) was added to a 100 mL round bottomed flask with freshly activated MS (4 Å) and stirred under argon for 30 min at 0 °C. NIS (121 mg, 0.54 mmol, 2.5 equiv) and AgOTf (3.3 mg, 0.013 mmol, 0.06 equiv) were added to the mixture, which was stirred for 15 min at 0 °C, and gradually brought to rt. After 3 h of stirring at rt, the reaction mixture was quenched with Et_3_N, followed by filtration and washing of the MS with DCM. The filtrate was washed with a saturated solution of Na_2_S_2_O_3_ and brine. The organic layer was dried over MgSO_4_, concentrated, and purified by column chromatography on silica gel (Hexanes/EtOAc 3:2) to give disaccharide **3** (133 mg, 70%), as a light-yellow oil. R*_f_* 0.37 (Hexanes/EtOAc 3:2). [α]_D_^29^ = +13.35 (c = 0.1 in CHCl_3_). ^1^H NMR (400 MHz, CDCl_3_, 300 K) δ 8.06–7.99 (m, 4H, arom.); 7.97–7.92 (m, 2H, arom.); 7.85–7.81 (m, 2H, arom.); 7.64–7.28 (m, 13H, arom.); 7.25–7.13 (m, 4H, arom.); 5.99–5.85 (m, 2H, H*f*-5, H-b); 5.56 (dd, *J* = 5.5, 0.9 Hz, 1H); 5.50 (s, 1H, OC*H*Ph); 5.44 (s, 1H, H*f*-1); 5.43 (d, *J* = 1.5 Hz, 1H); 5.35–5.27 (m, 1H, H-c); 5.23 (dd, *J* = 10.3, 1.4 Hz, 1H, H-c); 5.00 (d, *J* = 1.1 Hz, 1H, H*m*-1); 4.74 (dd, *J* = 5.6, 3.1 Hz, 1H); 4.57 (dd, *J* = 12.1, 8.4 Hz, 1H, H-a); 4.37–3.78 (m, 9H, H-a, H*f*-6a,b, H*m*-6a,b); 2.94 (s, 1H, -O*H*); ppm. ^13^C NMR (101 MHz, CDCl_3_, 300 K) δ 166.0 (C=O); 165.9 (C=O); 165.7 (C=O); 165.5 (C=O); 137.3 (*C*q, arom.); 133.6 (C-b); 133.5 (*C*-arom.); 133.4 (*C*-arom.); 133.1 (*C*-arom.); 132.9 (*C*-arom.); 130.0 (*C*-arom.); 129.9 (*C*-arom.); 129.8 (*C*-arom.); 129.7 (*C*-arom.); 129.6 (*C*q, arom.); 129.5 (*C*q, arom.); 129.1 (*C*-arom.); 129.0 (*C*q, arom.); 128.6 (*C*q, arom.); 128.5 (*C*-arom.); 128.4 × 2 (*C*-arom.); 128.3 × 2 (*C*-arom.); 125.9 (*C*-arom.); 118.1(C-c); 102.5 (C*f*-1); 102.1 (OC*H*Ph); 99.2 (C*m*-1); 82.5; 81.3; 77.2; 76.8; 71.9; 70.1; 68.9 (CH_2_); 68.7; 68.3 (CH_2_); 64.2 (C-a); 63.7 ppm. ESI-TOF HRMS *m*/*z* calcd for C_50_H_46_O_15_Na [M+Na]^+^ 909.2734, found 909.2706.

(*S*-Acetyl)-3-thiopropyl 2,3,5,6-tetra-*O*-benzoyl-β-D-galactofuranosyl-(1→3)-α-D-mannopyranoside **(4)**. To a solution of the partially deprotected allyl disaccharide **S1** (45 mg, 0.056 mmol, 1.0 equiv) and AIBN (10 mg, 0.061 mmol, 1.1 equiv) in anhydrous THF (9.0 mL), AcSH (28 μL, 0.40 mmol, 7.1 equiv) was added and stirred under argon for 5 min. The solution was then placed in a Rayonet UV reactor and illuminated at 350 nm under stirring and water cooling for 6 h. The solution was concentrated, co-evaporated with toluene, and purified by preparative TLC (DCM/MeOH 20:1) to yield thioester **4** as a yellow oil (39 mg, 79%). R*_f_* 0.30 (DCM/MeOH 20:1). ^1^H NMR (400 MHz, CDCl_3_, 300 K) δ 8.08 (d, *J* = 7.5 Hz, 2H, arom.); 8.04–7.95 (m, 4H, arom.); 7.90 (d, *J* = 7.3 Hz, 2H, arom.); 7.59–7.49 (m, 4H, arom.); 7.45–7.28 (m, 8H, arom.); 6.03–5.93 (m, 1H, H*f*-5); 5.72 (dd, *J* = 5.7, 1.5 Hz, 1H); 5.56–5.50 (m, 1H); 5.49 (s, 1H, ***H*-1-Gal*f***); 4.93 (dd, *J* = 5.6, 3.6 Hz, 1H); 4.85 (s, 1H, ***H*-1-Man**); 4.82–4.70 (m, 2H); 4.10–3.92 (m, 3H); 3.85 (d, *J* = 3.4 Hz, 2H); 3.75–3.56 (m, 3H); 3.45–3.28 (m, 2H); 3.20 (br. s. 1H); 2.91 (t, *J* = 7.0 Hz, 2H); 2.30 (s, 3H, C*H*_3_); 1.82 (quin, *J* = 6.6 Hz, 2H) ppm.^13^C NMR (101 MHz, CDCl_3_, 300 K) δ 195.8 (SC=OCH_3_); 166.2 (C=O); 166.0 (C=O); 165.63 (C=O); 165.56 (C=O); 133.6 (*C*H, arom.); 133.5 (*C*H, arom.); 133.4 (*C*H, arom.); 133.2 (*C*H, arom.); 129.94 (*C*H, arom.); 129.89 (*C*H, arom.); 129.84 (*C*H, arom.); 129.75 (*C*H, arom.); 129.4 (*C*, arom.); 129.3 (*C*, arom.); 128.7 (*C*, arom.); 128.51 (*C*H, arom.); 128.47 (*C*, arom.); 128.44 (*C*H, arom.); 128.42 (*C*H, arom.); 128.35 (*C*H, arom.); 104.2 (***C*-1-Gal*f***); 99.5 (***C*-1-Man**); 83.0 (*C*H); 81.0 (*C*H); 78.6 (*C*H); 77.2 (*C*H); 72.0 (*C*H); 70.1 (*C*H); 68.3 (*C*H); 66.01 (*C*H); 65.9 (*C*H_2_); 63.1 (*C*H_2_); 62.4 (*C*H_2_); 30.6 (*C*H_3_); 29.2 (*C*H_2_); 25.8 (*C*H_2_) ppm. ESI-TOF HRMS *m*/*z* calcd for C_45_H_46_O_16_SNa [M+Na]^+^ 897.2404, found 897.2718.

3-Thiopropyl β-D-galactofuranosyl-(1→3)-α-D-mannopyranoside [G29_SH_ and (G29_S_)_2_]. To a flask containing thioester **4** (29 mg, 0.033 mmol, 1.0 equiv), 3 mL of 0.5 M NaOMe in MeOH was added under argon, and stirred at rt for 2 h. HRMS showed complete removal of the protecting groups. The mixture was neutralized with Amberlyst 15 ion-exchange resin and filtered through Celite. After removal of the MeOH by rotary evaporation the remainder was dissolved in water and lyophilized. Initially, the 3-thiopropyl disaccharide **G29_SH_** was produced, which oxidized by handling on air to the disulfide within hours. The disulfide **(G29_S_)_2_** was obtained as a white powder (14 mg, quant.). R*_f_* 0.33 (EtOAc/EtOH/H_2_O/NH_3_ 7:3:1:0.5). ^1^H NMR (400 MHz, D_2_O, 300 K) δ 5.13 (d, *J* = 1.7 Hz, 1H); 4.90 (d, *J* = 1.8 Hz, 1H); 4.13–4.16 (m, 2H); 4.10–4.04 (m, 2H); 3.92 –3.61 (m, 10H); 2.88–2.84 (m, 2H, OCH_2_CH_2_*CH_2_*S); 2.06–2.00 (m, 2H, OCH_2_C*H_2_*CH_2_S) ppm.^13^C NMR (101 MHz, D_2_O,300 K) δ 106.9; 99.7; 83.0; 81.4; 77.1; 75.6; 72.8; 70.8; 66.8; 66.1; 65.1; 62.9; 61.0; 34.9; 28.2 ppm. ESI-TOF HRMS *m*/*z* calcd for C_15_H_28_O_11_S [M+Na]^+^ 439.1250, found 439.1214; for C_30_H_54_O_22_S_2_Na [M+Na]^+^ 853.2446, found 853.2366.

2,3,5,6-tetra-*O*-benzoyl-β-D-galactofuranosyl-(1→3)-2-acetyl-4,6-*O*-benzylidene-α-D-mannopyranoside trichloroacetimidate (**5**). To a solution of the hemiacetal **S3** (421 mg, 0.47 mmol) in dry DCM (24 mL), CCl_3_CN (1.0 mL, ~20 equiv) and DBU (0.1 mL, ~1.4 equiv) were consecutively added at 0 °C and stirred at rt for 15 min. Then, the residue was concentrated and purified by flash column chromatography on silica gel (Hexanes/EtOAc = 2:1) to yield trichloroacetimidate **5** as a light-yellow powder (425 mg, 87%). [α]_D_^29^ = −20.02 (c = 0.1 in CHCl_3_). R*_f_* 0.40 (Hexanes/EtOAc = 2:1). ^1^H NMR (400 MHz, CDCl_3_, 300 K) δ 8.74 (s, 1H, NH); 8.02 (d, *J* = 7.8 Hz, 2H, arom.); 7.95 (m, 4H, arom.); 7.87 (d, *J* = 7.8 Hz, 2H, arom.); 7.58–7.42 (m, 4H, arom.); 7.40–7.22 (m, 10H, arom.); 7.20–7.09 (m, 3H, arom.); 6.26 (s, 1H, H*m*-1); 5.97 (dt, *J* = 7.7 Hz, 3.8 Hz, 1H, H*f*-5); 5.64–5.56 (m, 2H); 5.52, (d, *J* = 5.4 Hz, 1H); 5.48–5.44 (m, 2H); 4.77 (dd, *J* = 5.1 Hz, 3.4 Hz, 1H); 4.64 (dd, *J* = 11.7 Hz, 8.0 Hz, 1H); 4.52 (dd, *J* = 9.5 Hz, 3.7 Hz, 1H); 4.40–4.24 (m, 2H); 4.19–4.03 (m, 2H); 3.93–3.81 (m, 1H); 2.25 (s, 3H, COC*H_3_*) ppm. ^13^C NMR (101 MHz, CDCl_3_, 300 K) δ 169.8 (C=O); 165.9 (C=O); 165.6 (C=O); 165.5 (C=O); 165.1 (C=O); 160.1 (C=N); 136.8 (*C*q, arom.); 133.4 (*C*-arom.); 133.3 (*C*-arom.); 133.1 (*C*-arom.); 133.0 (*C*-arom.); 129.9 (*C*-arom.); 129.8 (*C*-arom.); 129.7 (*C*-arom.); 129.5 (*C*q, arom.); 129.4 (*C*q, arom.); 129.1 (*C*-arom.); 129.0 (*C*q, arom.); 128.9 (*C*q, arom.); 128.4 × 3 (*C*-arom.); 128.2 (*C*-arom.); 125.8 (*C*-arom.); 102.3; 101.9; 95.7; 90.5 (*C*q); 81.8; 81.5; 77.6; 77.2; 76.4; 69.9; 68.9; 68.4 (CH_2_); 66.9; 66.3; 63.4 (CH_2_); 20.7 (CH_3_) ppm. ESI-TOF HRMS *m*/*z* calcd for C_51_H_44_Cl_3_NO_16_Na [M+Na]^+^ 1054.1623, found 1054.1650.

Allyl 2,3,5,6-tetra-*O*-benzoyl-β-D-galactofuranosyl-(1→3)-2-O-acetyl-4,6-*O*-benzylidene-α-D-mannopyranoside-(1→2)-[2,3,5,6-tetra-*O*-benzoyl-β-D-galactofuranosyl-(1→3)]-4,6-*O*-benzylidene-α-D-mannopyranoside **(6)**. A solution of disaccharide acceptor 3 (125 mg, 0.14 mmol) and disaccharide donor 5 (215 mg, 0.22 mmol) in anhydrous DCM (30 mL) with freshly activated MS (4 Å) was stirred under Ar for 30 min at 0 °C. Then, BF_3_-OEt_2_ (10.0 μL, 0.08 mmol) was added to the flask, which was stirred at 0 °C for 15 min, and gradually brought to rt. After 30 min stirring at rt, the reaction mixture was quenched with Et_3_N and then filtered. The filtrate was concentrated and purified by column chromatography on silica gel (Hexanes/EtOAc = 3:2) to afford tetrasaccharide 6 as a white powder (185 mg, 75%). [α]_D_^29^ = −31.70 (c = 0.1 in CHCl_3_). R*_f_* 0.53 (Hexanes/EtOAc = 3:2). ^1^H NMR (400 MHz, CDCl_3_, 300 K) δ 8.08–8.00 (m, 6H, arom.); 7.99–7.92 (m, 6H, arom.); 7.84–7.74 (m, 4H, arom.); 7.56–7.45 (m, 7H, arom.); 7.39–7.28 (m, 15H, arom.); 7.25–7.15 (m, 12H, arom.); 6.01 (dt, *J* = 7.7, 3.8 Hz, H*f*-5, 1H); 5.97–5.85 (m, 2H); 5.80 (dd, *J* = 3.6, 1.2 Hz, 1H); 5.59 (s, 1H); 5.51–5.57 (m, 3H); 5.49 (d, *J* = 5.5, 1H); 5.44 (s, 1H); 5.36–5.23 (m, 5H); 4.84 (d, *J* = 1.0, 1H); 4.77 (dd, *J* = 5.2, 3.4 Hz, 1H); 4.71 (dd, *J* = 11.7, 8.2 Hz, 1H); 4.64 (dd, *J* = 5.4, 2.7 Hz, 1H); 4.56–4.49 (m, 2H); 4.41–3.97 (m, 11H + 2H from EtOAc); 3.85–3.94 (m, 3H); 2.12 (s, 3H) ppm. ^13^C NMR (101 MHz, CDCl_3_, 300 K) δ 169.9 (C=O); 166.0 (C=O); 165.9 (C=O); 165.7 (C=O); 165.6 (C=O); 165.5 (C=O); 165.1 (C=O); 137.3 (*C*q, arom.); 137.1 (*C*q, arom.); 133.4 × 2 (*C*-arom.);133.3 × 2 (*C*-arom.); 133.2 (*C*-arom.); 133.1 (*C*-arom.); 133.0 (*C*-arom.); 132.9 (*C*-arom.); 132.8 (*C*-arom.); 130.0 (*C*-arom.); 129.9 × 3(*C*-arom.); 129.8 × 2 (*C*-arom.); 129.7 × 2 (*C*-arom.); 129.6 (*C*q, arom.); 129.5 (*C*q, arom.); 129.2 (*C*q, arom.); 129.1 (*C*q, arom.); 129.0 (*C*-arom.); 128.9 (*C*q, arom.); 128.8 (*C*q, arom.); 128.3 (*C*arom.); 128.2 (*C*-arom.); 128.1 (*C*-arom.); 126.0 (*C*-arom.); 125.8 (*C*-arom.); 117.9 (CH_2_); 102.5 (CH); 102.4 (CH); 102.0 (CH); 101.9 (CH); 101.4 (CH); 99.0 (CH); 82.4 (CH); 81.7 (CH); 81.6 (CH); 81.5 (CH); 77.7 (CH); 77.5 (CH); 77.2 × 2 (CH); 76.8 (CH); 75.1 (CH); 71.4 (CH); 70.2 (CH); 70.1 (CH); 69.2 (CH); 68.7 (CH_2_); 68.1 (CH_2_); 67.8 (CH); 64.4 (CH_2_); 64.3 (CH); 64.1(CH); 63.6 (CH_2_); 60.4 (CH2); 29.7 (CH); 21.1 (CH); 20.8 (CH_3_); 14.2 (CH) ppm. ESI-TOF HRMS: *m*/*z* [M+Na]^+^ calcd for C_99_H_88_O_30_Na 1779.5258, found 1779.5445.

3-Thiopropyl β-D-galactofuranosyl-(1→3)-α-D-mannopyranoside-(1→2)-[β-D-galactofuranosyl-(1→3)]-α-D-mannopyranoside [G32_SH_ and (G32_S_)_2_]. To a flask containing thioester S5 (72 mg, 0.043 mmol), 13 mL of 0.5 M NaOMe in MeOH was added under Ar, and the mixture was stirred at rt for 2 h. HRMS showed complete removal of the protecting groups. The reaction mixture was neutralized with Amberlyst-15 ion-exchange resin, and filtered through Celite. The MeOH was removed by rotary evaporation, and the remainder was dissolved in water and lyophilized. Initially, the unprotected 3-thiopropyl disaccharide G32_SH_ was produced, which oxidized to the disulfide by handling on air within hours. (G32_S_)_2_ was obtained as a white powder (25 mg, 75%). ^1^H NMR (400 MHz, D_2_O) δ 5.15 (dd, *J* = 5.5, 1.8 Hz, 2H); 5.08 (dd, *J* = 8.5, 1.9 Hz, 2H); 4.25–4.24 (m, 1H); 4.18–3.59 (m, 25H); 2.85 (t, 2H, OCH_2_CH_2_C*H_2_*S); 2.06–1.98 (m, 2H, OCH_2_C*H_2_*CH_2_S) ppm. ^13^C NMR (101 MHz, D_2_O) δ 104.9; 104.6; 101.7; 98.4; 82.9; 82.8; 81.40; 81.37; 77.0; 76.8; 75.8; 75.4; 74.7; 73.3; 72.96; 70.76; 70.7; 66.9; 66.2; 65.3; 65.2; 62.9; 61.13; 61.12; 60.9; 34.9; 28.1 ppm. ESI-TOF HRMS: *m*/*z* [M+Na]^+^ calcd for C_54_H_94_O_42_S_2_Na 1501.4559, found 1501.4542.

### 3.2. Conjugation Protocol

The kit for the conjugation of the thiol-containing glycans **(G29_S_)_2_**, or **(G32_S_)_2_**, to BSA (Imject™ Maleimide-Activated BSA, Thermo Fisher Scientific, Waltham, MA, catalog #77116), was purchased from Thermo Fisher Scientific, and the conjugation procedure followed was similar to the one described by the manufacturer and previously published [61]. A 0.05 M TCEP solution was prepared by diluting neutral TCEP solution (Bond-Breaker TCEP solution, 0.5 M, Thermo Fisher Scientific, Waltham, MA, catalog #77720) with conjugation buffer provided in the kit (83 mM sodium phosphate buffer, 0.1 M EDTA, 0.9 M sodium chloride, 0.02% sodium azide, pH 7.2). 0.9 Equiv of the diluted TCEP solution was added to a 1.5 mL micro-centrifuge tube that contained disulfide **(G29_S_)_2_** (0.6 mg, 3.0 μmol) or **(G32_S_)_2_** (1 mg, 3.0 μmol), and the mixture was agitated on a shaker for 30 min to furnish thiol **G29_SH_** or **G32_SH_**, respectively. The maleimide-activated BSA (2 mg, 15–25 moles of maleimide/mole BSA) was reconstituted with 200 μL of conjugation buffer to produce a 10 mg/mL solution. The disaccharide solution was added to the reconstituted BSA and agitated at rt for 2–3 h. The conjugation mixture was diluted with HPLC grade water to a volume of 1 mL and desalted using an Amicon Ultra 3K centrifugal filter and was centrifuged for 20 min at 4000× *g*, rt. The mixture was washed with 1 mL of water 3 times following the same centrifugation procedure. The tube with the filtrate was then removed, and 500 µL of water was added to the **NGP29b** or **NGP32b** solution remaining in the filter. Since a small amount of aggregation can occur, the solution/suspension was transferred onto a 2-mL ZebaTM spin desalting column (7K MWCO), provided in the kit, that was previously washed with 1 mL of water 4 times and centrifuged at 1000× *g* for 2 min at rt. This procedure removed all salts and aggregated protein. The filtrate was lyophilized and can be stored at −50 °C for at least 6 months. In our hands, this combination of filtration and size exclusion chromatography avoids or minimizes aggregation of the NGP. To determine the **NGP29b** or **NGP32b** quantity, a solution of 1–2 mg of it in 1–3 mL of ultrapure water was prepared, and the concentration was determined with a Pierce BCA Protein Assay Reagent kit using a spectrophotometer at a detection wavelength of 562 nm. To determine the payload (average number of **G29_SH_** or **G32_SH_** per BSA molecule) comparative MALDI-TOF MS spectra were measured, see Supplementary Material).

### 3.3. Chemiluminescent ELISA

The detailed chemiluminescent ELISA (CL-ELISA) protocol is described in the Supplementary Material.

## 4. Conclusions

Using a reversed immunoglycomics approach, a combination of the chemical synthesis of suspected *T. cruzi*-derived β-Gal*f*-containing glycotopes and their immunological evaluation with sera of CCD patients led to the discovery of two novel BMKs for CD (i.e., **NGP29b** and **NGP32b**). Of the two BMKs studied, **NGP32b** performed particularly well in distinguishing chronic Chagas disease sera from the sera of healthy individuals displaying sensitivity and specificity values of 85.3% and 100%, respectively. These data suggest that the branched tetrasaccharide Gal*f*β1,3Man*p*α1,2[Gal*f*β1,3]Man*p*α present in **NGP32b** is an immunodominant glycotope most likely present in at least one of the GPI anchors of mucins from the mammal-dwelling trypomastigote form of *T. cruzi*. β-Galactofuranosylation of core GPI anchors could result in common motifs in *T. cruzi* trypomastigotes, which could be the basis for the development of new diagnostic and potentially prognostic tools for CD.

## Figures and Tables

**Table 1:** CL-ELISA reactivity of sera from CCD patients and healthy individuals with **NGP29b** and **NGP32b**.

Disease/Control	*n*	NGP29b		NGP32b	
		Positive	Negative	Positive	Negative
		Pre-TG-ROC Analysis			
CCD	75	60	15	65	10
H	15	3	12	1	14
		Post-TG-ROC Analysis			
**CCD**	75	59	16	64	11
**H**	15	1	14	0	15

**Table 2:** Sensitivity and specificity of **NGP29b** and **NGP32b**.

Parameter	NGP29b		NGP32b	
	Pre-TG-ROC	Post-TG-ROC	Pre-TG-ROC	Post-TG-ROC
	%			
Sensitivity	80.0	78.7	86.7	85.3
Specificity	80.0	93.3	93.3	100

## Data Availability

Some of the results described here have been reported in Alba L. Montoya’s dissertation, 2020, The University of Texas at El Paso.

## Supplementary Materials

The following supporting information can be downloaded online. List of abbreviations; Scheme S1, Synthesis of **G29** (3-thiopropyl Galβ*f*1,3Manα) (overall reaction scheme); Scheme S2, Synthesis of **G32** (3-thiopropyl Galβ*f*1,3Manα1,2-[Galβ*f*1,3]Manα) (overall reaction scheme); Figure S1, MALDI-TOF mass spectra of **NGP29b** and **NGP32b**; detailed description of the syntheses of reaction intermediates **S1**; **S2**; **S3**; **S4**; **S5**; description of the CL-ELISA protocol (including titer cutoff value calculation); Appendix with NMR and additional MS spectra of compounds **3**; **4**; **5**; **6**; **S1**; **S2**; **S3**; **S4**; **S5**; **G29_SH_**/**(G29_S_)_2_**; and **G32_SH_**/**(G32_S_)_2_**.

## Sample Availability

The NGPs used in this study could be made available for collaborative studies. However, owing to the very limited amount of all serum samples used here, unfortunately these samples will not be available for sharing or collaborative studies.
