# Supplementary material for: Specific Recognition of β-Galactofuranose-Containing Glycans of Synthetic Neoglycoproteins by Sera of Chronic Chagas Disease Patients
Source: Molecules. 2022 Jan 9;27(2):411. doi: 10.3390/molecules27020411 (PMC8781757; doi:10.3390/molecules27020411)

SUPPLEMENTARY MATERIAL

Specific Recognition of β-Galactofuranose-Containing Glycans of Synthetic Neoglycoproteins by Sera of Chronic Chagas Disease Patients

Alba L. Montoya, [1#] Eileni R. Gil [1#], Emily L. Heydemann [1], Igor L. Estevao [2], Bianca E. Luna [2], Cameron C. Ellis [2], Sohan R. Jankuru [1], Belkisyolé Alarcón de Noya [3], Oscar Noya [3,4], Maria Paola Zago [5], Igor C. Almeida, [2,5,*] and Katja Michael [1,5,*]

[1] Department of Chemistry and Biochemistry, Border Biomedical Research Center, University of Texas at El Paso, El Paso, TX, 79968, USA
[2] Department of Biological Sciences, Border Biomedical Research Center, University of Texas at El Paso, El Paso, TX, 79968, USA
[3] Sección de Inmunología, Instituto de Medicina Tropical, Facultad de Medicina, Universidad Central de Venezuela, Caracas 1041-A, Venezuela
[4] Centro para Estudios Sobre Malaria, Instituto de Altos Estudios "Dr. Arnoldo Gabaldón", Instituto Nacional de Higiene Rafael Rangel, Ministerio del Poder Popular para la Salud, Caracas 1041-A, Venezuela
[5] Instituto de Patología Experimental, Facultad de Ciencias de la Salud, Universidad Nacional de Salta (UNSa)-Consejo Nacional de Investigaciones Científicas y Técnicas (CONICET), 4400 Salta, Argentina
[#] These authors contributed equally.

Table of Contents

GENERAL INFORMATION S1

ABBREVIATIONS USED S2

SYNTHETIC SCHEMES, PROCEDURES, AND CHARACTERIATIONS S4

Synthesis of the G29 (3-thiopropyl Galβ*f*1,3Manα), overall synthetic scheme S4

Synthesis of the intermediate S1 .... S4

Synthesis of G32 (3-thiopropyl Galβ*f*1,3Manα1,2-[Galβ*f*1,3]Manα), overall synthetic scheme S6

Synthesis of the intermediate S2 .... S7

Synthesis of the intermediate S3 .... S8

Synthesis of the intermediate S4 .... S9

Synthesis of the intermediate S5 .... S10

MALDI-TOF MS of NGP29b and NGP32b S11

CHEMILUMINESCENT ENZYME-LINKED IMMUNOSORBENT ASSAY (CL-ELISA) S10

APPENDIX (^{1}H and ^{13}C NMR spectra, and additional mass spectra) S15

^{1}H NMR spectrum, 400 MHz, $CDCl_3$, compound 3 .... S16

^{13}C NMR, 100 MHz spectrum, $CDCl_3$, compound 3 .... S17

HSQC NMR spectrum, 400 MHz, $CDCl_3$, compound 3 .... S18

COSY NMR spectrum, 400 MHz, $CDCl_3$, compound 3 .... S19

ESI-TOF HR mass spectrum of compound 3 .... S20

^{1}H NMR spectrum, 400 MHz, $CDCl_3$, compound S1 .... S21

^{13}C NMR spectrum, 100 MHz, $CDCl_3$, compound S1 .... S22

ESI-TOF HR mass spectrum of compound S1 .... S23

^{1}H NMR spectrum, 400 MHz, $CDCl_3$, compound 4 .... S24

^{13}C NMR spectrum, 100 MHz, $CDCl_3$, compound 4 .... S25

ESI-TOF HR mass spectrum of compound 4 .... S26

^{1}H NMR spectrum, 400 MHz, D_2O, compound $G29_{SH}$ [and $(G29_S)_2$] S27

^{13}C NMR spectrum, 100 MHz, D_2O, compound $G29_{SH}$ [and $(G29_S)_2$] S28

ESI-TOF HR mass spectrum of compounds G29$_{SH}$ and (G29$_{S}$)$_2$ S29
^{1}H NMR spectrum, 400 MHz, $CDCl_3$, compound S2 .... S30
^{13}C NMR spectrum, 100 MHz, $CDCl_3$, compound S2 .... S31
ESI-TOF HR mass spectrum of compound S2 .... S32
^{1}H NMR spectrum, 400 MHz, $CDCl_3$, compound S3 .... S33
^{13}C NMR spectrum, 100 MHz, $CDCl_3$, compound S3 .... S34
ESI-TOF HR mass spectrum of compound S3 .... S35
^{1}H NMR spectrum, 400 MHz, $CDCl_3$, compound 5 .... S36
^{13}C NMR spectrum, 100 MHz, $CDCl_3$, compound 5 .... S37
ESI-TOF HR mass spectrum of compound 5 .... S38
^{1}H NMR spectrum, 400 MHz, $CDCl_3$, compound 6 .... S39
^{13}C NMR spectrum, 100 MHz, $CDCl_3$, compound 6 .... S40
ESI-TOF HR mass spectrum of compound 6 .... S41
^{1}H NMR spectrum, 400 MHz, $CDCl_3$, compound S4 .... S42
^{13}C NMR spectrum, 100 MHz, $CDCl_3$, compound S4 .... S43
ESI-TOF HR mass spectrum of compound S4 .... S44
^{1}H NMR spectrum, 400 MHz, $CDCl_3$, compound S5 .... S45
^{13}C NMR spectrum, 100 MHz, $CDCl_3$, compound S5 .... S46
ESI-TOF HR mass spectrum of compound S5 .... S47
^{1}H NMR spectrum, 400 MHz, D_2O, compound G32$_{SH}$ [and (G32$_{S}$)$_2$] S48
^{13}C NMR spectrum, 100 MHz, D_2O, compound G32$_{SH}$ [and (G32$_{S}$)$_2$] S49
ESI-TOF HR mass spectrum of compounds G32$_{SH}$ and (G32$_{S}$)$_2$ S50

GENERAL INFORMATION

All chemicals were purchased as reagent grade from Thermo Fisher Scientific, Sigma-Aldrich, or Acros Organic, and used without further purification. The ACS grade solvents used for reactions were obtained from Thermo Fisher Scientific and they were distilled from the appropriate drying agents. Molecular sieves (3 Å and 4 Å) were purchased from Alfa Aesar and Thermo Fisher Scientific, respectively, and activated under high vacuum and heat prior to use. Reactions were performed under an argon atmosphere, strictly anhydrous conditions and monitored by TLC on silica gel 60 F254 plates from EMD Millipore or Dynamic Adsorbents, Inc. Spots were detected under UV light (254 nm) and/or by charring with 4% sulfuric acid in ethanol. The purification of the compounds was performed by flash column chromatography on silica gel (40-60 μm) from Thermo Fisher Scientific, and the ratio between silica and crude product ranged from 50:1 to 120:1 (dry w/w). FPLC purifications were performed with an AKTA Purifier 100 FPLC system from Cytiva (former GE Healthcare) using a Resource RPC column with a stationary phase of 15 ◎m polystyrene/divinylbenzene beads, solvent A: 2% CH_3CN/H_2O; solvent B: 85% CH_3CN/H_2O. ^{1}H and ^{13}C NMR spectra were recorded on a Bruker Avance III HD 400 MHz NMR spectrometer at 400 and 101 MHz or on a JEOL 600 MHz NMR spectrometer at 600 and 150 MHz, respectively. Chemical shifts (in ppm) were determined relative to tetramethylsilane (δ 0.00 ppm) as an internal standard in $CDCl_3$ and CD_3OD, or relative to the $CDCl_3$ signal (δ 77.0 ppm) in ^{13}C NMR spectra. In case of spectra measured in D_2O, a solution of tetramethylsilane in $CDCl_3$ in a sealed capillary was used as an external standard for calibration. Coupling constant(s) [Hz] were measured from one-dimensional 1H-NMR spectra. Full or partial assignments were made by 1D spectra as well as standard COSY, HSQC, and TOCSY experiments. In disaccharides and tetrasaccharides, protons of mannose are labeled with an italized "*m*", and protons of galactofuranose with an italized "*f*". Protons in the allyl group are labeled as "a" for the sp^3-hybridized CH_2, "b" for the sp^2-hybridized CH, and "c" for the terminal sp^2-hybridized CH_2. MS analyses of the carbohydrate derivatives were performed on a high-resolution JEOL AccuTOF mass spectrometer using an electrospray ionization (ESI) source. The thiol-ene reactions were performed in a Rayonet RPR200 photochemical reactor (Southern New England Ultraviolet Company, Branford, CT) equipped with 16 UV lamps (350 nm). Bovine serum albumin (BSA) and BSA derivatives (neoglycoproteins (NGPs) and 2-mercaptoethanol-BSA) were measured by matrix-assisted laser/desorption/ionization mass spectrometer (MALDI)-TOF-MS (MALDI-8020, Shimadzu) using 10 mg/mL sinapinic acid, 0.1% trifluoroacetic acid, in 50% acetonitrile as a matrix. Polystyrene Nunc MaxiSorp 96-well ELISA plates, and chemiluminescent ELISA reagents were purchased from Thermo Fisher Scientific or Jackson ImmunoResearch, and chemiluminescence was recorded on a Luminoskan Ascent,

Thermo Fisher Scientific. Optical rotations were measured on an ATAGO AP-300 Automatic Polarimeter.

ABBREVIATIONS USED

Å	angstrom
Abs	antibodies
Ac	acetyl
Ac2O	acetic anhydride
AcSH	thiolacetic acid
AEP	aminoethylphosphonate
AgOTf	silver trifluoromethanesulfonate
AIBN	azobisisobutyronitrile
All	allyl
AllOH	allyl alcohol
Ar	argon
AUC	area under the curve
$BF_3{\cdot}Et_2O$	boron trifluoride etherate
BMKs	biomarkers
BSA	bovine serum albumin
Bz	benzoyl
BzCl	benzoyl chloride
BZN	benznidazole
C_a	adjusted cutoff value
C_i	initial cutoff value
CCD	chronic Chagas disease
CCDSP	chronic Chagas disease serum pool
CCl_3CN	trichloroacetonitrile
CD	Chagas disease
CL-ELISA	chemiluminescent enzyme-linked immunosorbent assay
CS	conventional serology
DBU	1,8-diazabicyclo[5.4.0]undec-7-ene
DCM	methylene dichloride
DMAP	4-Dimethylaminopyridine
DPAP	2,2-dimethoxy-2-phenylacetophenone
ELISA	enzyme-linked immunosorbent assay
equiv.	equivalent
ESI-TOF HRMS	Electrospray ionization Time-of-Flight high resolution mass spectra
Et_3N	triethylamine

EtOAc	ethyl acetate
EtSH	ethanethiol
FPLC	Fast protein liquid chromatography
GM	galactomannan
GIPL	glycoinositolphospholipid
h	hour(s)
HOAc	acetic acid
HR-MS	high resolution mass spectrometry
IgG	immunoglobulin G
LFA	lateral flow assay
LOD	limit of detection
LPPG	lipopeptidoglycan
MALDI-TOF	matrix-assisted laser desorption ionization time-of-flight
MeOH	methanol
min	minute(s)
MS	molecular sieves
m/z	mass-to-charge ratio
NGP	neoglycoprotein
NHS	normal human serum
NHSP	normal human serum pool
NIS	N-iodosuccinimide
NMR	nuclear magnetic resonance
PBS	phosphate-buffered saline
PI	phosphatidylinositol
PTLC	preparative thin-layer chromatography
PTSA	para-toluenesulfonic acid
quant.	quantitative
ROC	receiver-operating characteristic
RLU	relative luminescence unit(s)
rt	room temperature
tBu	tert-butyl
TAA	trypanolytic antibody assay
TCEP	tris(2-carboxyethyl)phosphine
Tc	*T. cruzi*
TCT	*T. cruzi* trypomastigote
TFA	trifluoroacetic acid
TG-ROC	two graph-receiver-operating characteristic
TLC	thin-layer chromatography
TolSH	para-thiocresol
THF	tetrahydrofuran

SYNTHETIC SCHEMES, PROCEDURES, AND CHARACTERIATIONS

Synthesis of G29 (3-thiopropyl Galβ*f*1,3Manα)

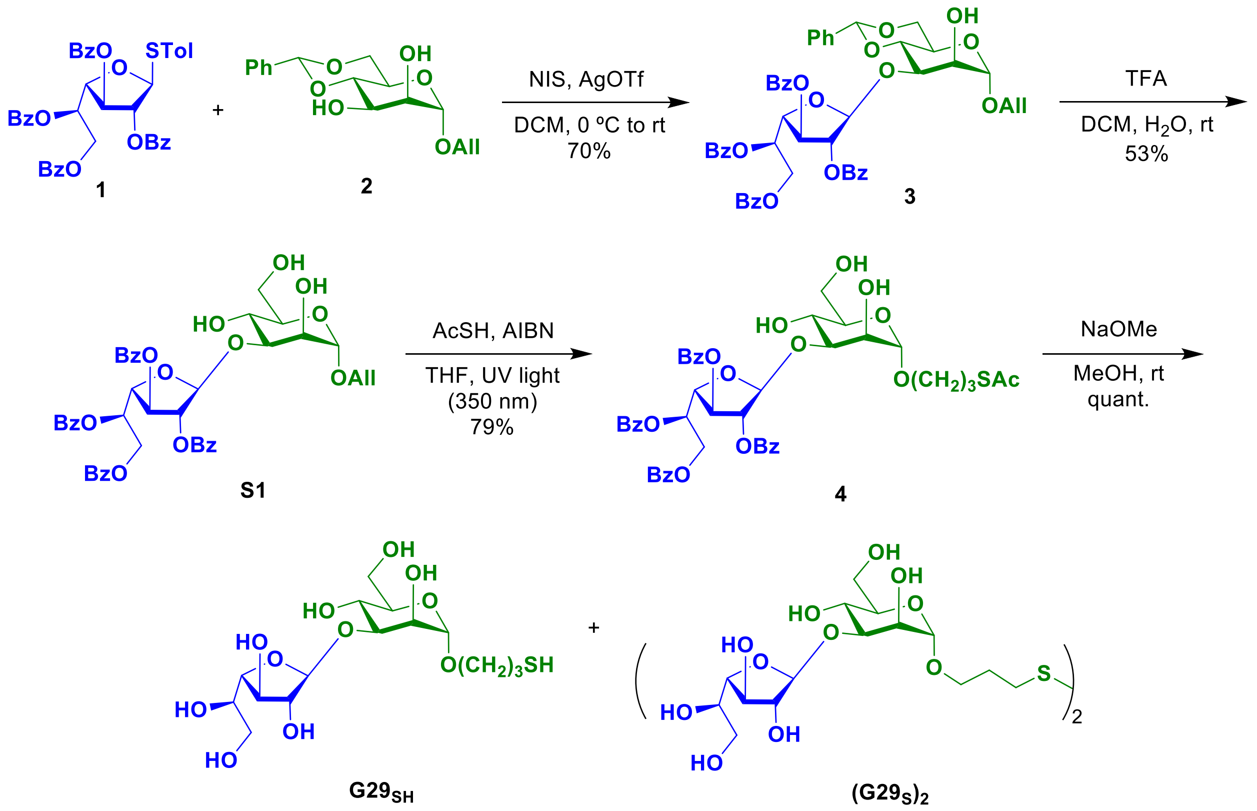

Scheme S1. Synthesis of the 3-thiopropyl disaccharide **G29SH**.

Synthesis of the intermediate S1

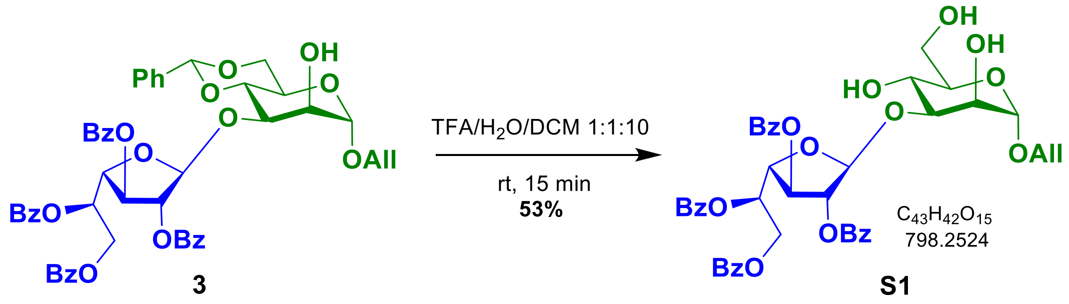

Allyl 2,3,5,6-tetra-*O*-benzoyl-β-D-galactofuranosyl-(1→3)-α-D-mannopyranoside (**S1**). Compound **3** (94 mg, 0.11 mmol, 1.0 equiv) was dissolved in DCM (15 mL). While stirring at r.t., H_2O (1.5 mL) and TFA (1.5 mL) were sequentially added, and the reaction proceeded for 20 min. After the starting material was observed to disappear on TLC, the resulting solution was co-evaporated with EtOH (10 mL) twice. The solution was concentrated, dried under vacuum pressure, and purified by prep-TLC (DCM/MeOH 15:1) to yield **S1** (45 mg, 53%) as a beige powder. R_f 0.37 (DCM/MeOH 15:1). $[\alpha]_D^{29}$= -8.34 (c = 0.1 in $CHCl_3$). ^1H NMR (400 MHz, $CDCl_3$, 300K) δ 8.12–8.05 (m, 2H, arom.); 8.04–7.96 (m, 4H, arom.); 7.94–7.88 (m, 2H, arom.); 7.61–7.49 (m, 4H, arom.); 7.46–7.28 (m, 8H, arom.); 5.99–5.92 (m, 1H, H*f*-5); 5.92–5.79 (m, 1H, H-b); 5.72 (dd, *J* = 5.7, 2.0 Hz, 1H); 5.50 (d, *J* = 1.6 Hz, 1H); 5.47 (s, 1H, H*f*-1); 5.27 (dd, *J* = 17.2, 1.5 Hz, 1H, H-c); 5.19 (dd, *J* = 10.3, 1.3 Hz, 1H, H-c); 4.93 (d, *J* = 1.2 Hz, 1H, H*m*-1); 4.90 (dd, *J* = 5.7, 3.8 Hz, 1H); 4.9–4.83 (m, 2H); 4.21–3.80 (m, 7H); 3.72–3.61 (m, 1H); 3.21 (br. s., 1H, O*H*); 2.94 (br. s., 1H, O*H*); 2.31 (br. s., 1H, O*H*) ppm. ^{13}C NMR (101 MHz, $CDCl_3$, 300K) δ 166.3 (C=O); 166.1 (C=O); 165.7 (C=O); 165.6 (C=O); 133.7 (C-b); 133.5 (*C*-arom.); 133.4 (*C*-arom.); 133.2 (*C*-arom.); 130.0 (*C*-arom.); 129.9 (*C*-arom.); 129.8 (*C*-arom.); 129.7 (*C*-arom.); 129.4 (*C*q, arom.); 129.3 (*C*q, arom.); 128.7 (*C*q, arom.); 128.6 (*C*-arom.); 128.5 × 2 (*C*-arom.); 128.4 (*C*-arom.); 117.9(C-c); 104.4 (C*f*-1); 98.6 (C*m*-1); 83.0; 81.2; 78.9; 77.0; 71.9; 70.2; 68.5; 68.2 (CH_2); 66.3; 63.1 (CH_2); 62.6 (CH_2) ppm. ESI-TOF HRMS *m*/*z* calcd for $C_{43}H_{42}O_{15}$ $[M+Na]^+$ 821.2421, found 821.2452.

Synthesis of G32 (3-thiopropyl Galβ*f*1,3Manα1,2-[Galβ*f*1,3]Manα)

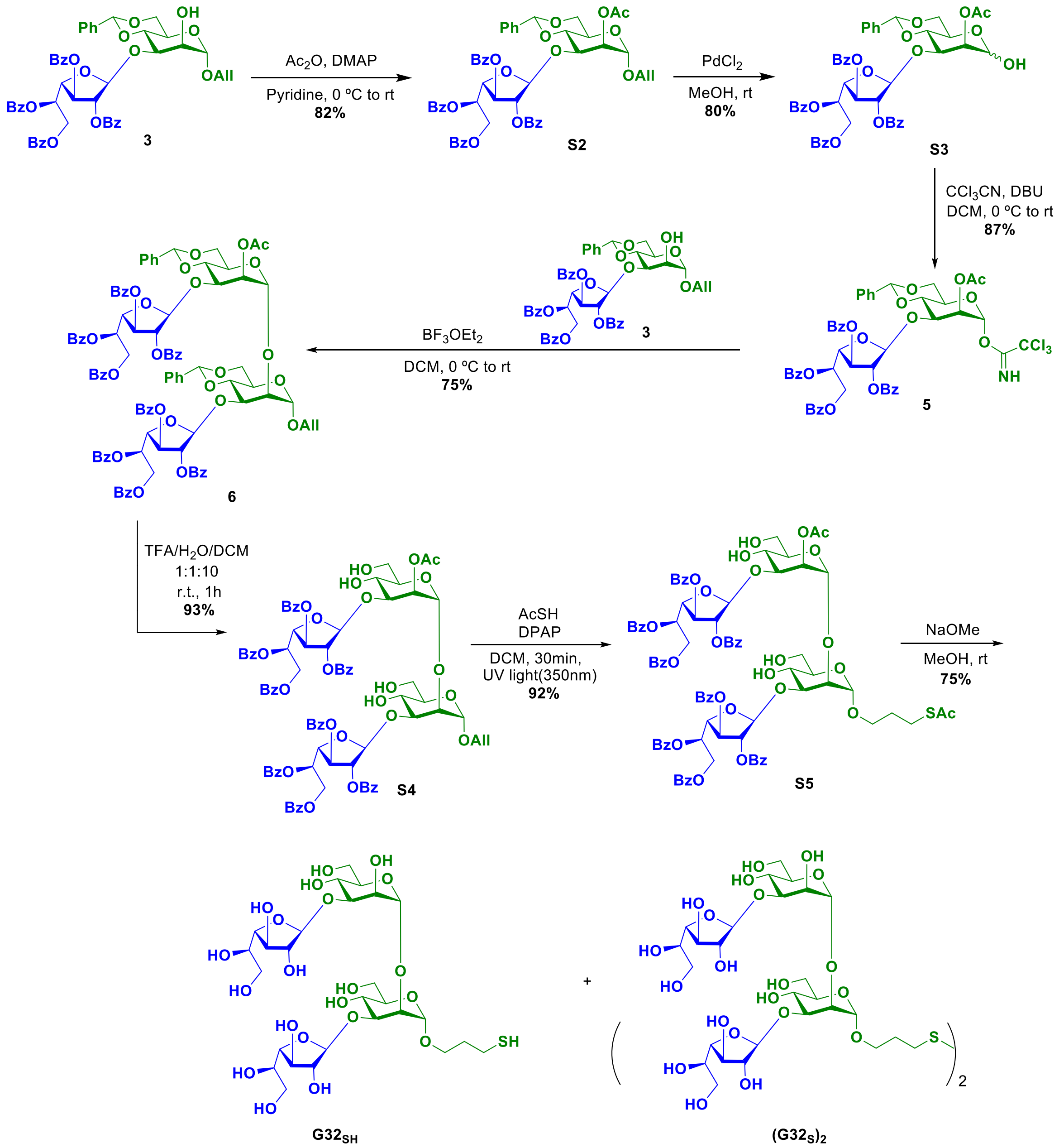

Scheme S2. Synthesis of the 3-thiopropyl disaccharide **G32**$_{SH}$.

Synthesis of the intermediate S2

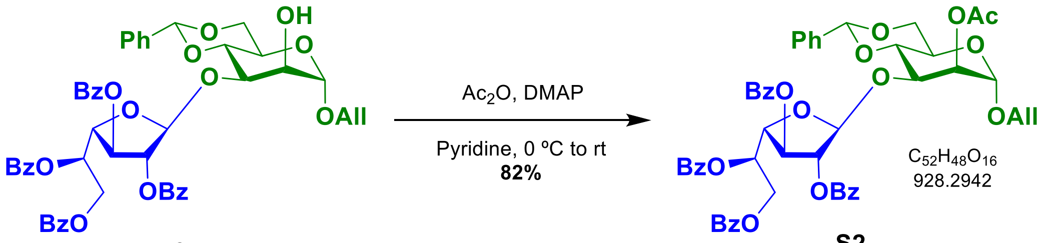

Allyl 2,3,5,6-tetra-*O*-benzoyl-β-D-galactofuranosyl-(1→3)-2-acetyl-4,6-*O*-benzylidene-α-D-mannopyranoside (**S2**). To a solution of disaccharide **3** (717 mg, 0.81 mmol) in dry pyridine (48 mL) at 0°C, acetic anhydride (4.0 mL) and DMAP (40 mg) were added. The mixture was stirred at 0°C for 30 min, allowed to warm to rt and stirred for 3 h. The mixture was washed with water and extracted with EtOAc. The organic layers were dried over $MgSO_4$, concentrated, and purified by column chromatography on silica gel (Hexanes/EtOAc = 3:1) to afford **S2** (616 mg, 82%), as a light-yellow powder. $[\alpha]_D^{29}$ = +1.67 (c = 0.1 in $CHCl_3$). R_f 0.57 (Hexanes/EtOAc = 3:2). ^{1}H NMR (400 MHz, $CDCl_3$, 300K) δ 8.04–7.99 (m, 2H, arom.); 7.98–7.92 (m, 4H, arom.); 7.88–7.82 (m, 2H, arom.); 7.57–7.43 (m, 4H, arom.); 7.38–7.29 (m, 6H, arom.); 7.25–7.12 (m, 6H, arom.); 5.94 (m, 1H, H*f*-5); 5.89 (m, 1H, H-b); 5.53 (s, 1H); 5.48 (d, *J* = 5.4 Hz, 1H); 5.45–5.40 (m, 3H); 5.31 (dq, *J* = 17.2, 1.5 Hz, 1H); 5.23 (dq, *J* = 10.3, 1.3 Hz, 1H); 4.87 (d, *J* = 1.5 Hz, 1H); 4.71 (dd, *J* = 5.3, 3.0 Hz, 1H); 4.62 (dd, *J* = 12.0, 8.2 Hz, 1H); 4.45 (dd, *J* = 9.7, 3.7 Hz, 1H); 4.35–4.25 (m, 2H); 4.19 (ddt, *J* = 12.8, 5.2, 1.5, 1.5 Hz, 1H); 4.05–3.90 (m, 3H); 3.88–3.80 (m, 1H); 2.21 (s, 3H,-COCH_3) ppm.^{13}C NMR (101 MHz, $CDCl_3$, 300K) δ 170.1 (C=O); 166.0 (C=O); 165.6 (C=O); 165.4 (C=O); 165.1 (C=O); 137.1 (Cq, arom.); 133.3 (C-b); 133.2 (*C*-arom.); 133.1 (*C*-arom.); 132.9 (*C*-arom.); 130.0 (*C*-arom.); 129.9 (*C*-arom.); 129.8 (*C*-arom.); 129.7 (*C*-arom.); 129.6 (Cq, arom.); 129.5 (Cq, arom.); 129.1 (*C*-arom.); 129.0 (Cq, arom.); 128.9 (Cq, arom.); 128.3 × 4 (*C*-arom.); 128.2 (*C*-arom.); 125.8 (*C*-arom.); 118.3 (C-c); 102.2; 101.9; 97.8; 81.6; 81.4; 77.7; 77.2; 70.0; 69.1; 68.7 (CH_2); 68.6; 68.4 (CH_2); 63.9 (CH_2); 63.8; 20.9 (CH_3) ppm. ESI-TOF HRMS *m*/*z* calcd for $C_{52}H_{48}O_{16}$ $[M+Na]^+$ 951.2840, found 951.2842.

Synthesis of the intermediate S3

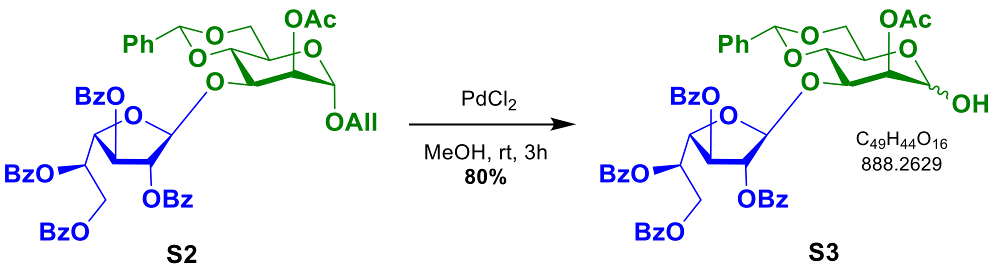

Hydroxyl 2,3,5,6-tetra-*O*-benzoyl-β-D-galactofuranosyl-(1→3)-2-acetyl-4,6-*O*-benzylidene-α-D-mannopyranoside (**S3**). To a solution of fully protected disaccharide (**S2**) (571 mg, 0.62 mmol) in dry MeOH (48 mL), $PdCl_2$ (72 mg) was added. The mixture was stirred at rt for 3 h. The mixture was filtered through celite, while washed with MeOH. The filtrate was evaporated under vacuum and purified by column chromatography on silica gel (Hexanes/EtOAc = 3:2) to afford **S3** (437 mg, 80%), as a light-yellow powder. R_f 0.30 (Hexanes/EtOAc = 3:2). ^{1}H NMR (400 MHz, $CDCl_3$, 300K) δ 8.05–7.99 (m, 2H, arom.); 7.98–7.92 (m, 4H, arom.); 7.85 (dd, *J* = 8.3, 1.2 Hz, 2H, arom.); 7.58–7.43 (m, 4H, arom.); 7.40–7.27 (m, 10H, arom.); 7.19–7.10 (m, 3H, arom.); 5.93 (dt, *J* = 7.8, 3.3 Hz, 1H); 5.55–5.39 (m, 5H); 5.24 (dd, *J* = 3.8, 1.1 Hz, 1H); 4.71 (dd, *J* = 5.4, 2.9 Hz, 1H); 4.61 (dd, *J* = 11.9, 8.1 Hz, 1H); 4.50 (dd, *J* = 10.0, 3.7 Hz, 1H); 4.34 (dd, *J* = 11.9, 3.7 Hz, 1H); 4.26 (dd, *J* = 10.1, 4.8 Hz, 1H); 4.20–4.08 (m, 1H); 4.06–3.97 (m, 1H); 4.06–3.97 (m, 1H); 3.77–3.86 (m, 1H); 3.14 (d, J = 4.0 Hz, 1H); 2.21 (s, 3H,- $COCH_3$) ppm.^{13}C NMR (101 MHz, $CDCl_3$, 300K) δ 170.2 (C=O); 166.1 (C=O); 165.7 (C=O); 165.5 (C=O); 165.2 (C=O); 137.1 (Cq, arom.); 133.4 (*C*-arom.); 133.2 (*C*-arom.); 133.1 (*C*-arom.); 133.0 (*C*-arom.); 130.0 (*C*-arom.); 129.9 (*C*-arom.); 129.8 (*C*-arom.); 129.7 (*C*-arom.); 129.6 (Cq, arom.); 129.5 (Cq, arom.); 129.1 (*C*-arom.); 129.0 (Cq, arom.); 128.9 (Cq, arom.); 128.3 (*C*-arom.); 128.2 (*C*-arom.); 125.9 (*C*-arom.); 102.2; 101.9; 93.7; 81.7; 81.4; 77.8; 77.1; 70.0; 68.9; 68.8 (CH_2); 68.7; 63.9 (CH_2); 63.8; 20.9 (CH_3) ppm. ESI-TOF HRMS: *m*/*z* $[M+Na]^+$ calcd for $C_{49}H_{44}O_{16}$ 911.2527, found 911.2520.

An alternative deallylation method that produced compound **S3** at 95% yield utilized $Pd[PPh_3]_4$ in HOAc at 80°C, following a procedure described in Nakayama, K. et al., *Chem. Pharm. Bull.* **1992**, *40*, 1718-1720.

Synthesis of the intermediate S4

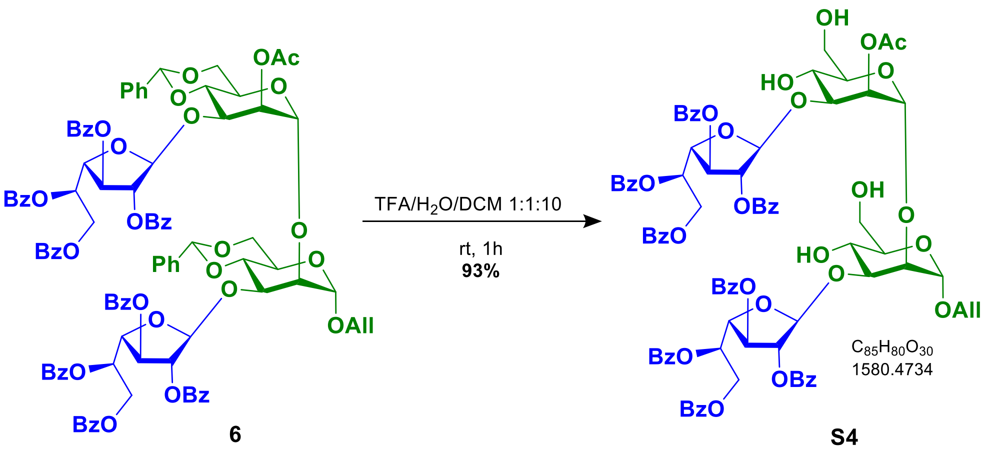

Allyl 2,3,5,6-tetra-*O*-benzoyl-β-D-galactofuranosyl-(1→3)-2-acetyl-α-D-mannopyranoside-(1→2)-[2,3,5,6-tetra-*O*-benzoyl-β-D-galactofuranosyl-(1→3)]-α-D-mannopyranoside (**S4**). Fully protected tetrasaccharide **6** (165 mg, 0.93 mmol) was dissolved in DCM (15 mL). While stirring at rt, H_2O (1.3 mL) and TFA (1.3 mL) were sequentially added, and the reaction proceeded for 1 h. After the starting material was observed to disappear on TLC, the reaction mixture was quenched by addition of Et_3N. The solution was concentrated, dried under vacuum pressure, and purified by column chromatography on silica gel (Hexanes /EtOAc = 1:2) to yield **S4** (127 mg, 85%) as a white powder. $[\alpha]_D^{29}$= -26.02 (c = 0.1 in $CHCl_3$) R_f 0.23 (Hexanes/EtOAc= 1:2). ^1H NMR (400 MHz, $CDCl_3$) δ 8.10–7.96 (m, 12H, arom.); 7.88–7.83 (m, *J* = 11.2, 6.9, 1.4 Hz, 4H, arom.); 7.60–7.27 (m, 21H, arom.); 7.25–7.21 (m, 3H, arom.); 6.09-6.01 (m, 2H); 5.89–5.80 (m,1H); 5.68 (dd, *J* = 5.8, 1.8 Hz, 1H); 5.61 (s, 1H, 1H-anomeric), 5.60–5.58 (m, 1H); 5.52–5.48 (m, *J* = 14.7, 4.5, 1.8 Hz, 3H); 5.41 (s, 1H-anomeric); 5.29 (d, *J* = 1.9 Hz, 1H, 1H-anomeric), 5.28–5.17 (m, 2H-OCH_2-CH=CH_2); 4.89 (dd, *J* = 5.7, 3.4 Hz, 1H); 4.80–4.68 (m, 5H+1H-anomeric); 4.18–3.58 (m, 15H); 3.05 (d, *J* = 3.5 Hz, 1H, OH); 2.87 (s, 1H, OH); 1.92 (s,3H, -COCH_3). ^{13}C NMR (151 MHz, $CDCl_3$) δ169.9 (-O*C*=OCH_3); 166.4 (C=O); 166.2 (C=O); 165.8 (C=O); 165.7 (C=O), 165.7 (C=O); 165.6 (C=O); 165.3 (C=O); 133.7 (*C*-arom.); 133.5 (*C*-arom.); 133.4 (*C*-arom.); 133.3 × 3 (*C*-arom.); 133.1 (*C*-arom.); 130.0 (*C*-arom.); 129.97 (*C*-arom.); 129.93 (*C*-arom.); 129.90 (*C*-arom.); 129.85 (*C*-arom.); 129.8 (*C*-arom.); 129.54 (*C*q-arom.); 129.45 (*C*q-arom.); 129.3 (*C*q-arom.); 129.2 (*C*q-arom.); 128.9 (*C*q-arom.); 128.80 (*C*q-arom.); 128.77 (*C*q-arom.); 128.6 (*C*-arom.); 128.44 (*C*-arom.); 128.41 (*C*-arom.); 128.35 (*C*-arom.); 117.8 (OCH_2-CH=CH_2); 104.5 (CH, *C*-anomeric); 103.3 (CH, *C*-anomeric), 99.3 (CH, *C*-anomeric); 97.9 (CH, *C*-anomeric); 82.7 (CH); 82.0 (CH); 81.8 (CH); 81.4 (CH); 77.6 (CH), 74.6 (CH); 74.4 (CH); 72.6(CH); 72.6 (CH); 70.3 (CH); 69.8 (CH); 68.2 (CH); 68.1 (CH_2); 66.7

(CH); 66.3 (CH); 63.2 (CH_2); 63.0 (CH_2); 62.5 (CH_2); 62.3 (CH_2); 20.7 (CH_3). ESI-TOF HRMS: m/z[M+Na]$^+$calcd for $C_{85}H_{80}O_{30}$ 1603.4632, found 1603.4600.

Synthesis of the intermediate S5

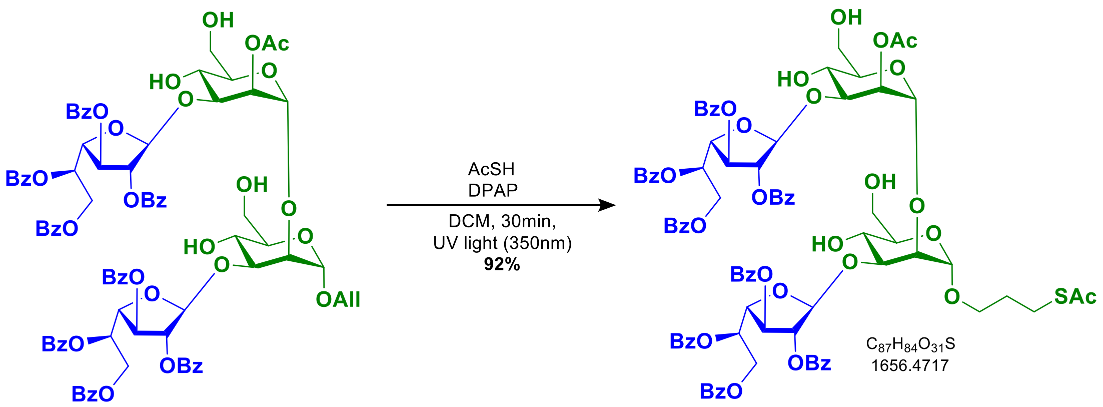

S4 S5

(*S*-Acetyl)-3-thiopropyl-2,3,5,6-tetra-*O*-benzoyl-β-D-galactofuranosyl-(1→3)-2-acetyl-α-D-mannopyranoside-(1→2)-[2,3,5,6-tetra-*O*-benzoyl-β-D-galactofuranosyl-(1→3)]-α-D-

mannopyranoside (**S5**)**.** To a solution of allyl tetrasaccharide **S4** (81 mg, 0.05 mmol) and DPAP (118 µL of a solution of 5 mg DPAP in 500 µL DCM, 0.0038 mmol) in anhydrous DCM (2.8 mL) under Ar, thioacetic acid (16 µL, 0.23 mmol) was added, and the mixture was stirred under water cooling (~ 25 °C) for 30 min in a Rayonet UV reactor equipped with 350 nm lamps. The solution was then co-evaporated with toluene and concentrated to near dryness. The crude product was purified by PTLC on silica gel (Hex/EtOAc = 1:2) to afford the acyl-protected tetrasaccharide **S5** (78 mg, 92%) as a white solid $[\alpha]_D^{29}$= -13.35 (c = 0.1 in $CHCl_3$). R_f 0.22 (Hexanes/EtOAc= 1:2). ^{1}H NMR (400 MHz, $CDCl_3$) δ 8.10–7.96 (m, 11H, arom.); 7.86 (ddd, *J* = 8.8, 7.9, 1.3 Hz, 4H, arom.), 7.60–7.27 (m, 20H, arom.), 7.25–7.21(m, 4H, arom.), 6.09–6.03 (m, 2H), 5.68 (dd, *J* = 5.8, 1.8 Hz, 1H), 5.61–5.58 (m, 1H+1H-anomeric), 5.52-5.48 (m, 3H), 5.43 (s, 1H, 1H-anomeric), 5.30 (d, *J* = 1.7 Hz, 1H, 1H-anomeric), 4.90 (dd, *J* = 5.8, 3.4 Hz, 1H), 4.79–4.67 (m, 5H+1H-anomeric), 4.17 (dd, *J* = 9.6, 3.3 Hz, 1H), 4.07–3.99 (m, 3H), 3.95–3.64 (m, 7H), 3.55–3.51 (m, 1H), 3.36–3.31 (m, 1H), 3.02–2.84 (m, 4H), 2.70 (t, 1H), 2.31 (s, 3H, SCOCH_3), 1.92 (s, 3H, COCH_3), 1.80 (m, *J* = 6.6 Hz, 2H, $OCH_2CH_2CH_2SCOCH_3$).^{13}C NMR (101 MHz, $CDCl_3$) δ196.1 (S*C*=OCH$_3$); 169.9 (O*C*=*O*CH$_3$); 166.4 (C=O); 166.2 (C=O); 165.8 (C=O); 165.7 (C=O); 165.7 (C=O); 165.6 (C=O); 165.3 (C=O); 133.7 (*C*-arom.); 133.4 (*C*-arom.); 133.32 (*C*-arom.); 133.29 (*C*-arom.); 133.26 (*C*-arom.); 133.1 (*C*-arom.); 130.02 (*C*-arom.); 129.96 (*C*-arom.); 129.93 (*C*-arom.); 129.89 (*C*-arom.); 129.84 (*C*-arom.); 129.81 (*C*-arom.); 129.6 (*C*q-arom.); 129.5 (Cq-arom.); 129.3 (Cq-arom.); 129.2 (Cq-arom.); 128.86 (Cq-arom.); 128.84 (Cq-arom.); 128.80 (Cq-arom.); 128.79

(Cq-arom.); 128.6 (*C*-arom.); 128.43 (*C*-arom.); 128.39 (*C*-arom.); 128.34 (*C*-arom.); 128.33 (*C*-arom.); 104.4 (CH, *C*-anomeric); 103.2 (CH, *C*-anomeric); 99.3 (CH, *C*-anomeric); 98.7 (CH, *C*-anomeric); 82.7 (CH); 82.0 (CH); 81.8 (CH); 81.3 (CH); 77.6 (CH); 74.4 (CH); 74.3 (CH); 72.7 (CH); 70.2 (CH); 69.8 (CH); 68.1 (CH); 66.8 (CH); 66.3 (CH); 65.7 (CH_2); 63.2 (CH_2); 63.0 (CH_2); 62.6 (CH_2); 62.3 (CH_2); 30.6 (CH); 29.2 (CH_2); 25.8 (CH_2); 20.7 (CH_3). ESI-TOF HRMS: m/z[M+Na]$^+$calcd for $C_{87}H_{84}O_{31}S$ 1679.4615, found 1679.4639.

MALDI-TOF MS of NGP29b and NGP32b

To determine the mass of BSA and **NGP29b** or **NGP32b**, in a 1.5 mL microcentrifuge tube, 1 μL of a solution of ~ 0.1 mg BSA /100 μL H_2O, was combined with 1 μL of a solution of ~ 0.1 mg **NGP29b** or **NGP32b** /100 μL H_2O and 2 μL of matrix (10 mg/mL sinapinic acid, 50% acetonitrile, 0.1% TFA). Two μL of the combined sample-matrix mixture was spotted onto a 48-well steel MALDI plate and allowed to crystallize at rt for approximately 20 minutes. The mass spectra were acquired using a SHIMADZU MALDI-8020 mass spectrometer set to linear mode with dithering at a scan range of 10,000 to 100,000 m/z. Data acquisition included a laser power of 110, laser rep. rate (Hz) 50, accumulated shots 5, blast shots 2, profiles at 200, pulse extraction set to 66431, and a blanking mass of 15000. Spectra were processed by Threshold Apex set at constant Threshold, Gaussian smoothing, smoothing filter width 200 and peak width 2. BSA standard was used for calibration and internal references set at [BSA+H]$^+$ = 66120 with a 5 ppm mass tolerance.

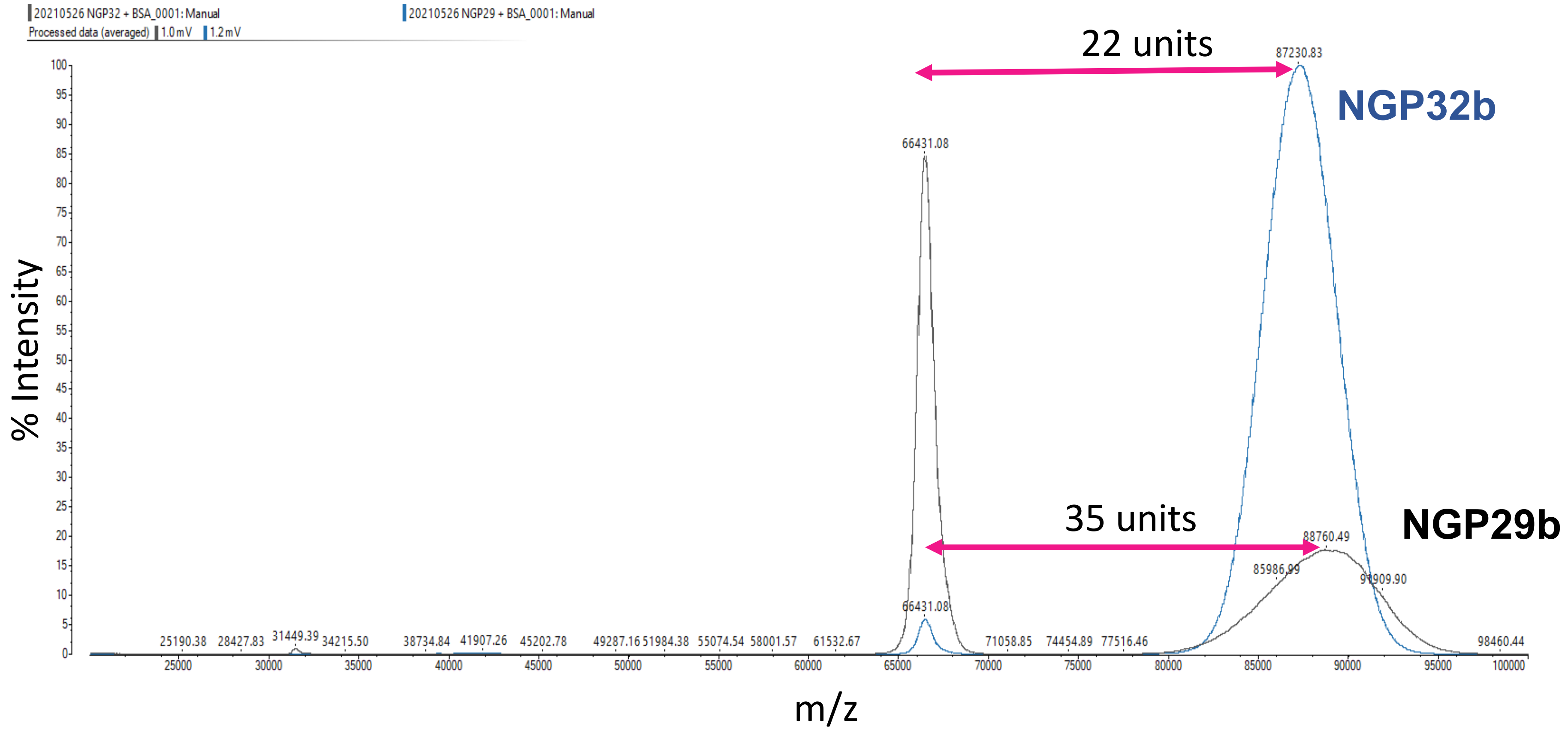

Figure S1. MALDI-TOF mass spectra of **NGP29b** and **NGP32b** overlaid with underivatized BSA. m/z, mass-to-charge ratio.

m/z for BSA $[M+H]^+$ 66431; for **NGP29b** $[M+H]^+$ 88760; **for NGP32b** $[M+H]^+$ 87230. The average payload of **G29**$_{SH}$ units per BSA molecule was 35, and the average payload of **G32**$_{SH}$ units per BSA was 22.

CHEMILUMINESCENT ENZYME-LINKED IMMUNOSORBENT ASSAY (CL-ELISA)

The CL-ELISA was carried out for screening the anti-β-Gal*f* IgG antibody response to **NGP29b** and **NGP32b** in chronic Chagas disease serum pool (CCDSP) (n=10) and normal human serum pool (NHSP) (for cross-titration) (n=10), and in individual samples (n=75) of CCD patients from nonendemic area (Venezuela), and healthy individuals (n=15) from a nonendemic area (USA). In summary, cross-titration of antigen and sera dilutions were performed using 96-well polystyrene microplates (Maxisorp, Nunc, Thermo Scientific). Microplate wells were coated with antigens diluted in 200 mM carbonate-bicarbonate buffer, pH 9.6 (CBB), according to the desired concentration, which ranged from 3.12 to 400 ng/well for cross-titration assays, and at a fixed concentration of 50 ng/well for individual patient serum, for 16 h at 4°C to a final volume of 50 μL/well. Excess antigen was discarded by inverting the microplate and tapping it onto absorbent paper towels, and free sites of the wells were blocked with 200 μL/well PBS-1% BSA (PBS-B) for 1 h at 37°C. The plate was sealed with plastic wrap to avoid evaporation during incubation. Plates were then washed three times with 200 μL PBS-0.05% Tween 20 (Sigma-Aldrich) (PBS-T) per well, using an automatic plate washer (El406 washer, BioTek). For cross-titration (antigen concentration vs. serum dilution) assays, 50 μL/well of CCDSP and NHSP from endemic (Venezuela) and nonendemic (U.S.A.) countries, respectively, were subjected to 1:2 serial dilution (from 1:200 to 1:1600) in PBS-T 1% BSA (PBS-TB). For immunoassays with individual sera from CCD patients from Argentina and NHS controls, sera were diluted 1:800. Serum incubation was performed for 1 h at 37°C, and then microplates were washed three times with PBS-T as above, to remove unbound antibodies. Secondary goat anti-human IgG (H + L) biotinylated antibody (1:5,000 dilution, Cat# 31770, Thermo Fisher Scientific) was prepared in PBS-TB and added to microplates (50 μL/well). The plates were washed again three times with PBS-T, and 50 μL/well of Pierce High Sensitivity NeutrAvidin-HRP (1:5,000 dilution, Cat# 31030, Thermo Fisher Scientific) was added. Microplates were then covered with aluminum foil and incubated for 1 h at 37°C before they were washed three times with PBS-T. The luminescent reaction was measured (as per Relative Luminescence Units-RLU) immediately after addition of SuperSignal™ ELISA Pico Luminol/Enhancer solution, SuperSignal ELISA Pico Substrate (Thermo Fisher Scientific, 37070), and CBB 0.1% BSA, diluted 1:1:8 (v/v/v) to a total volume of 50 μL/well using a microplate reader with luminescence detector (Luminoskan Ascent, Labsystems, Thermo Fisher Scientific). To test the protocol

functionality and assay reactivity of each microplate, positive and negative controls were included in triplicate as well as a blank sample lacking the primary antibody (serum) to address nonspecific background reactivities from the reagents against an internal antigen control, Galα1,3Galβ1,4GlcNAcα-BSA (**NGP24b**), at 100 ng/well. For assays with individual sera, the cutoff in each plate was calculated by using nine technical replicate RLU values of an NHSP (made from 10 different individual NHS). The average RLU value of the nine technical replicates was added to the standard deviation (SD) multiplied by a SD multiplier factor (*f*); thus, cutoff = [NHSP mean] + SD x *f*), as proposed by Frey et al. (A. Frey, J. Di Canzio, D. Zurakowski, *J. Immunol. Methods* **1998**, *221*, 35-41), and more recently described by Montoya et al. (A. L. Montoya, V. M. Austin, S. Portillo, I. Vinales, R. A. Ashmus, I. Estevao, S. R. Jankuru, Y. Alraey, W. S. Al-Salem, Á. Acosta-Serrano, I. C. Almeida, K. Michael, *JACS Au* **2021**, *1*, 1275-1287). The CL-ELISA titer was defined as the ratio of the experimental sample's average RLU value to the cutoff value. A serum sample was considered positive when its titer was equal to or higher than 1.000 and negative when the titer was lower than 1.000 (initial cutoff value, C*i*). For both NGPs (**NGP29b** and **NGP32b**) the initial cutoff values were fine-tuned to the adjusted cutoff values, as described in the main text of this publication.

APPENDIX (^{1}H and ^{13}C NMR spectra, and additional mass spectra)

¹H NMR spectrum, 400 MHz, $CDCl_3$, compound 3

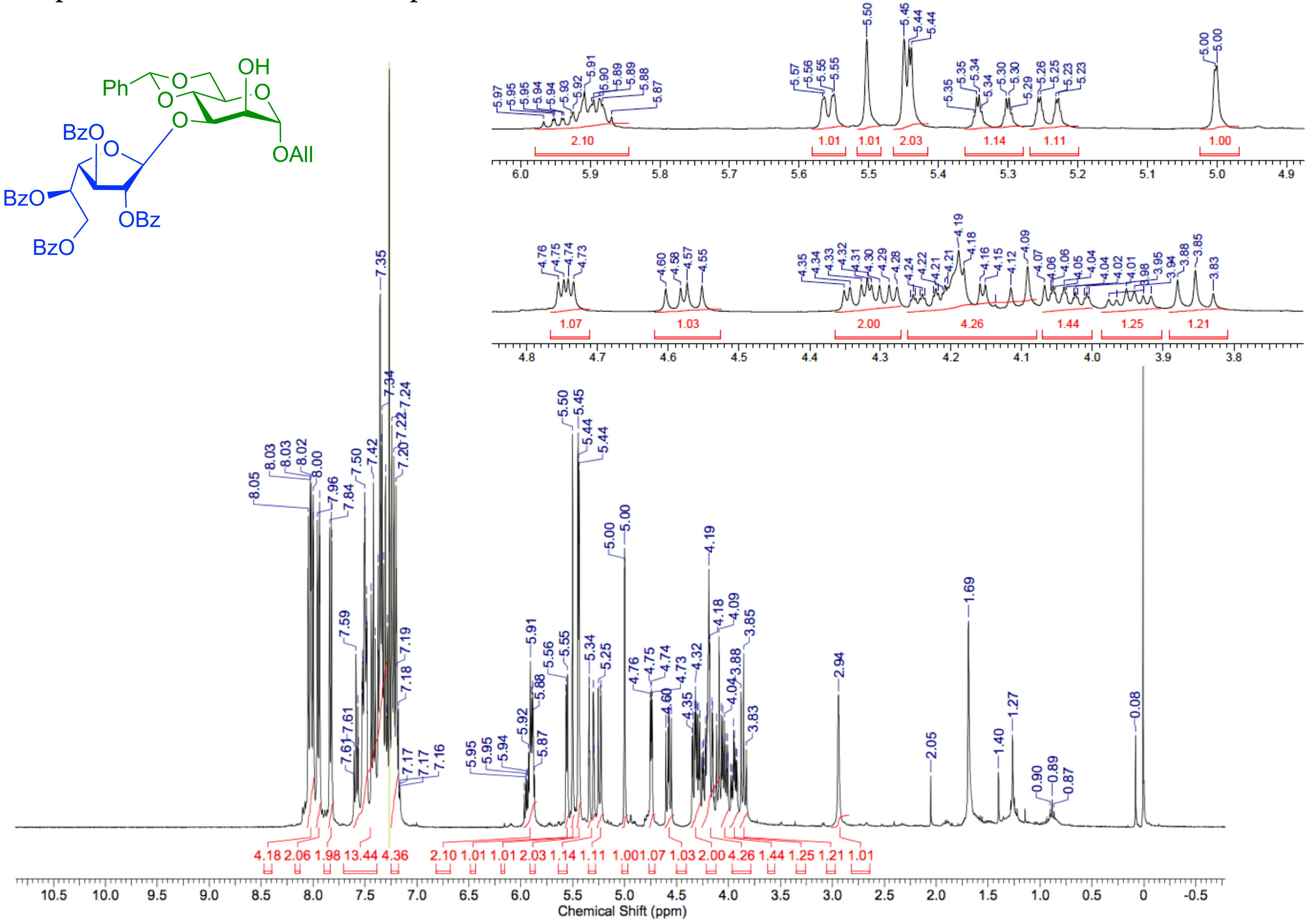

^{13}C NMR, 100 MHz spectrum, $CDCl_3$, compound 3

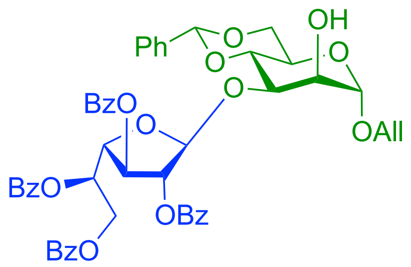

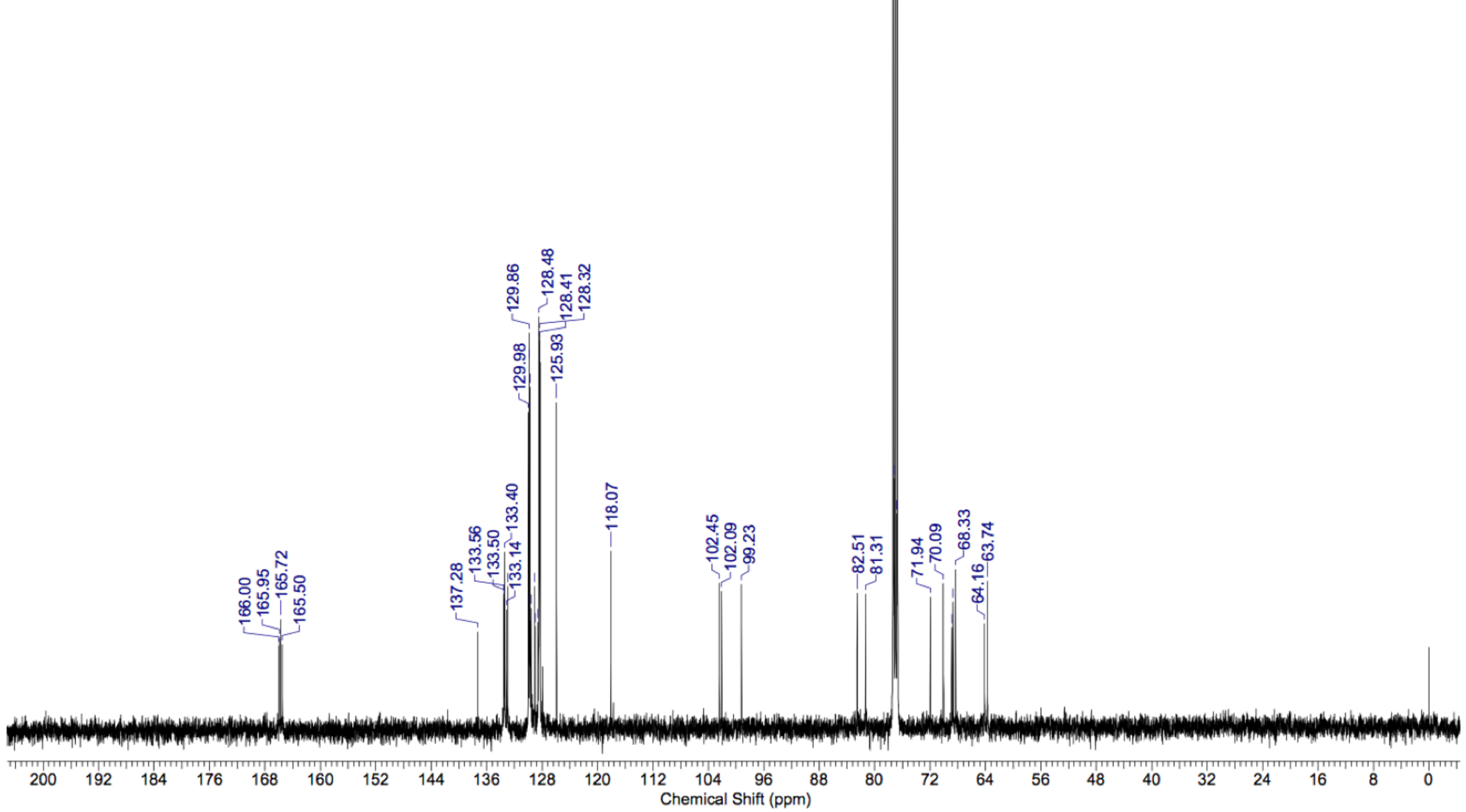

HSQC NMR spectrum, 400 MHz, $CDCl_3$, compound 3

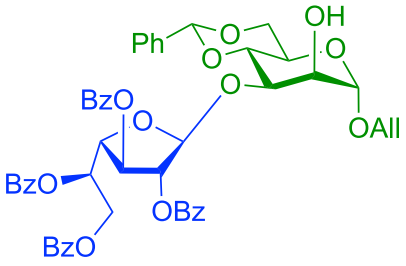

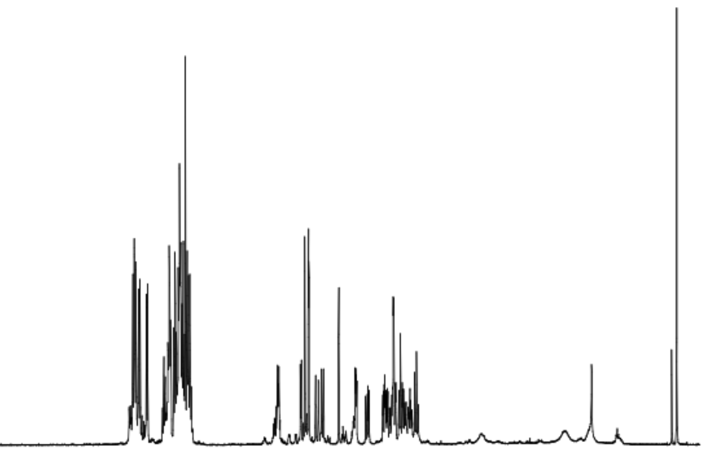

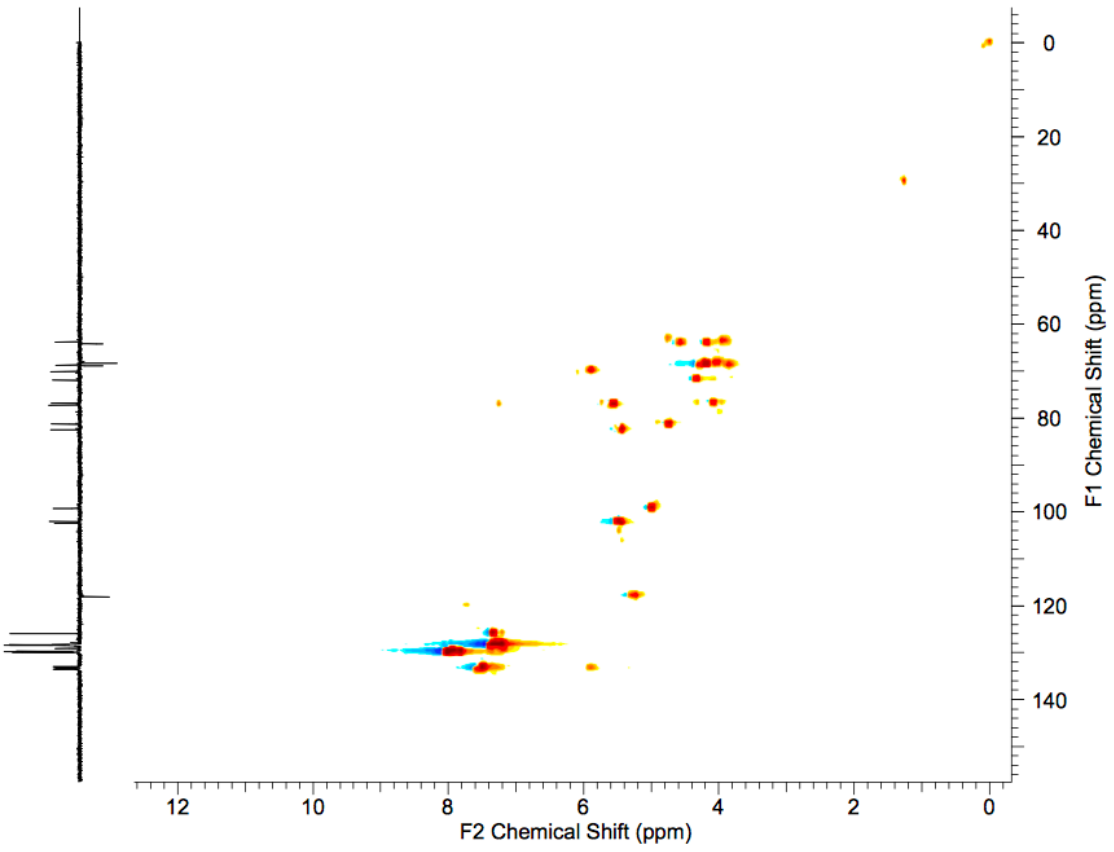

COSY NMR spectrum, 400 MHz, $CDCl_3$, compound 3

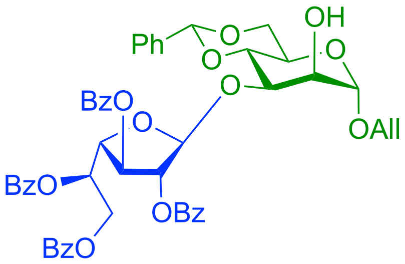

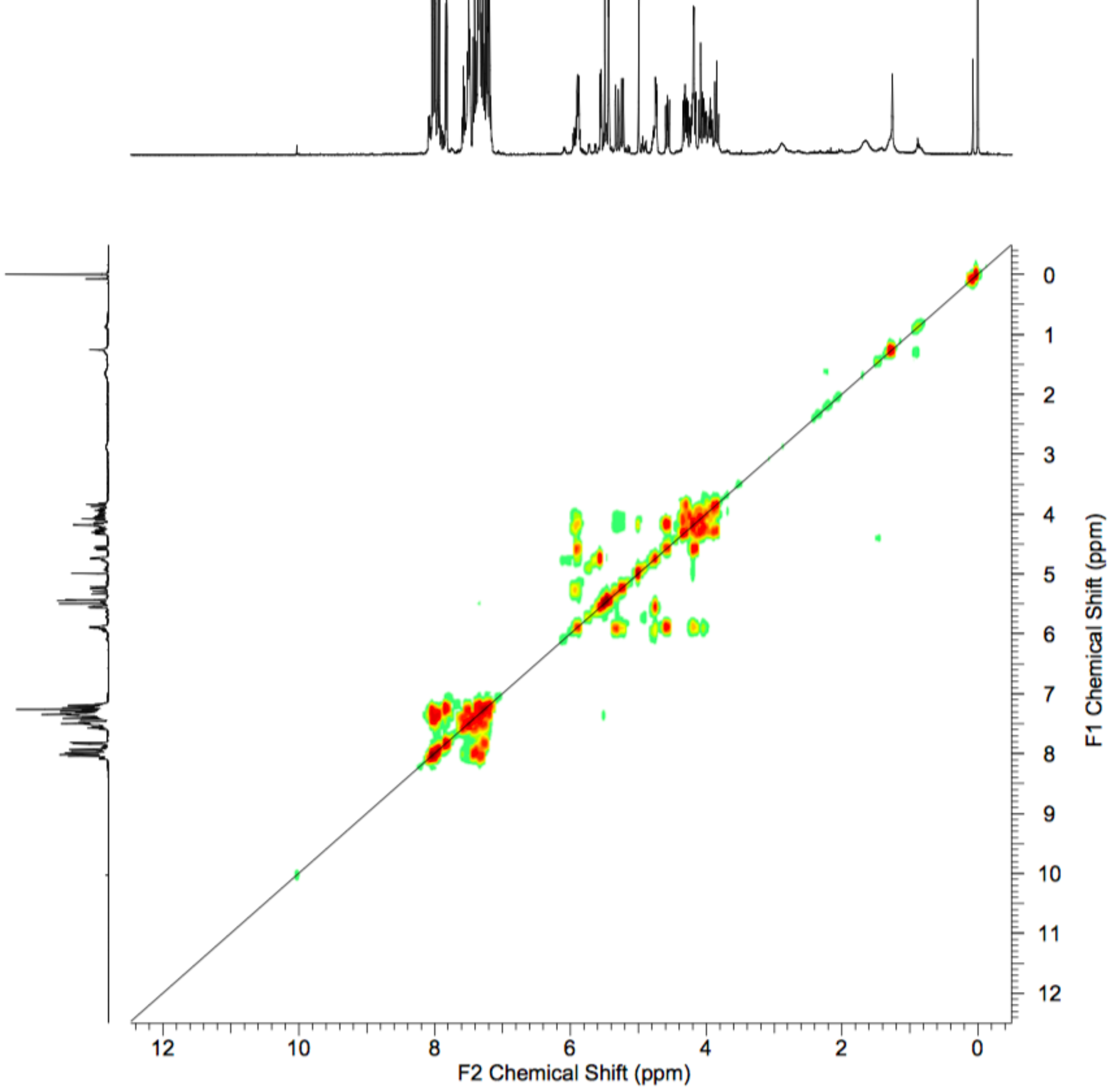

ESI-TOF HR mass spectrum of compound 3

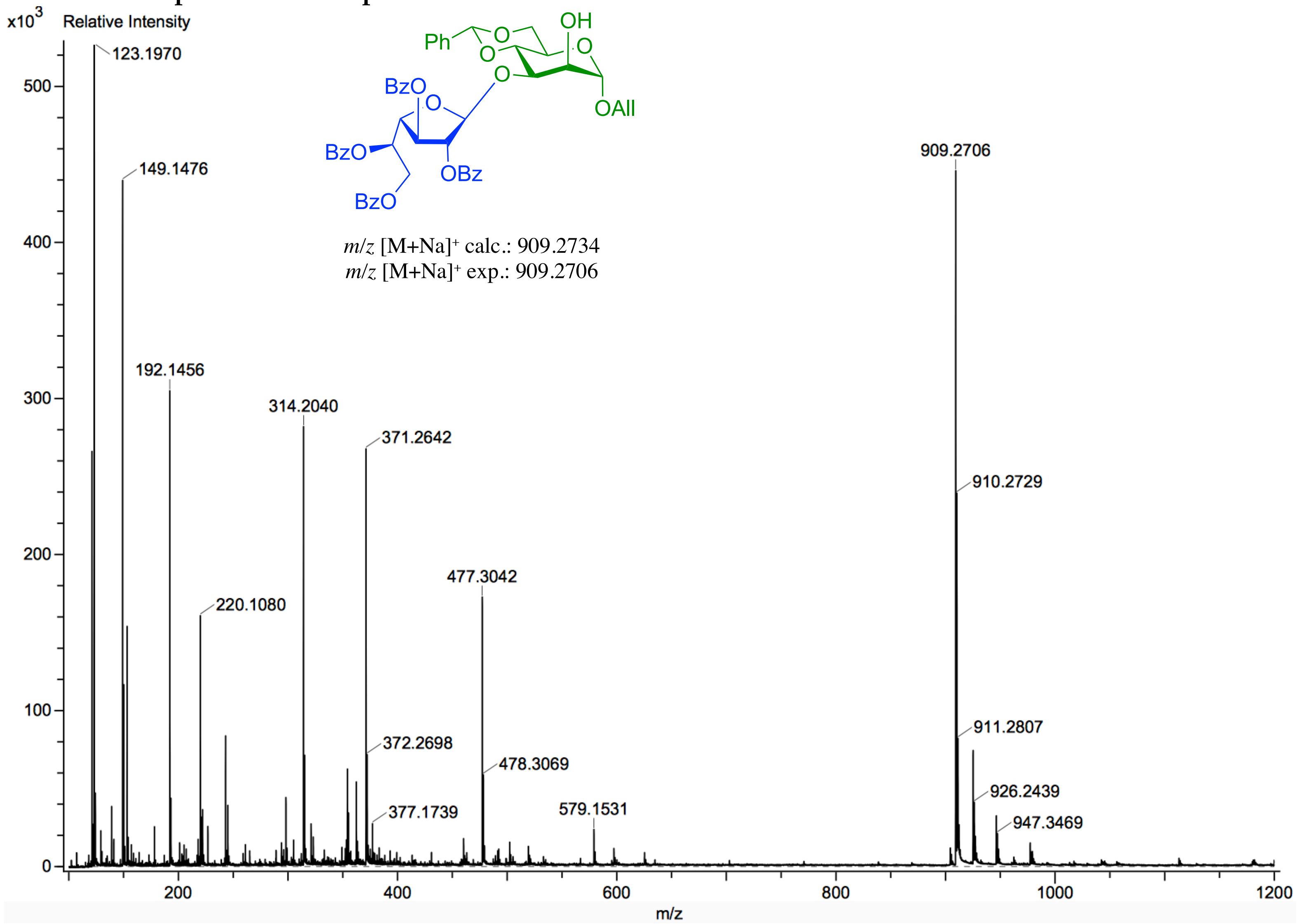

^{1}H NMR spectrum, 400 MHz, $CDCl_3$, compound S1

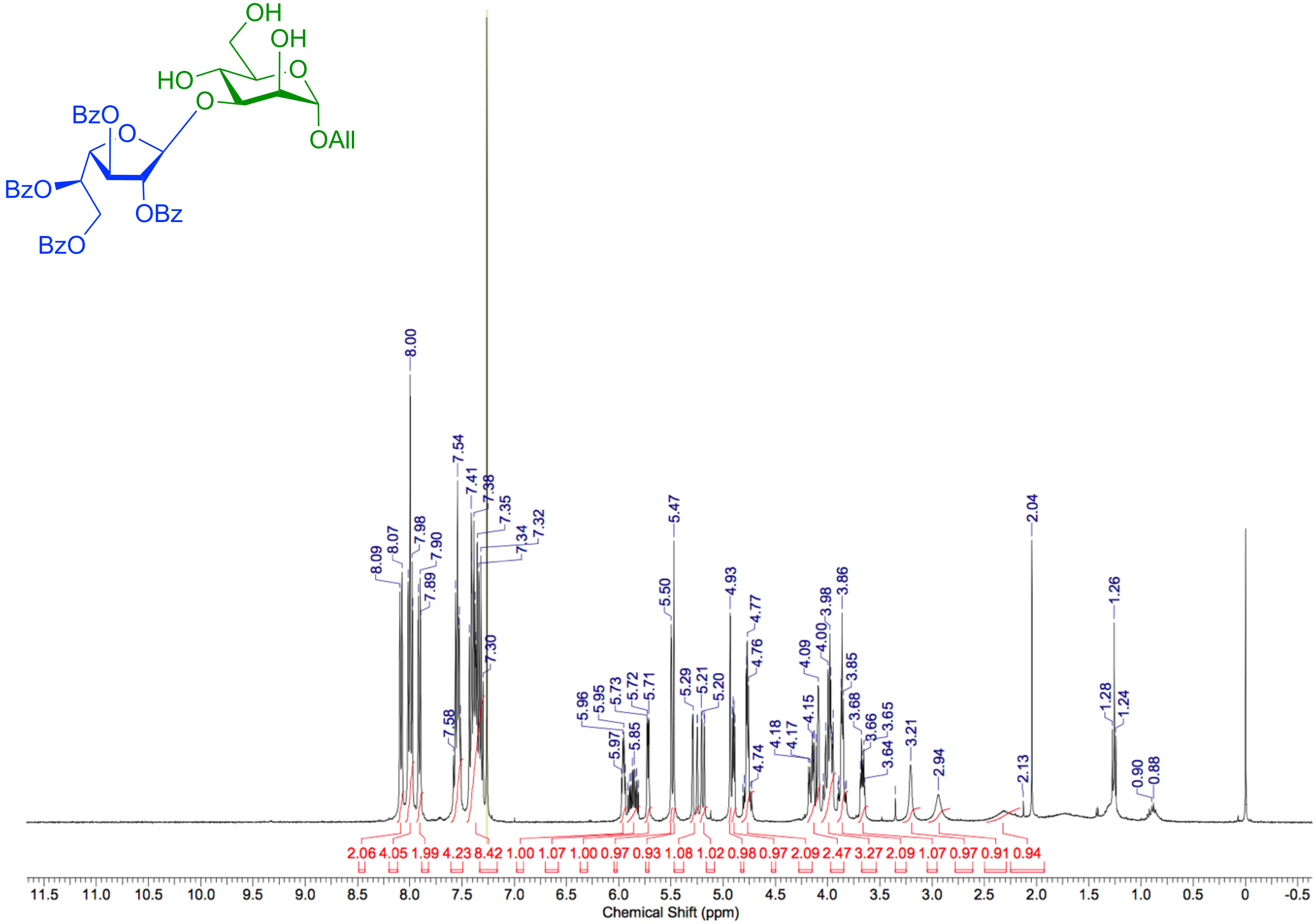

^{13}C NMR spectrum, 100 MHz, $CDCl_3$, compound S1

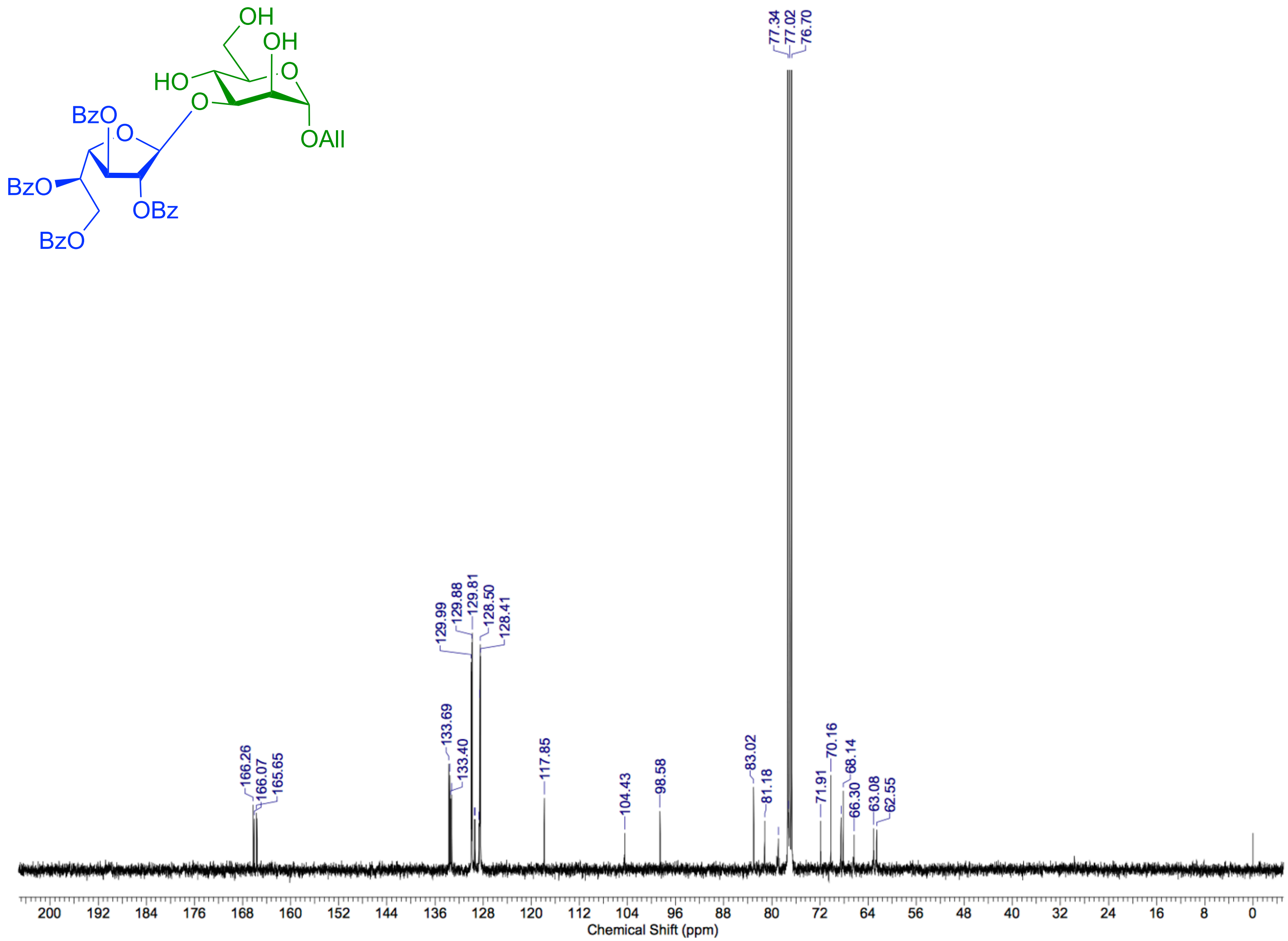

ESI-TOF HR mass spectrum of compound S1

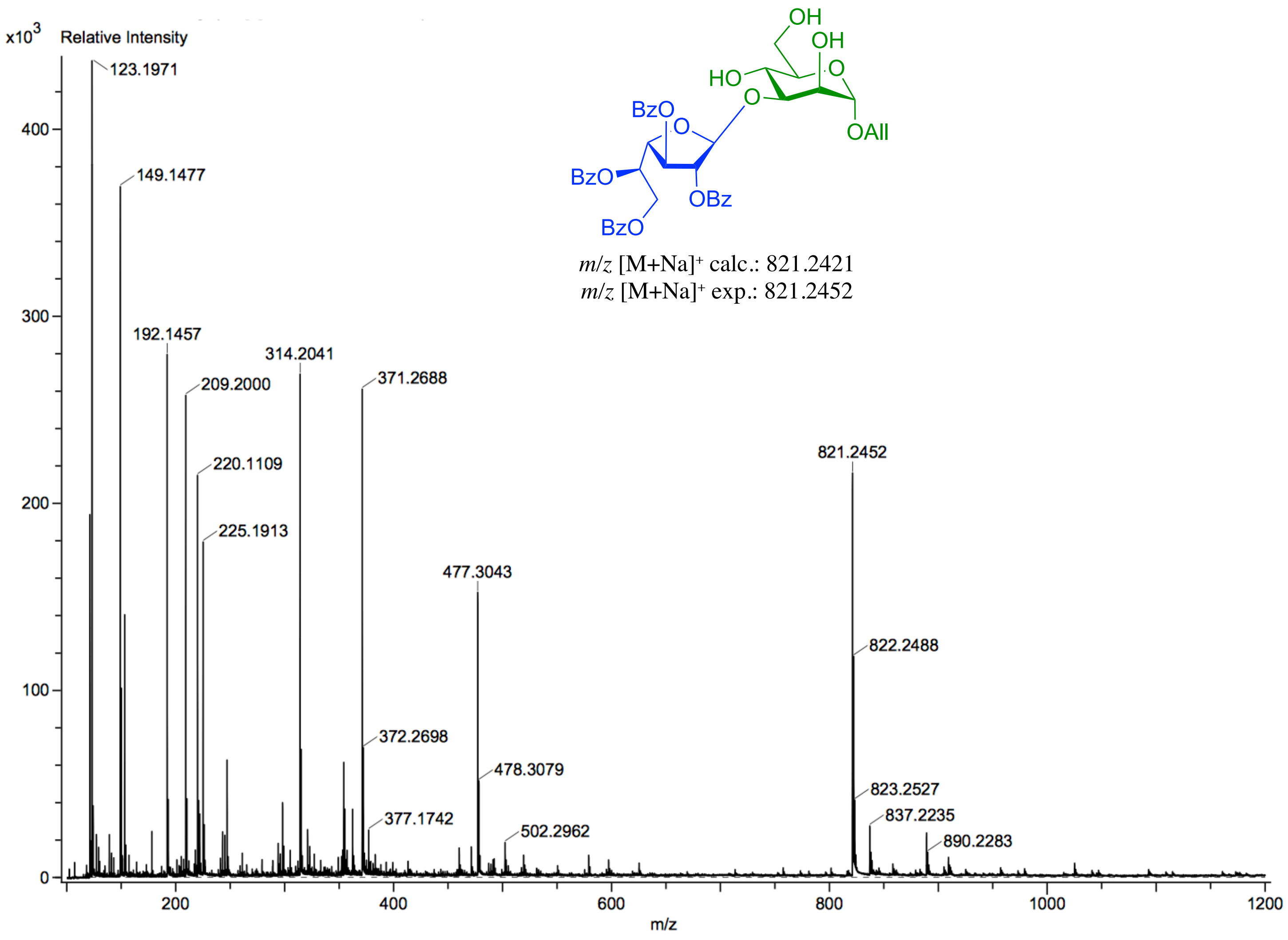

^{1}H NMR spectrum, 400 MHz, $CDCl_3$, compound 4

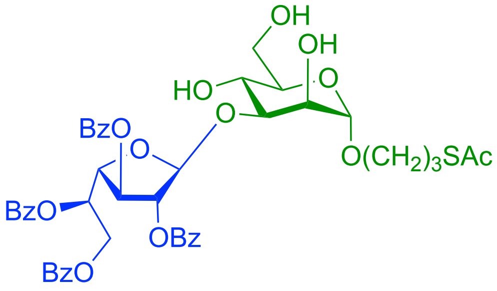

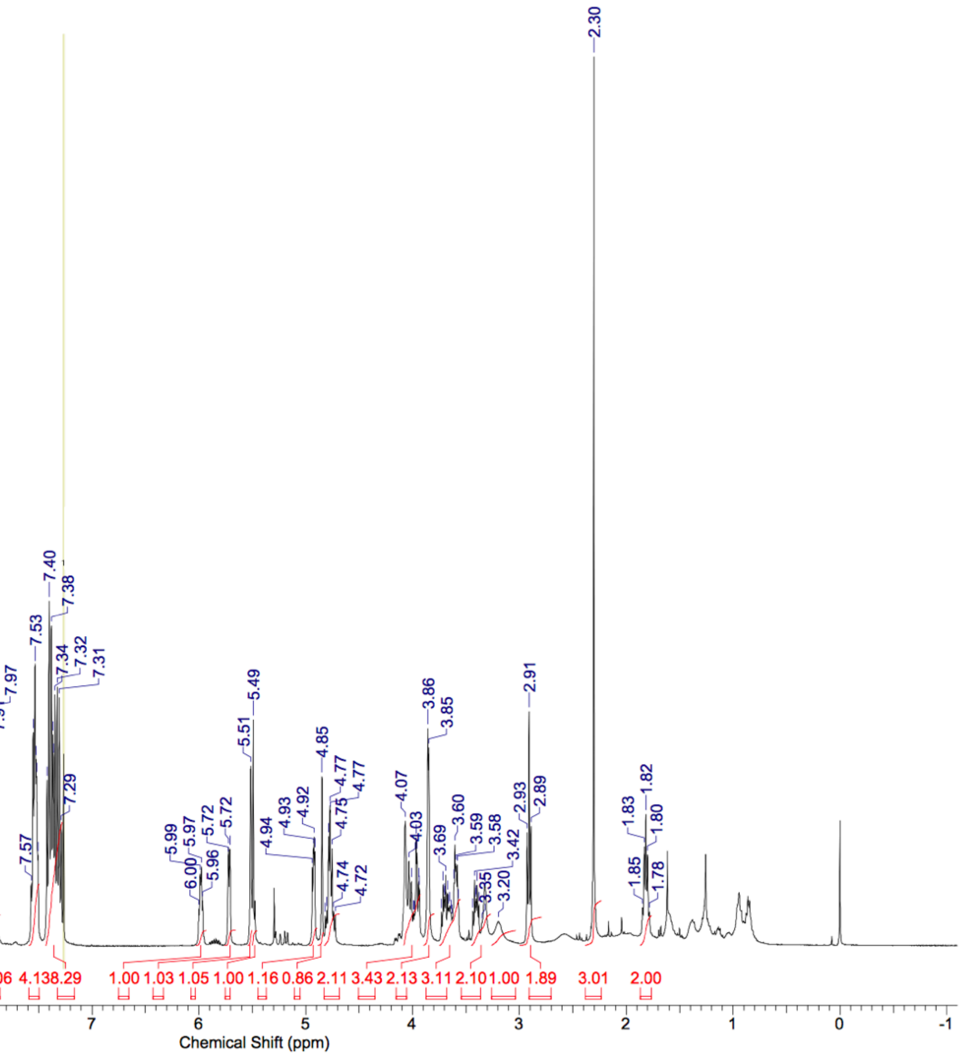

^{13}C NMR spectrum, 100 MHz, $CDCl_3$, compound 4

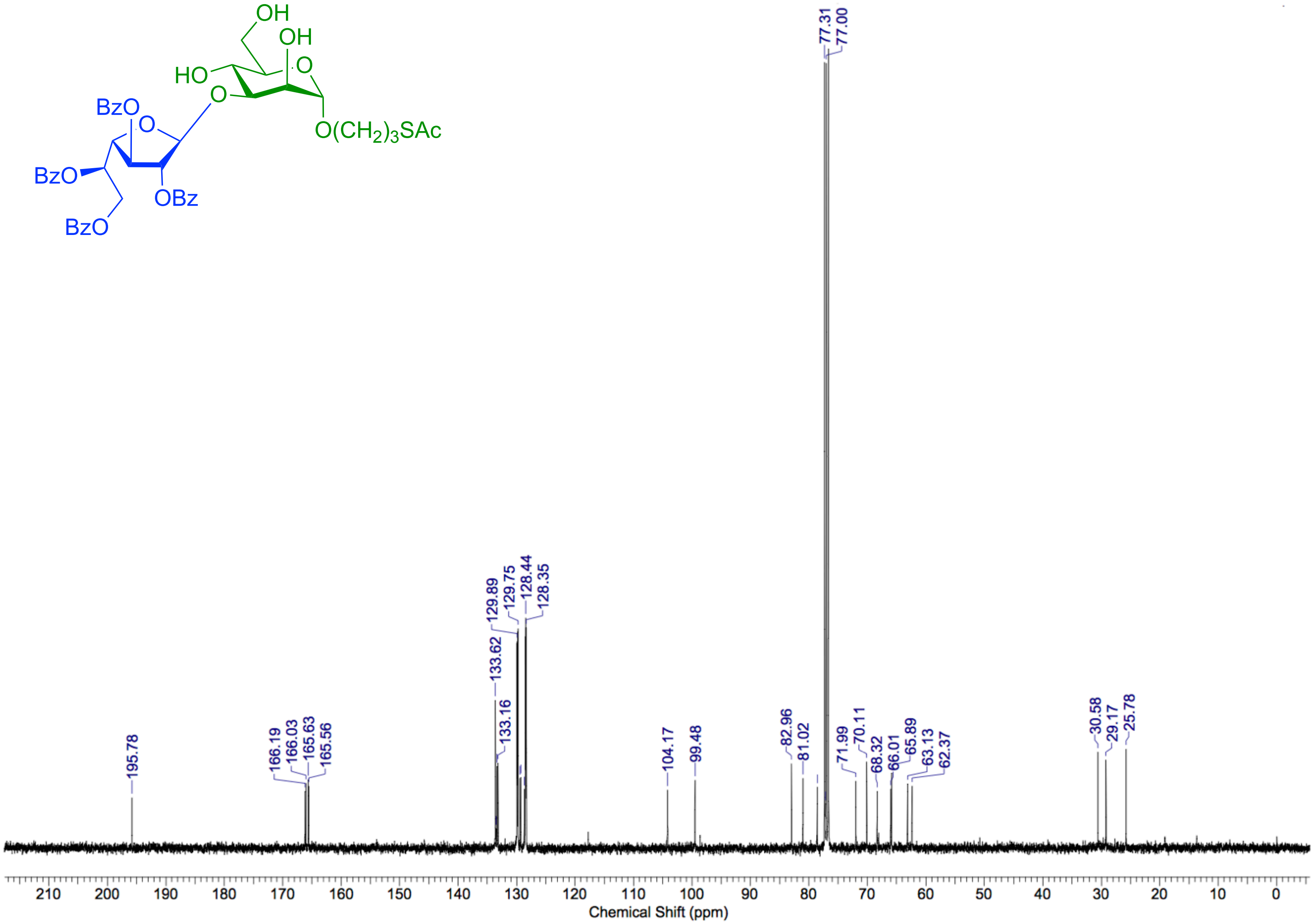

ESI-TOF HR mass spectrum of compound 4

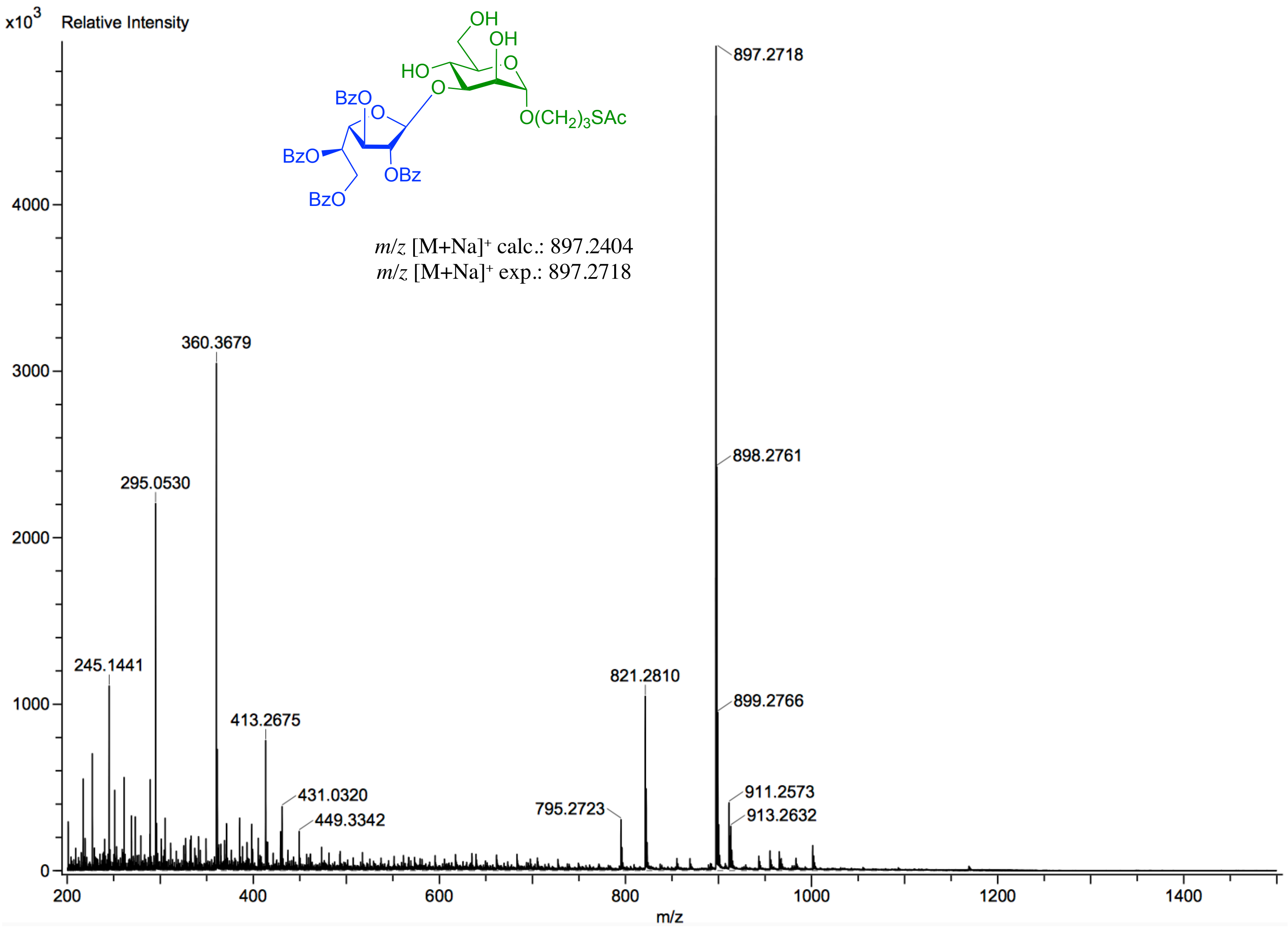

^{1}H NMR spectrum, 400 MHz, D_2O, compound G29$_{SH}$ [and (G29$_S$)$_2$]

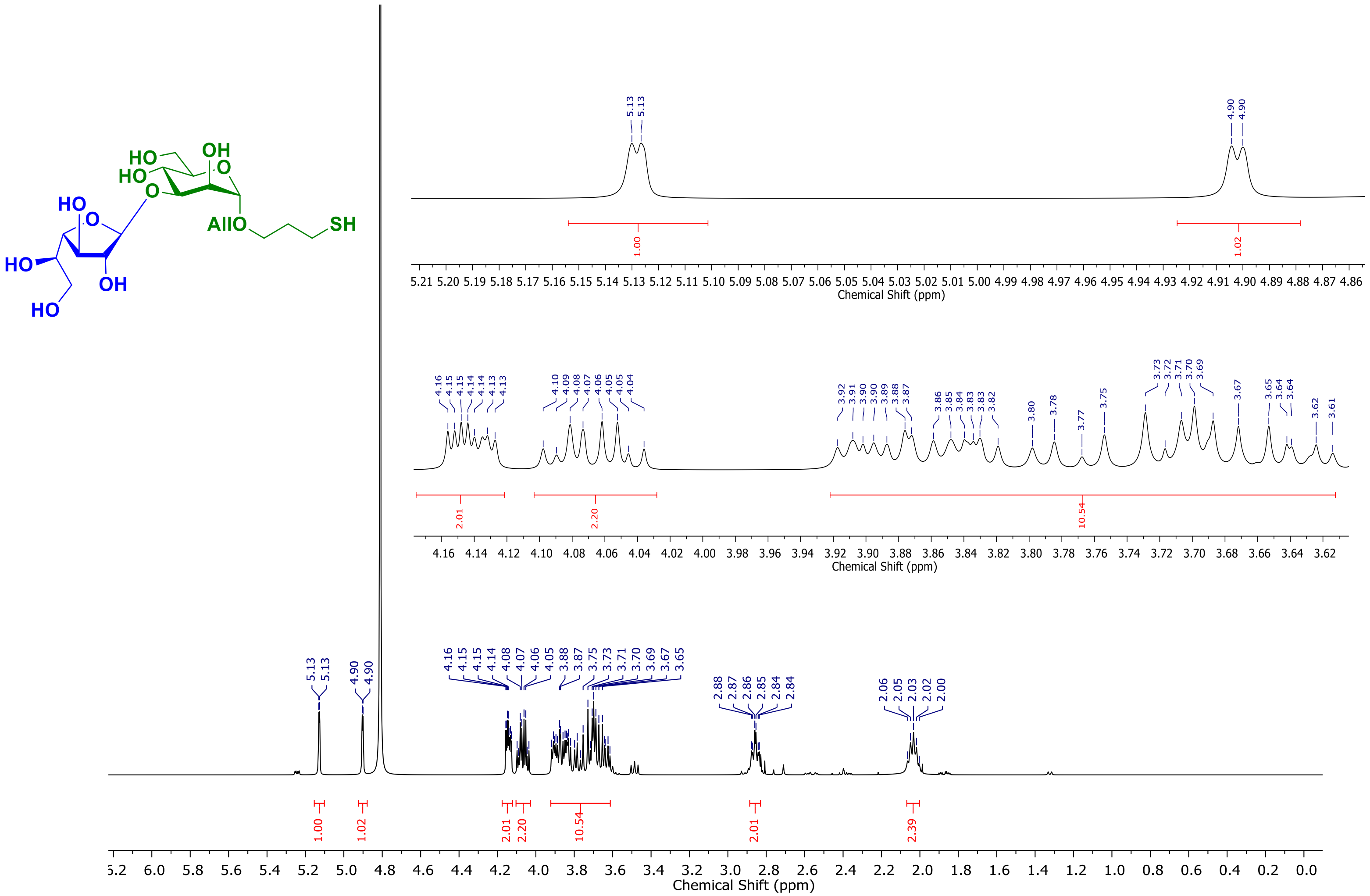

^{13}C NMR spectrum, 100 MHz, D_2O, compound G29$_{SH}$ [and (G29$_S$)$_2$]

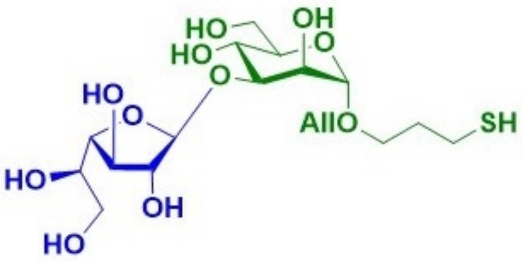

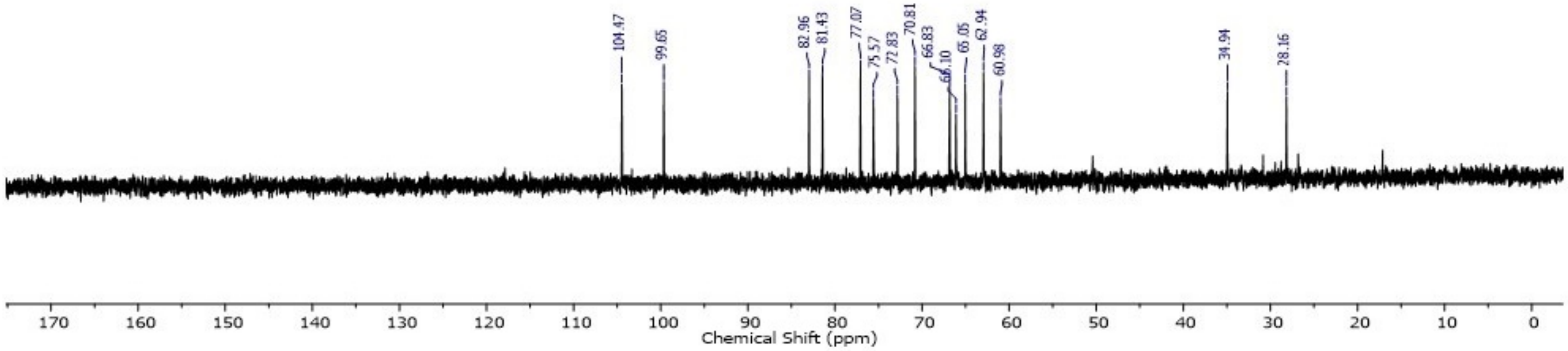

ESI-TOF HR mass spectrum of compounds G29SH and (G29S)2

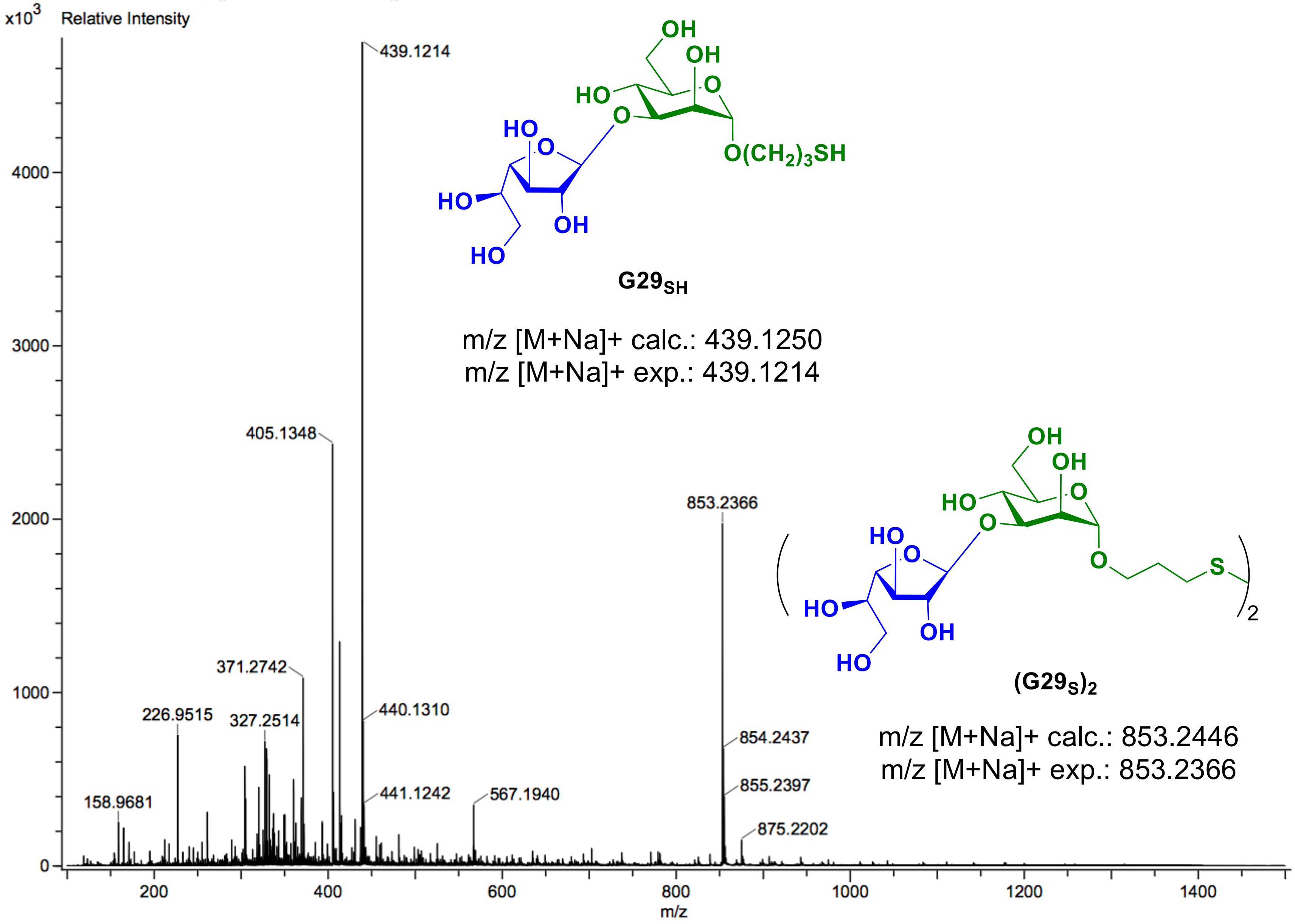

¹H NMR spectrum, 400 MHz, $CDCl_3$, compound S2

(Residual ethyl acetate is present.)

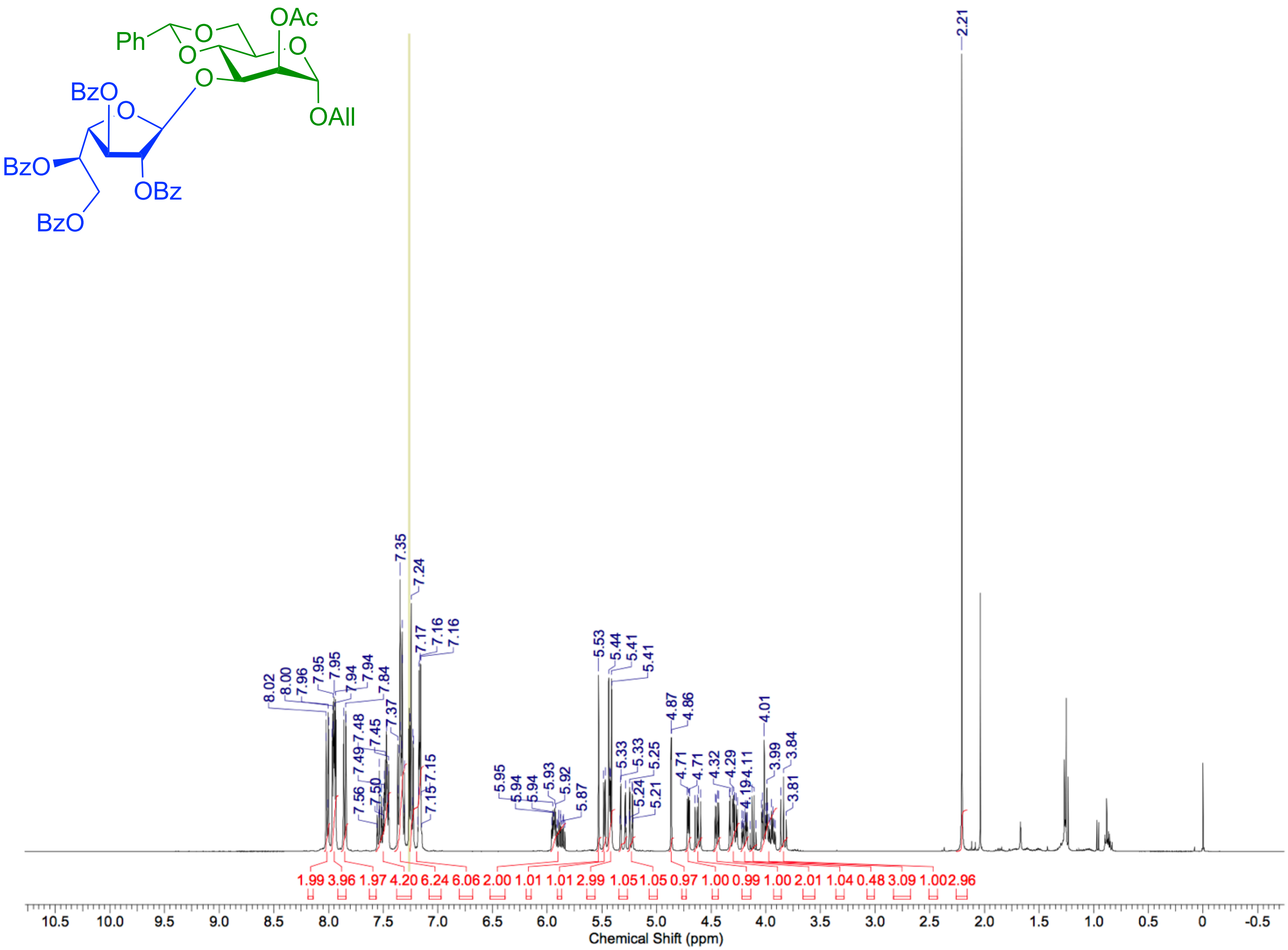

^{13}C NMR spectrum, 100 MHz, $CDCl_3$, compound S2

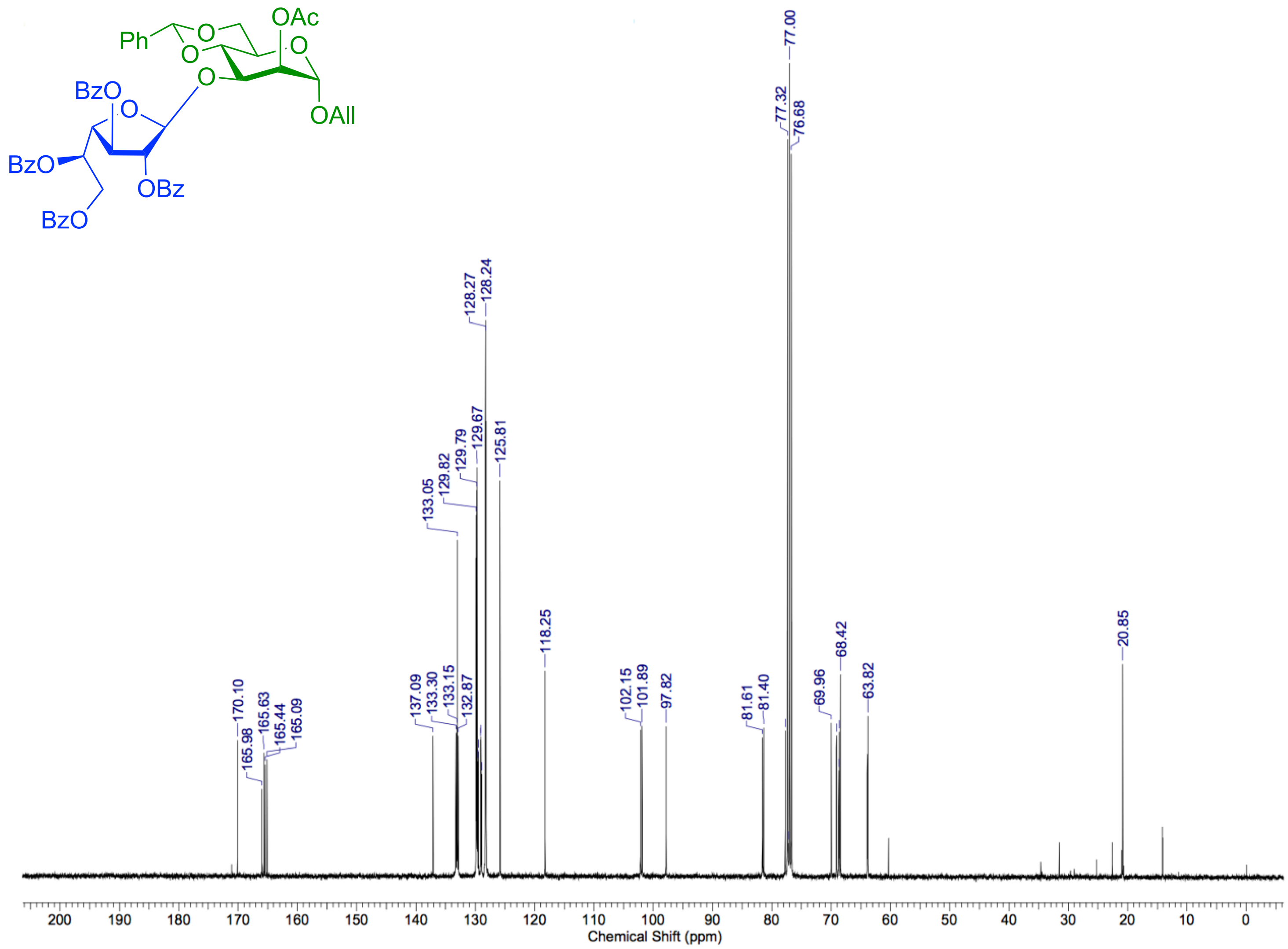

ESI-TOF HR mass spectrum of compound S2

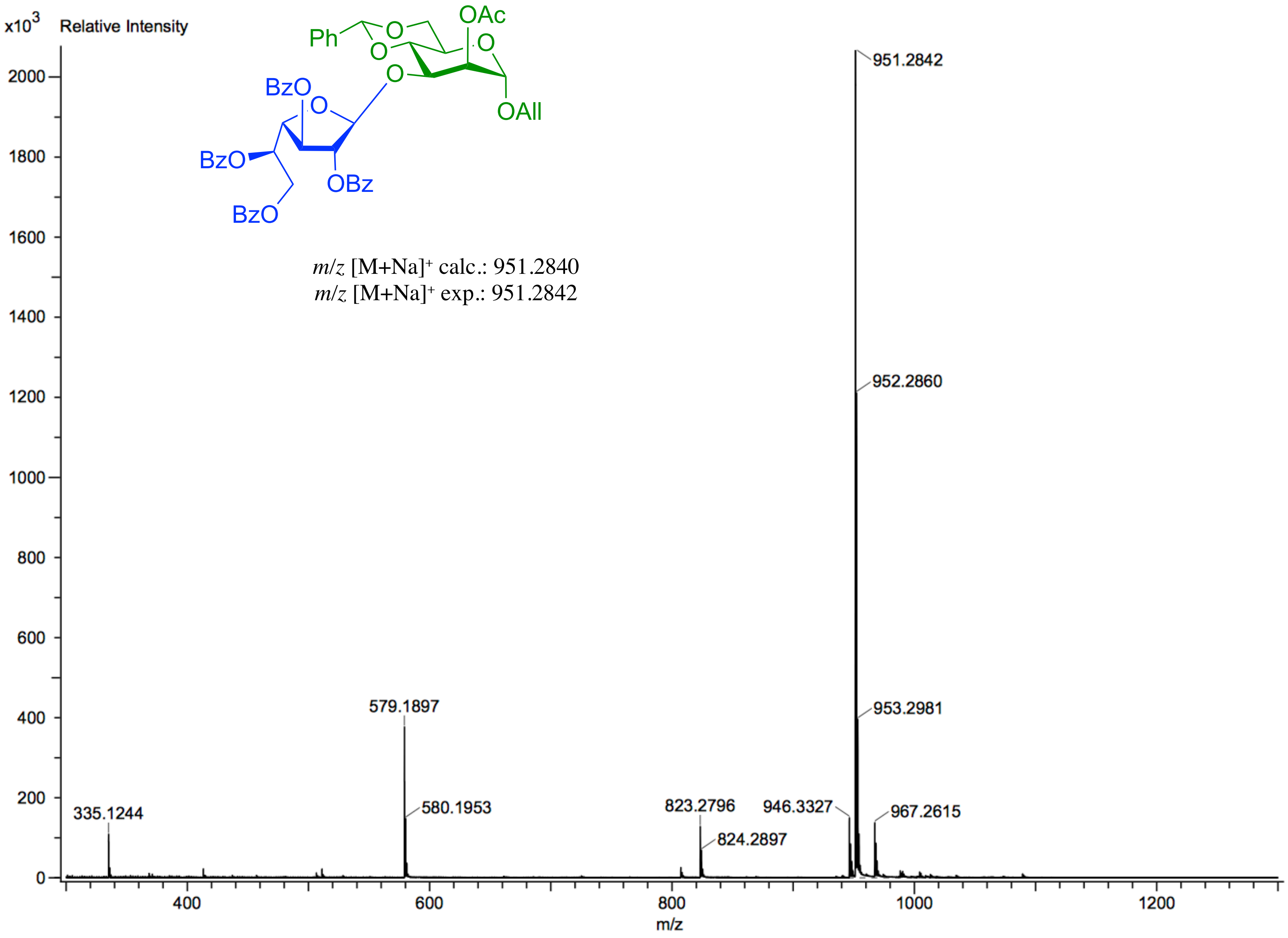

^{1}H NMR spectrum, 400 MHz, $CDCl_3$, compound S3

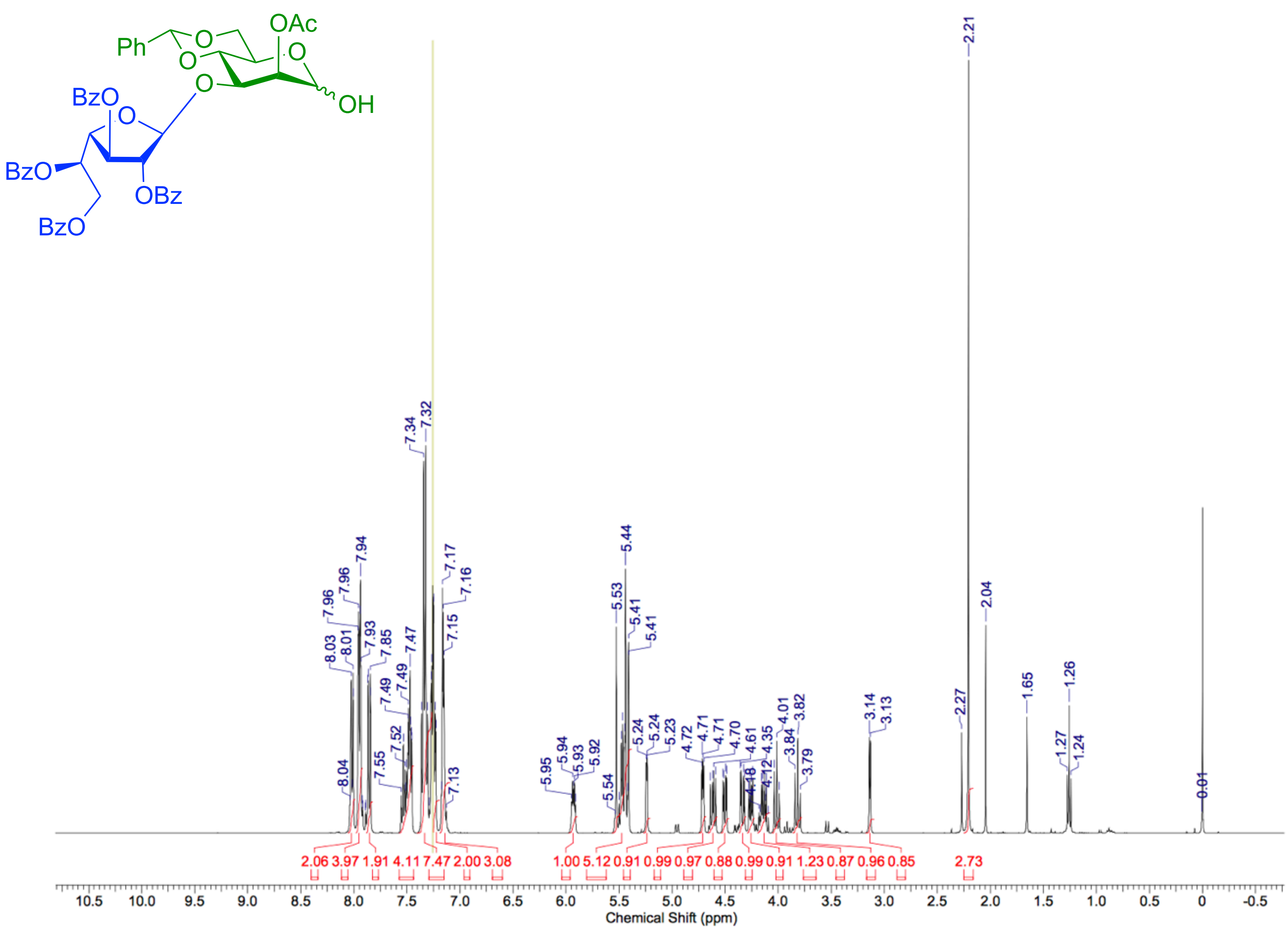

^{13}C NMR spectrum, 100 MHz, $CDCl_3$, compound S3

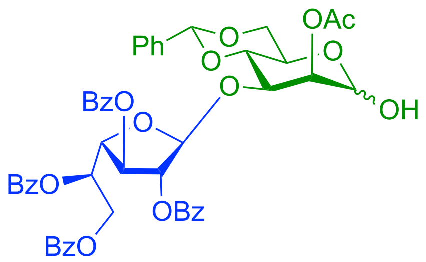

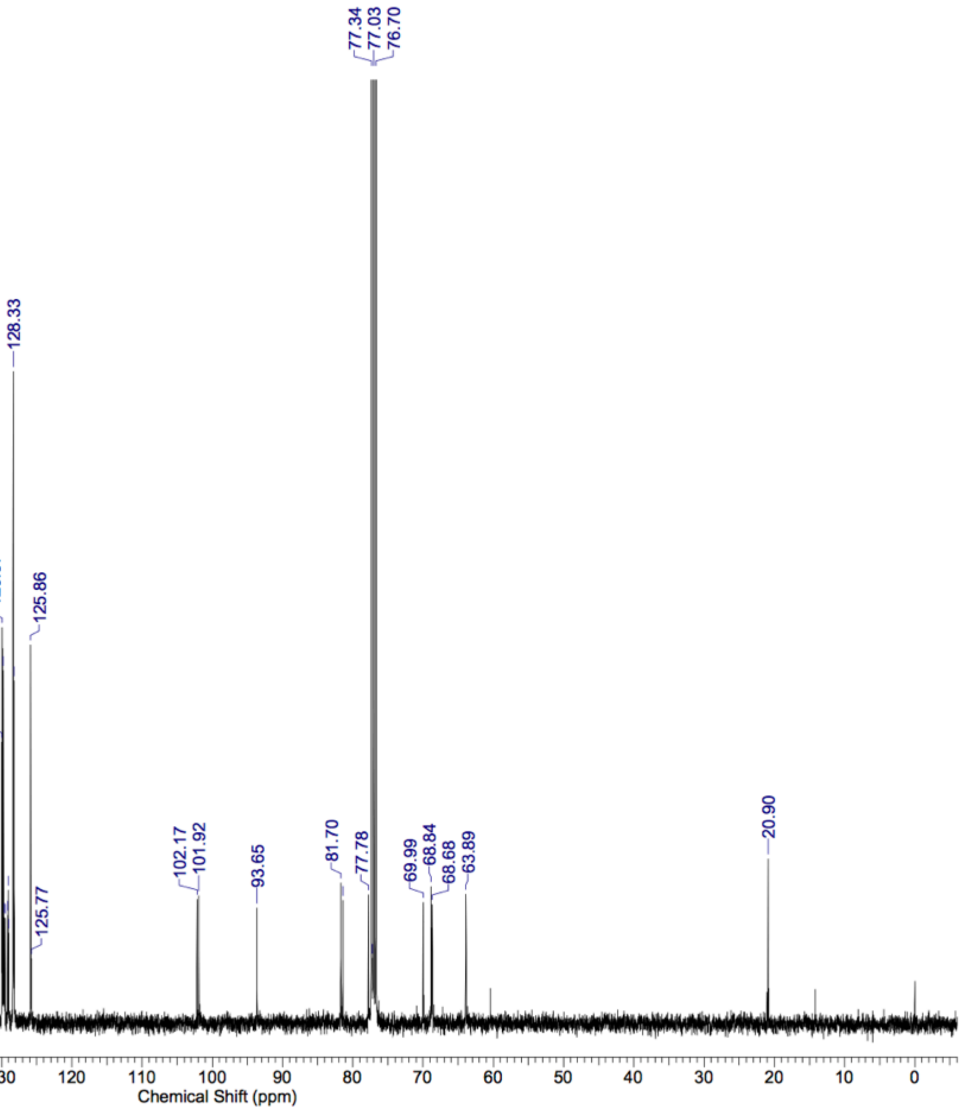

ESI-TOF HR mass spectrum of compound S3

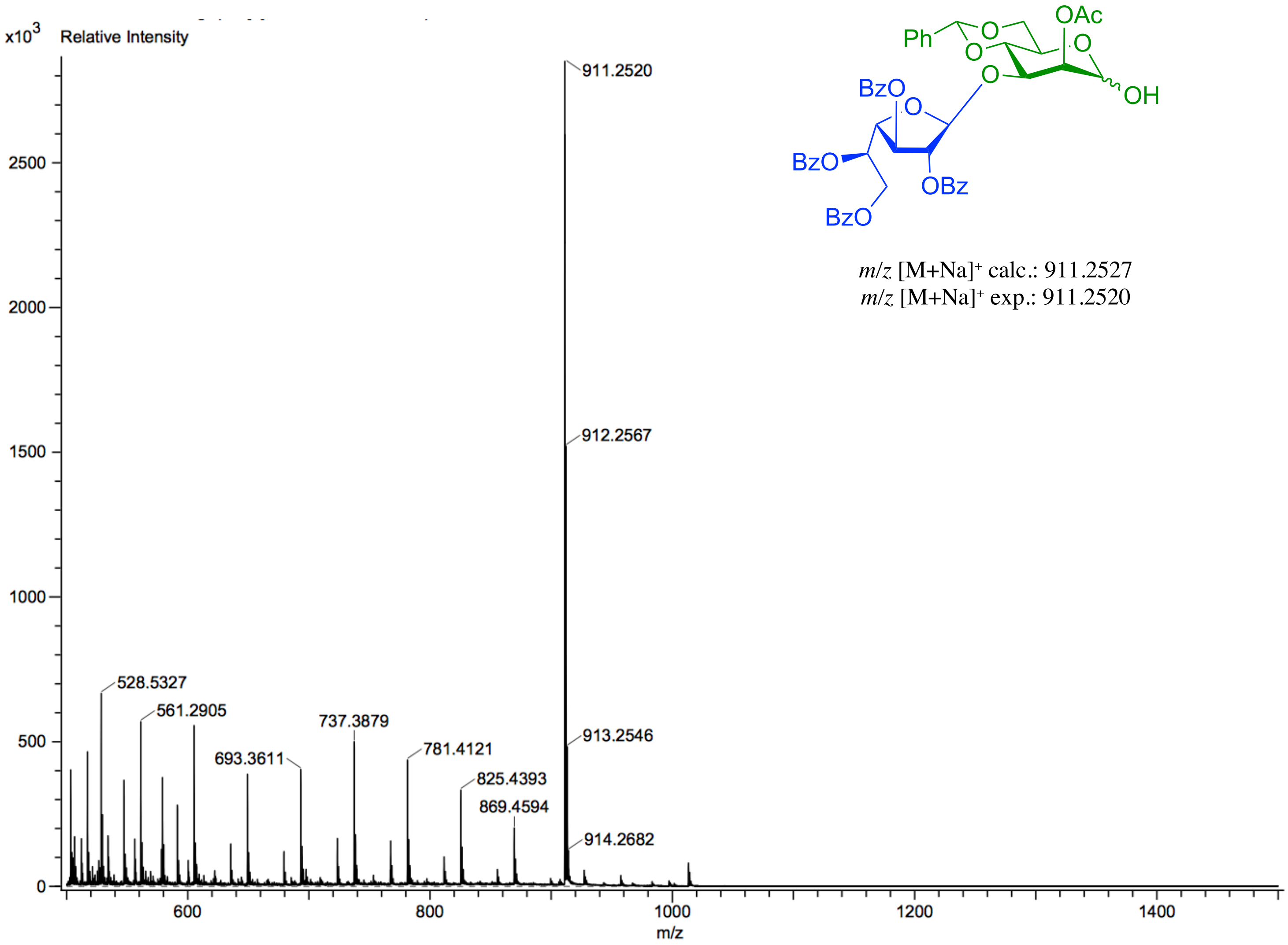

m/z [M+Na]+ calc.: 911.2527
m/z [M+Na]+ exp.: 911.2520

¹H NMR spectrum, 400 MHz, CDCl₃, compound 5

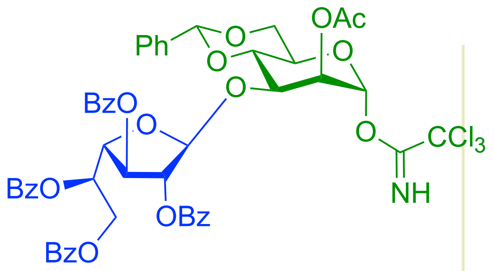

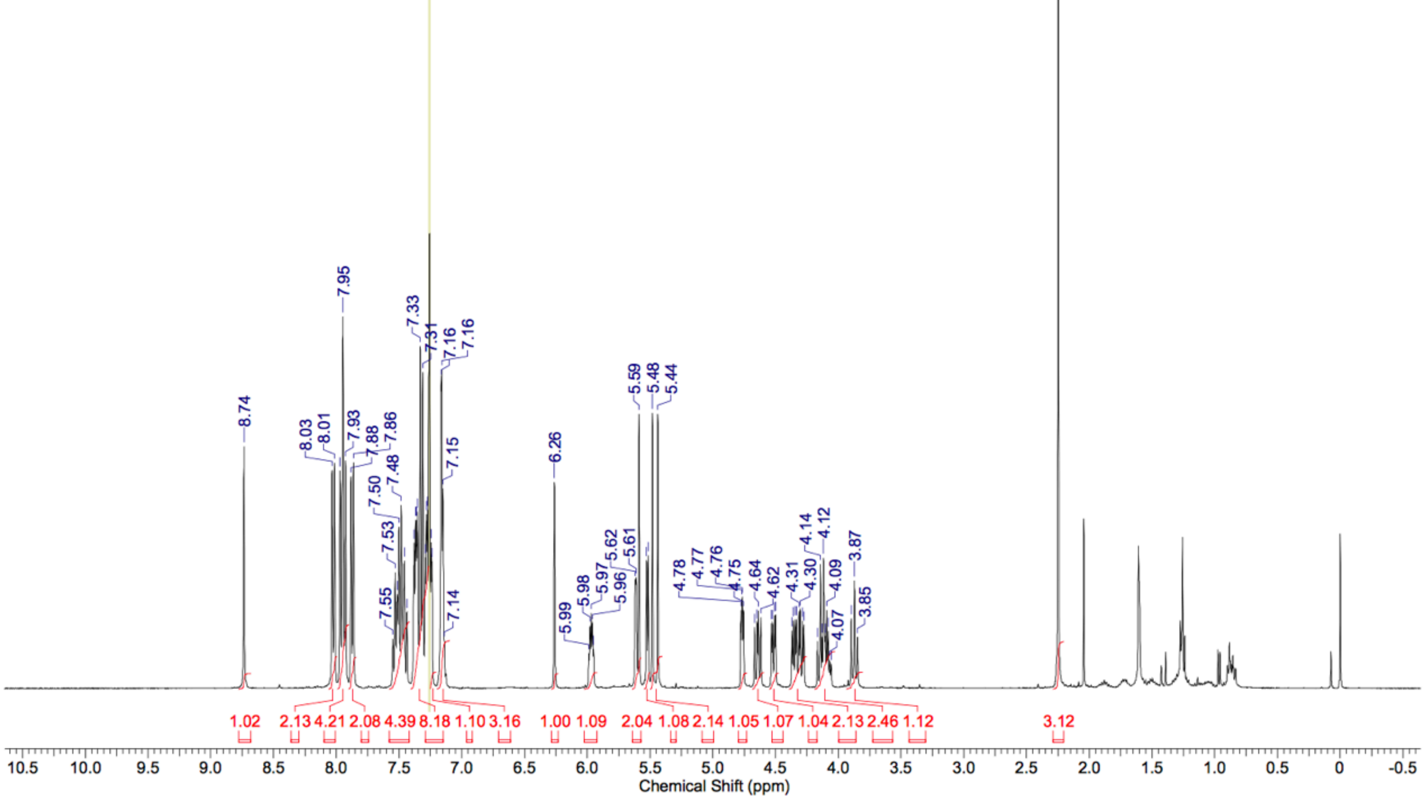

^{13}C NMR spectrum, 100 MHz, $CDCl_3$, compound 5

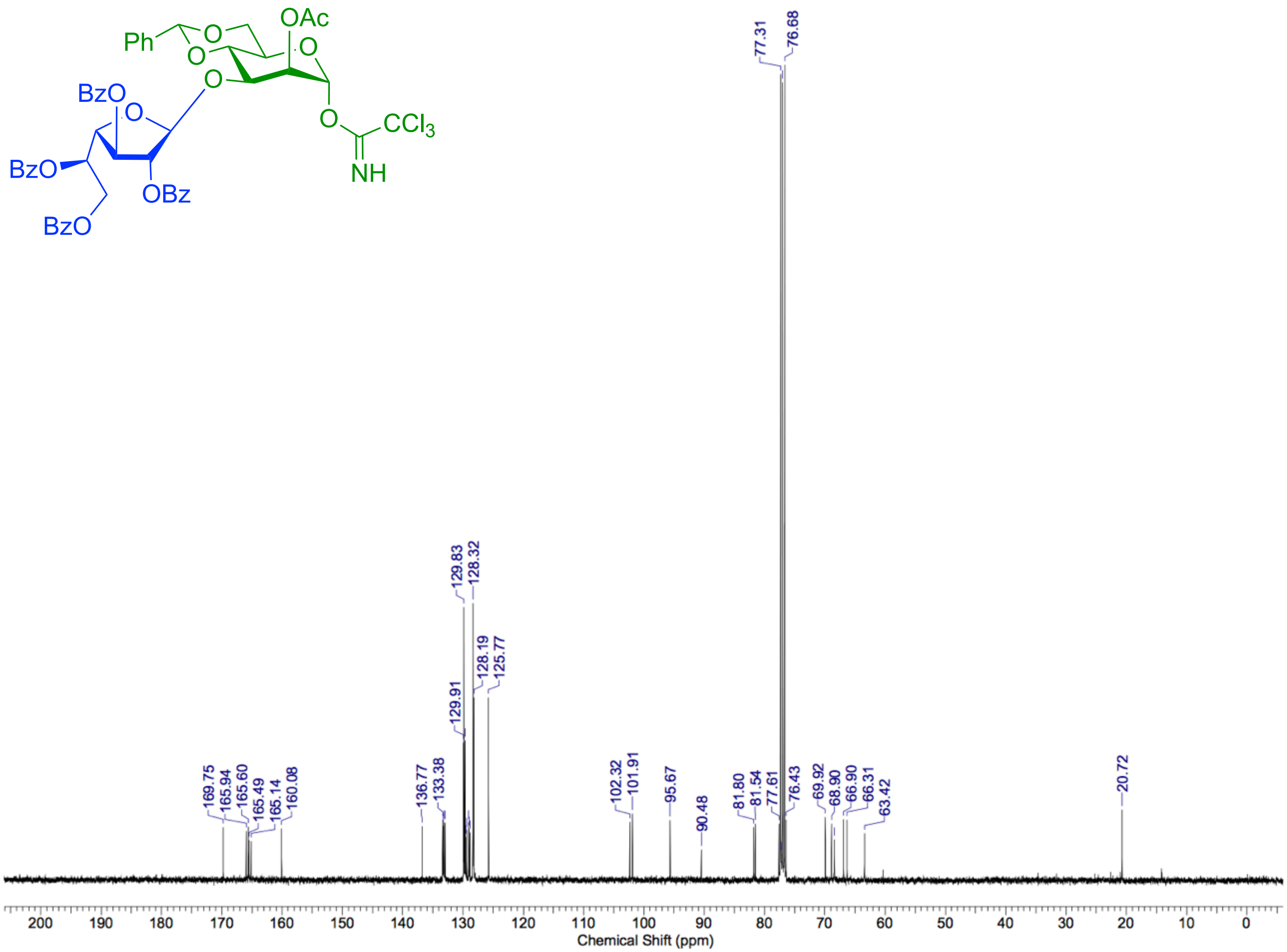

ESI-TOF HR mass spectrum of compound 5

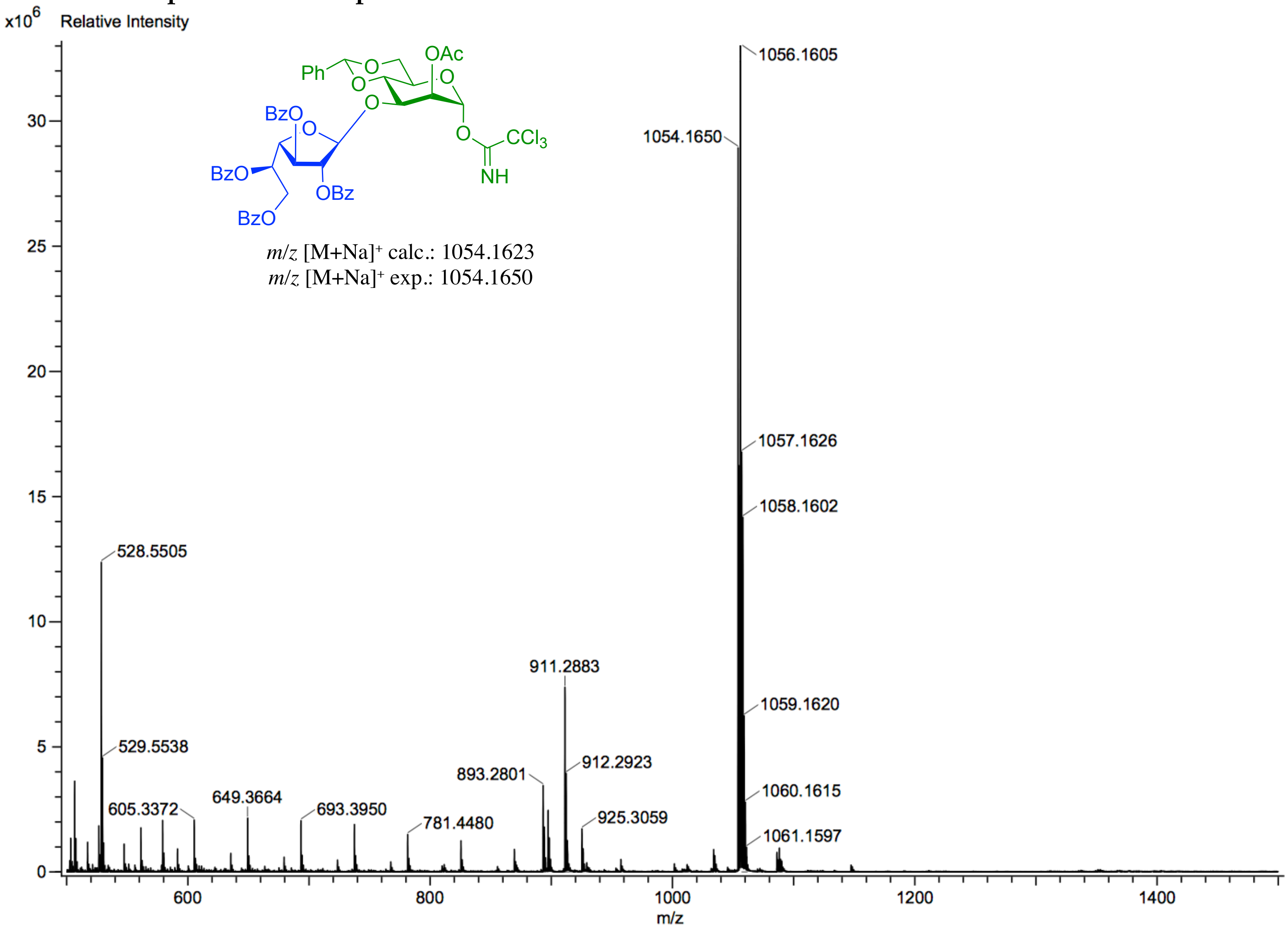

^{1}H NMR spectrum, 400 MHz, $CDCl_3$, compound 6

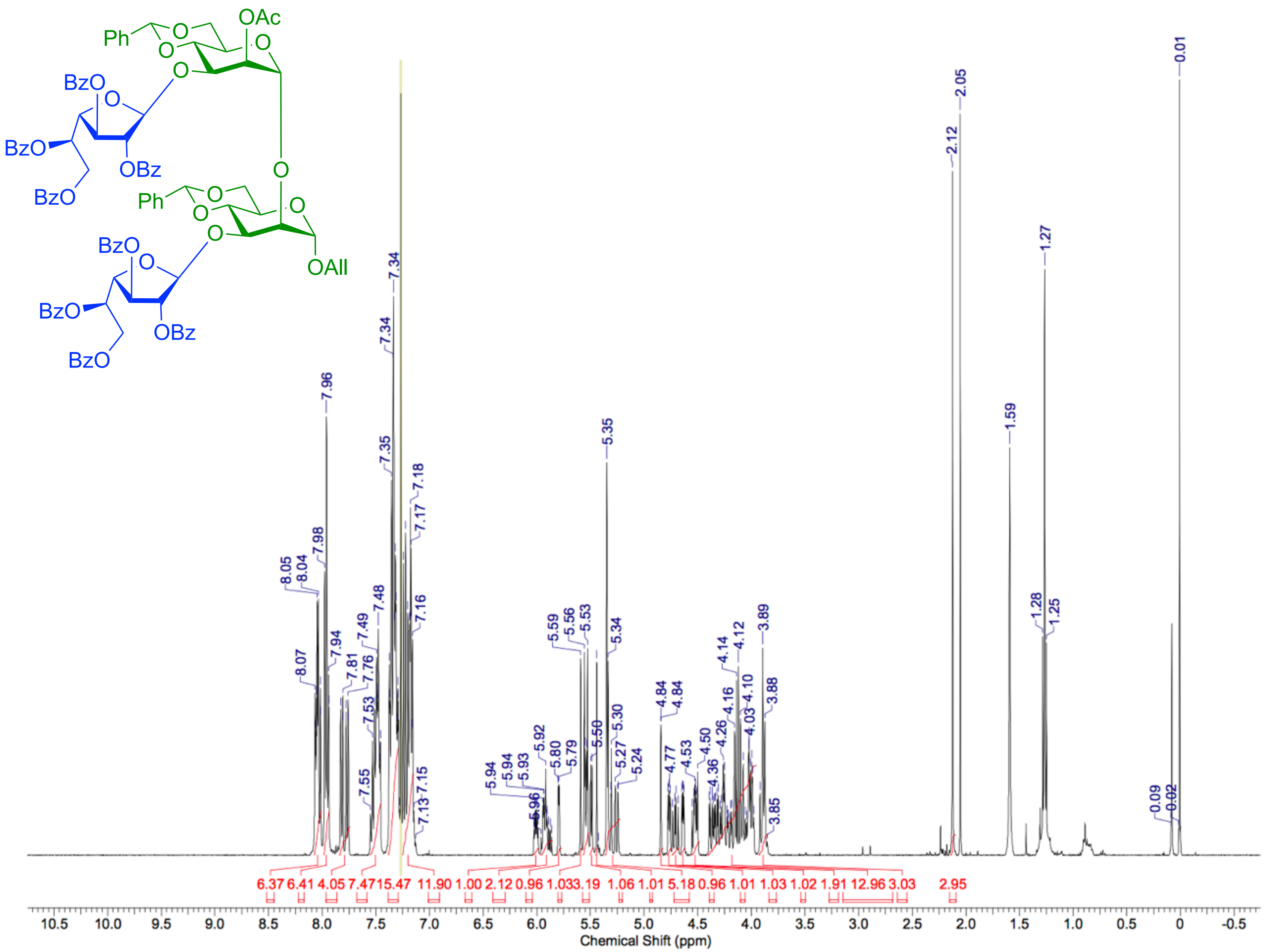

^{13}C NMR spectrum, 100 MHz, CDCl3, compound 6

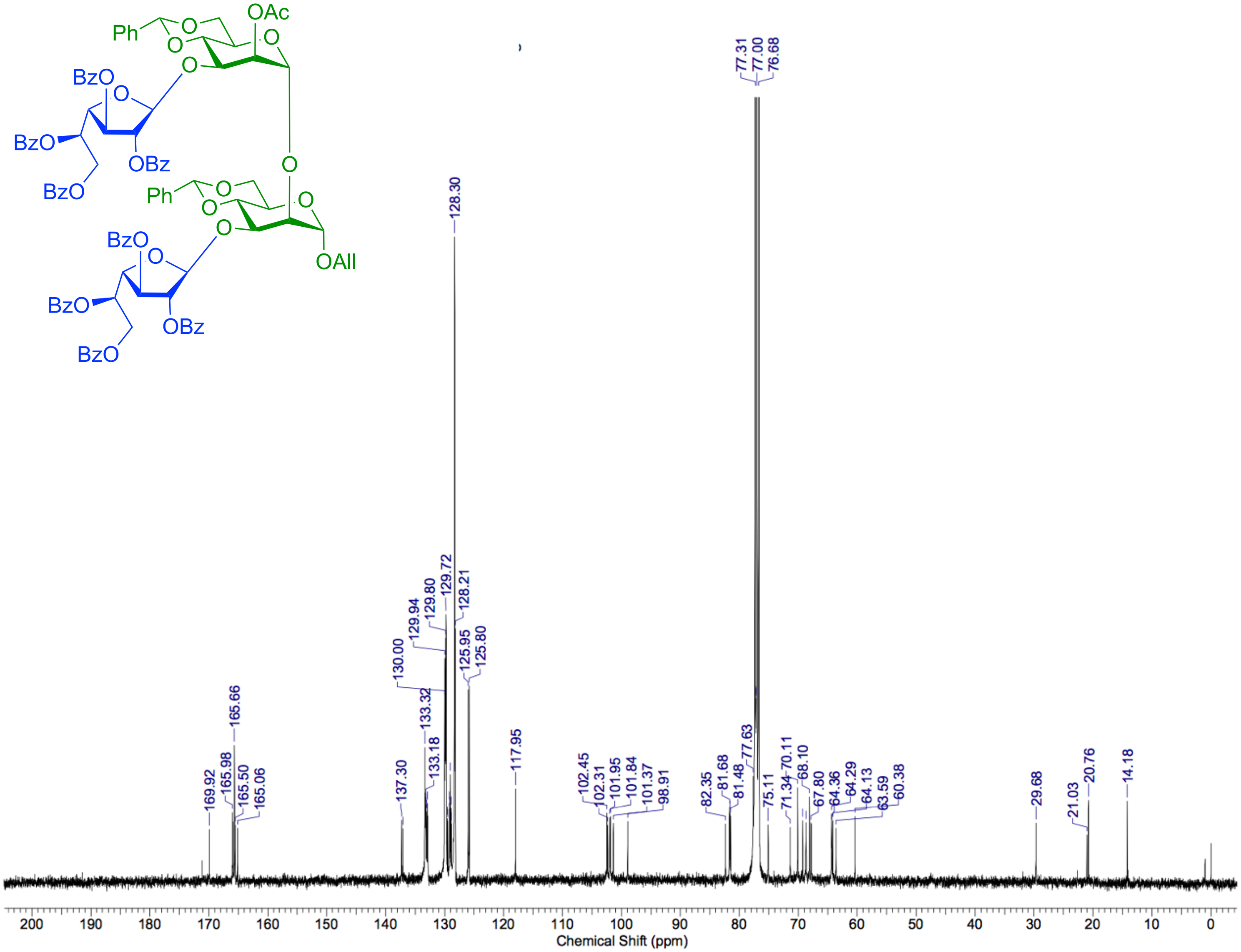

ESI-TOF HR mass spectrum of compound 6

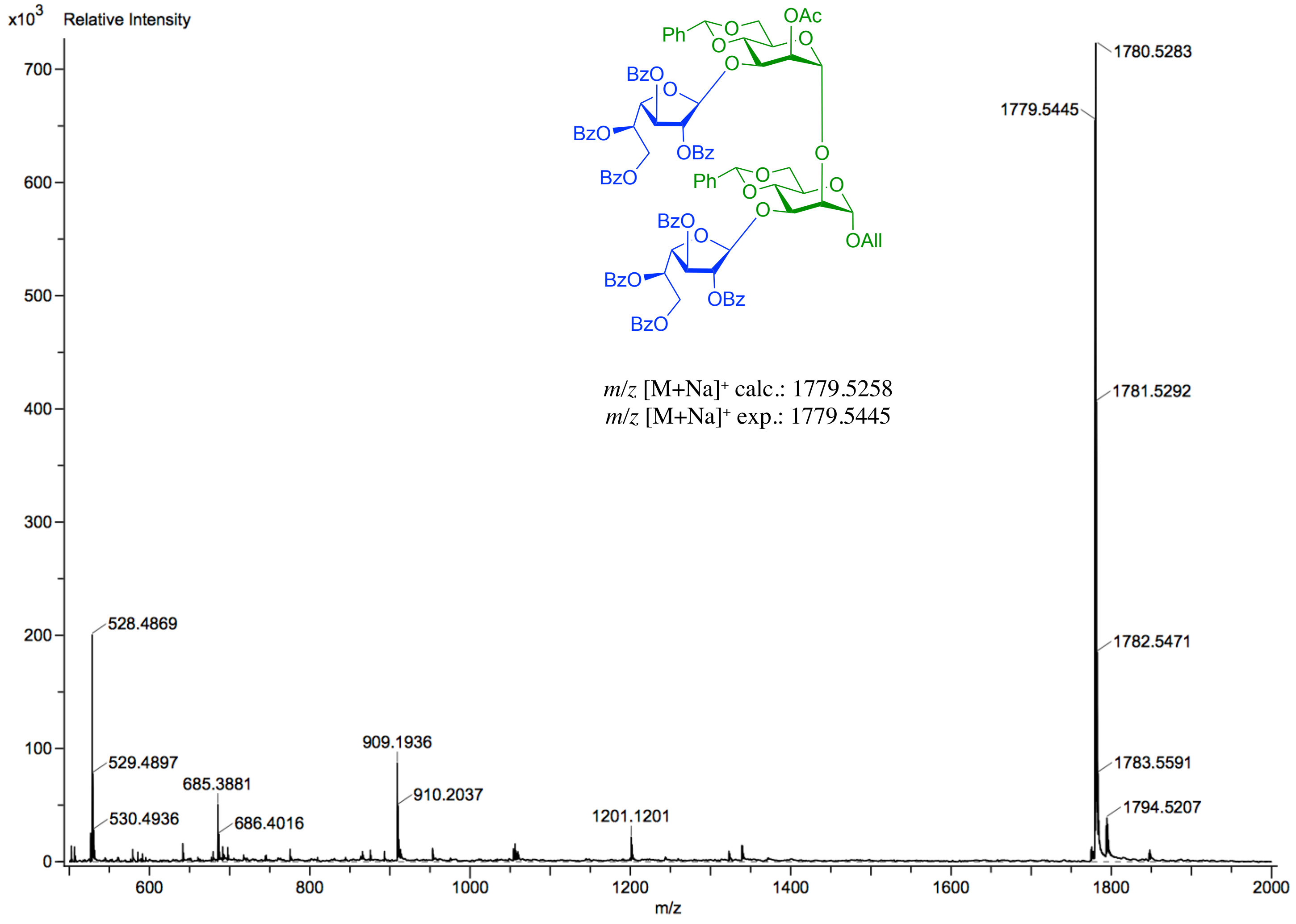

^{1}H NMR spectrum, 400 MHz, $CDCl_3$, compound S4

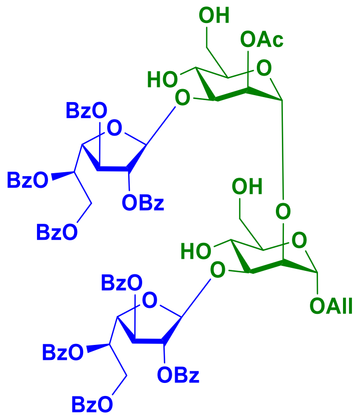

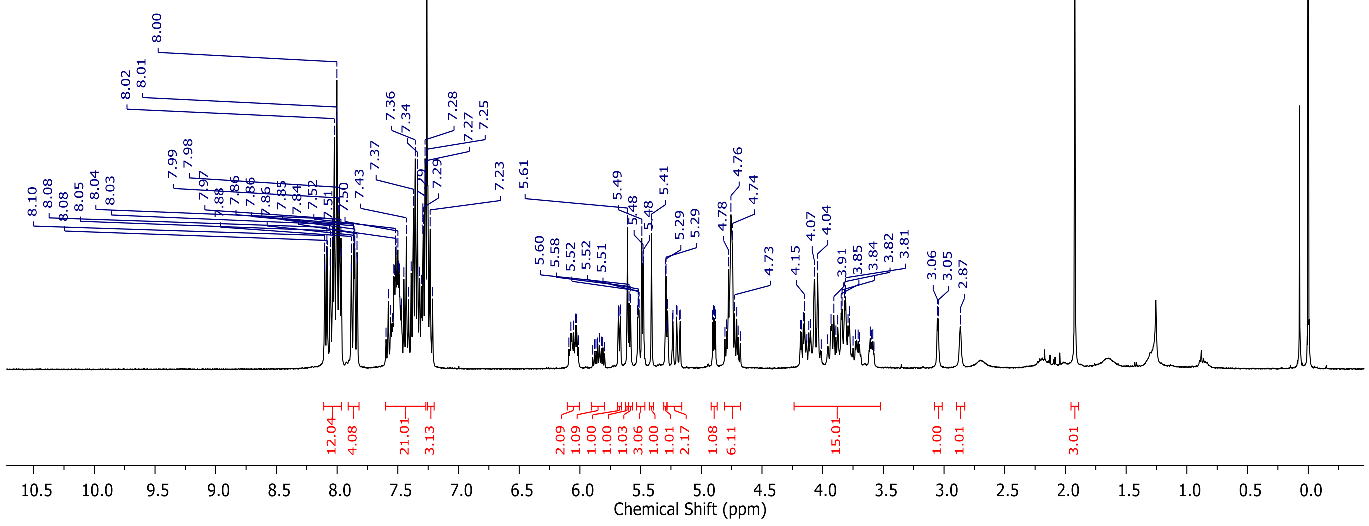

^{13}C NMR spectrum, 100 MHz, $CDCl_3$, compound S4

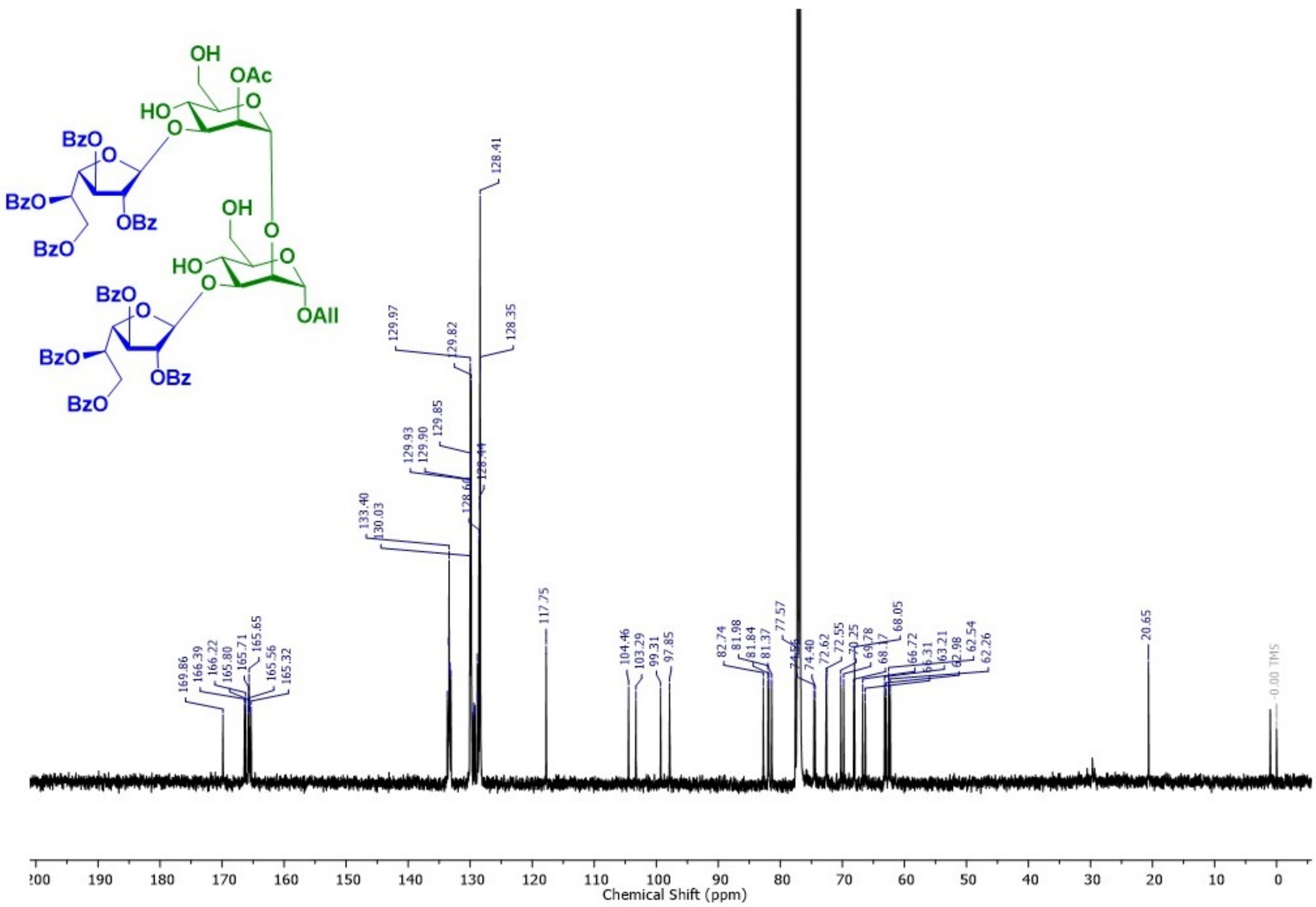

ESI-TOF HR mass spectrum of compound S4

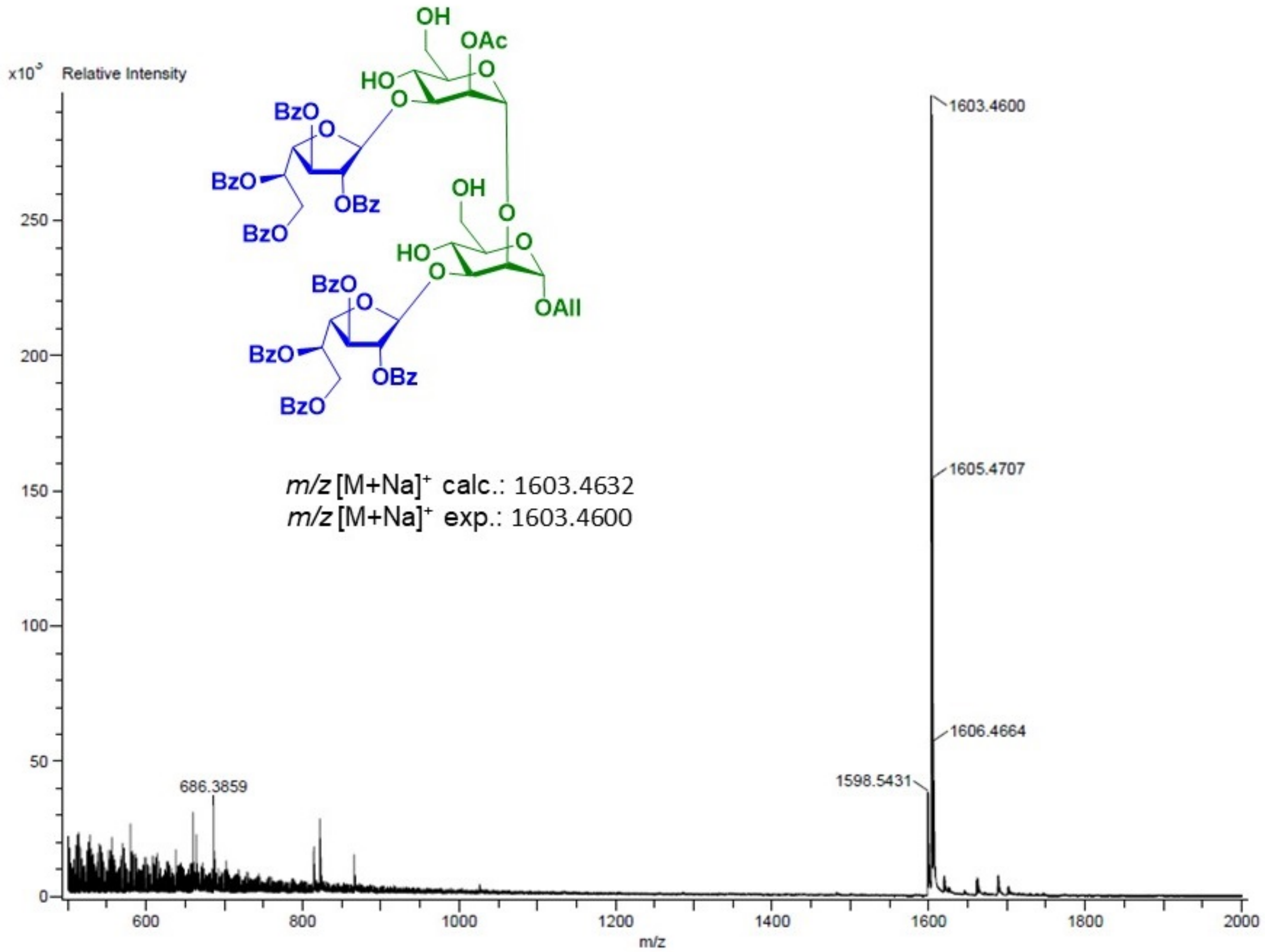

^{1}H NMR spectrum, 400 MHz, $CDCl_3$, compound S5

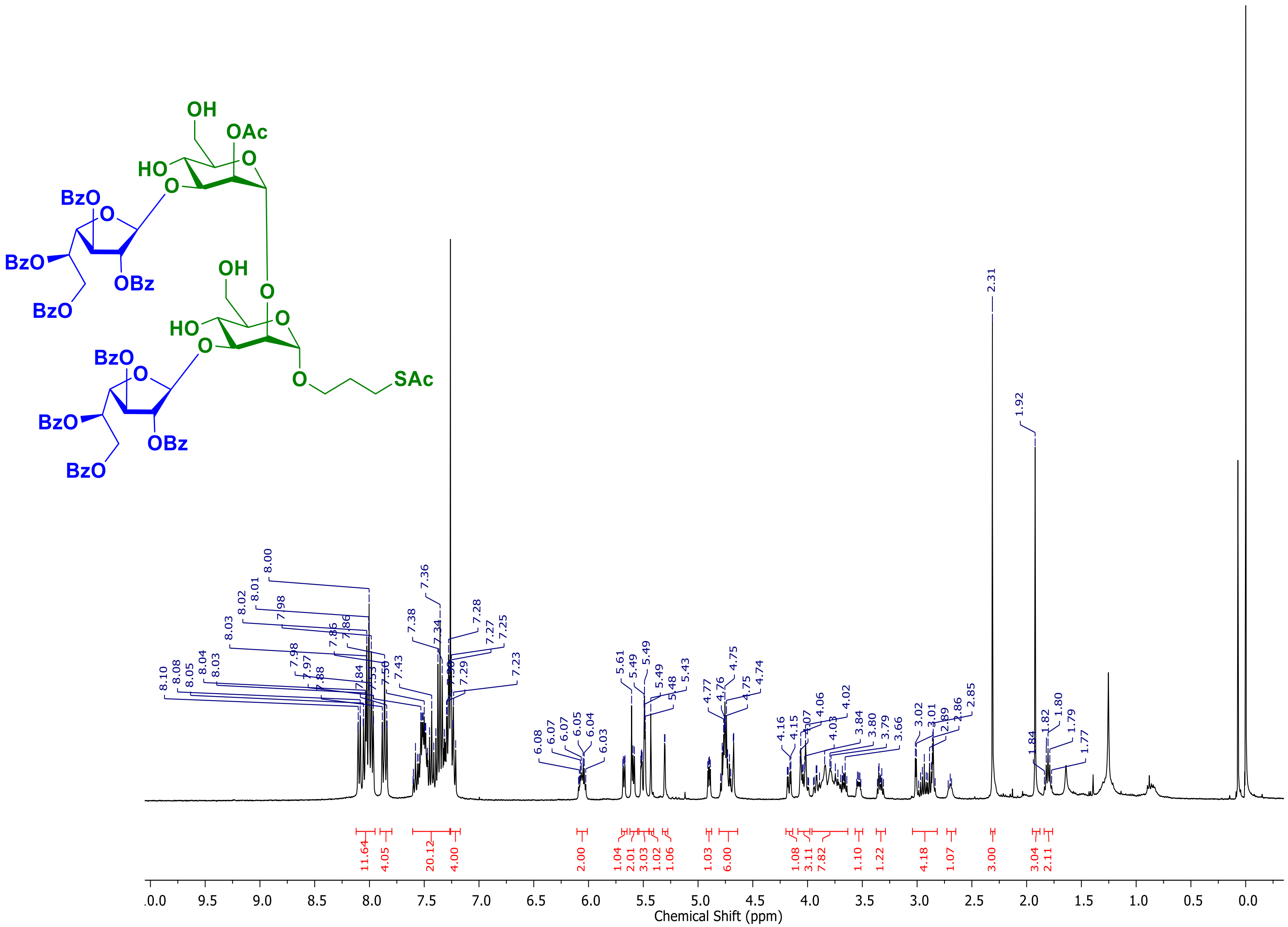

^{13}C NMR spectrum, 100 MHz, $CDCl_3$, compound S5

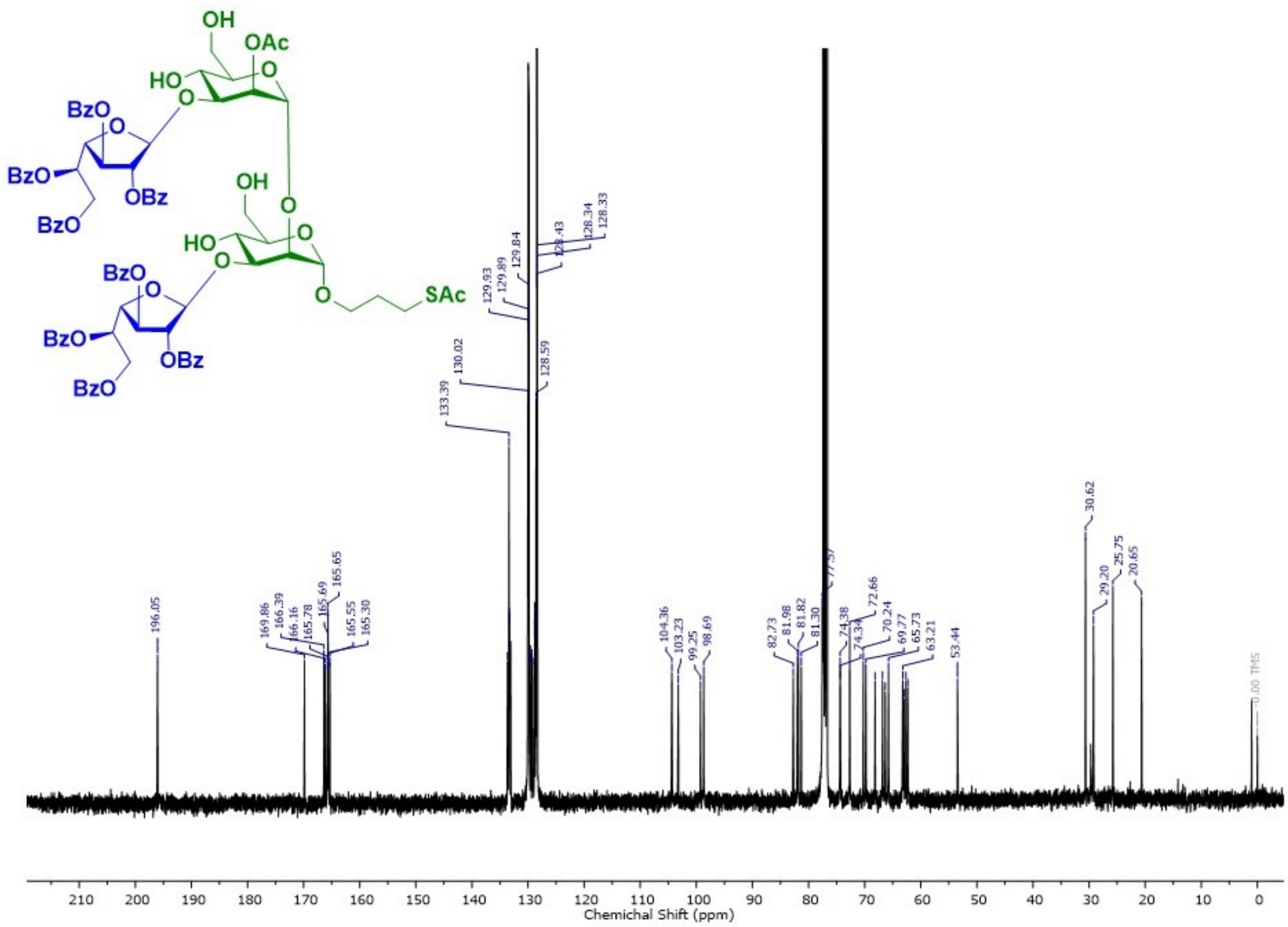

ESI-TOF HR mass spectrum of compound S5

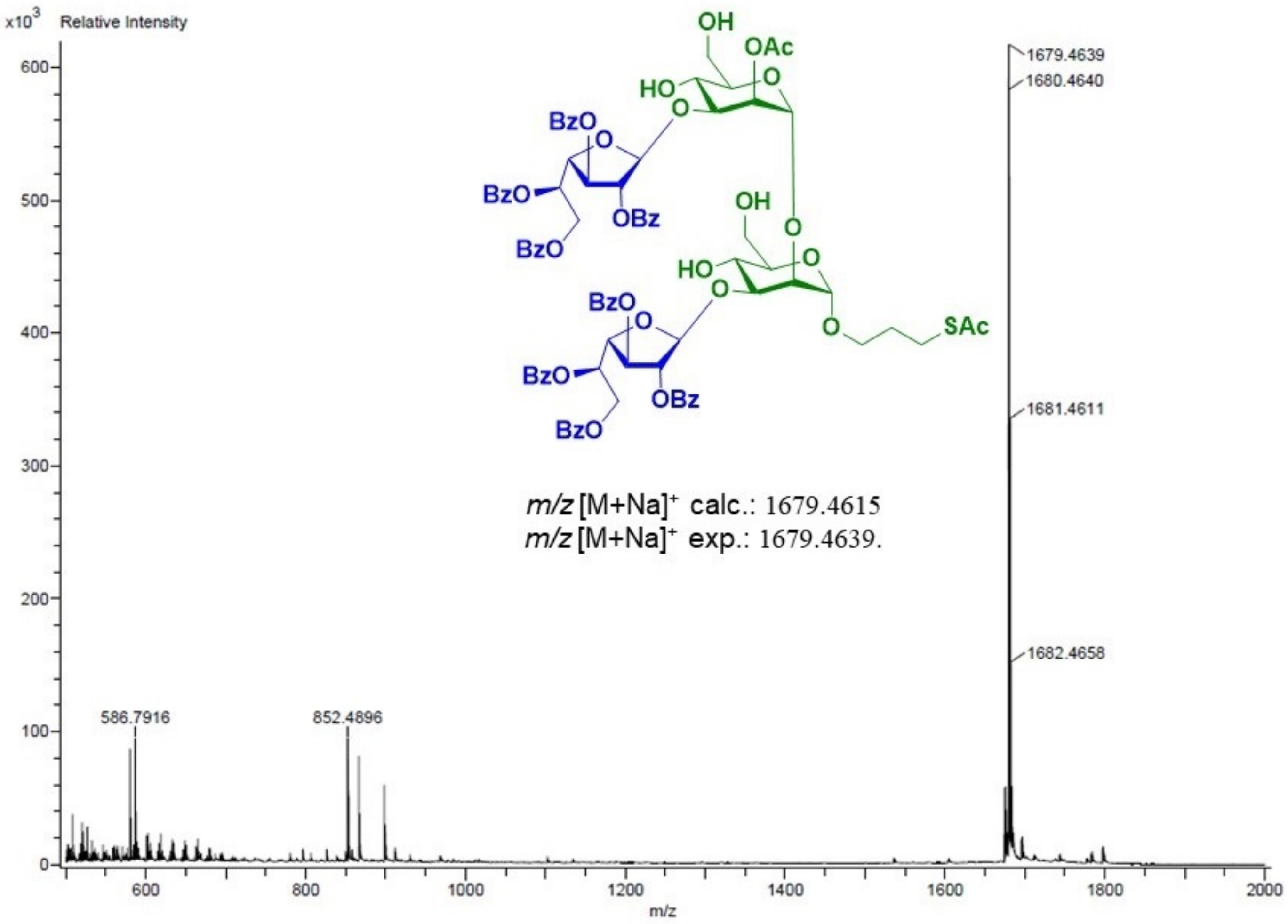

¹H NMR spectrum, 400 MHz, D_2O, compound G32$_{SH}$ [and $(G32_S)_2$]

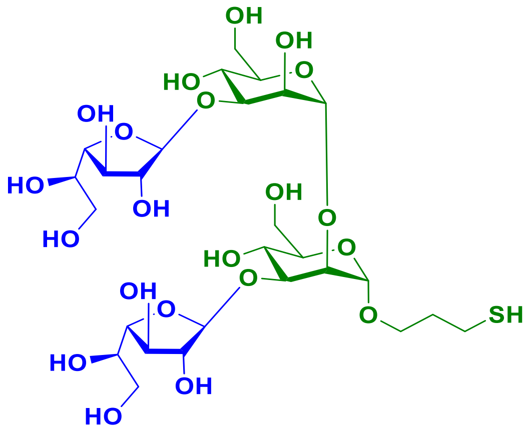

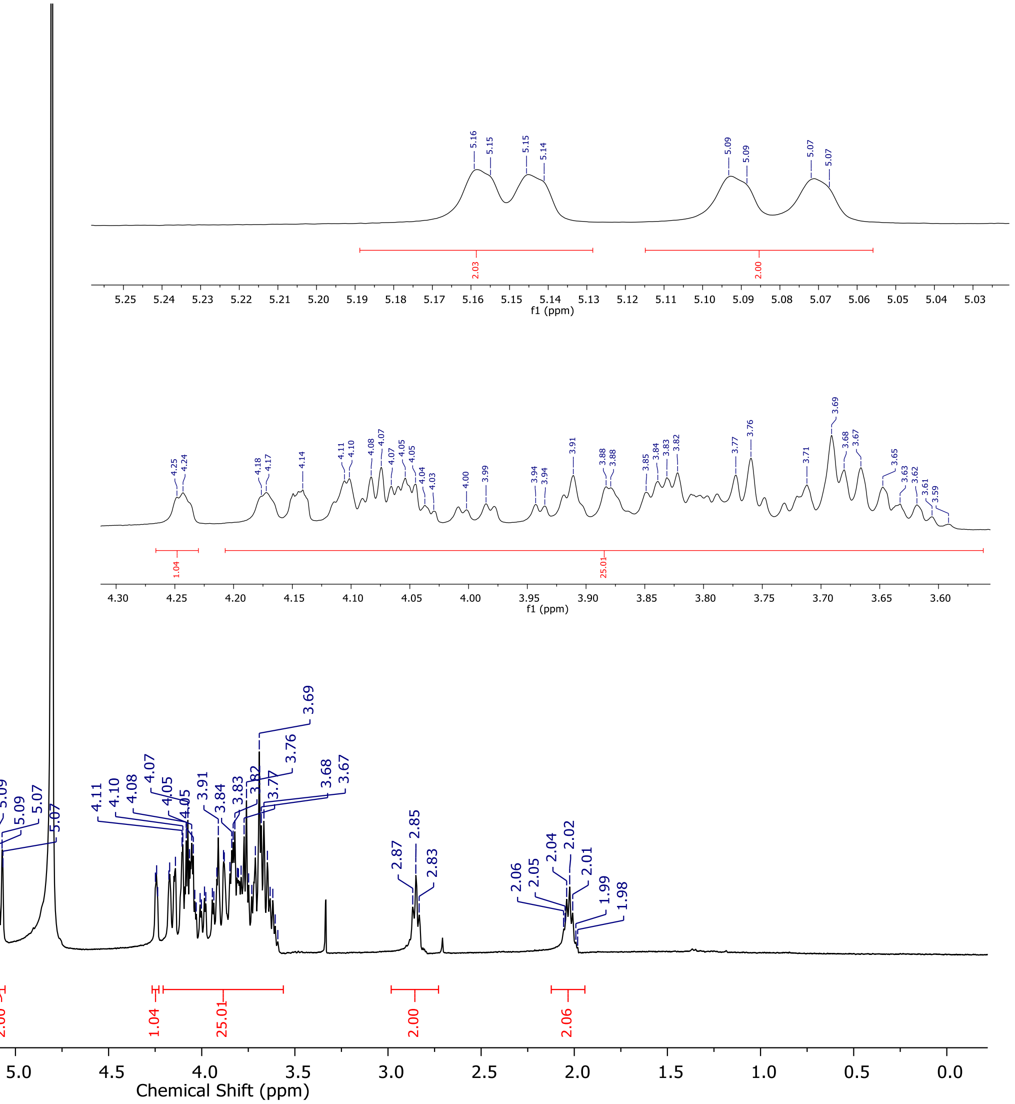

^{13}C NMR spectrum, 100 MHz, D_2O, compound G32$_{SH}$ [and (G32$_{S}$)$_2$]

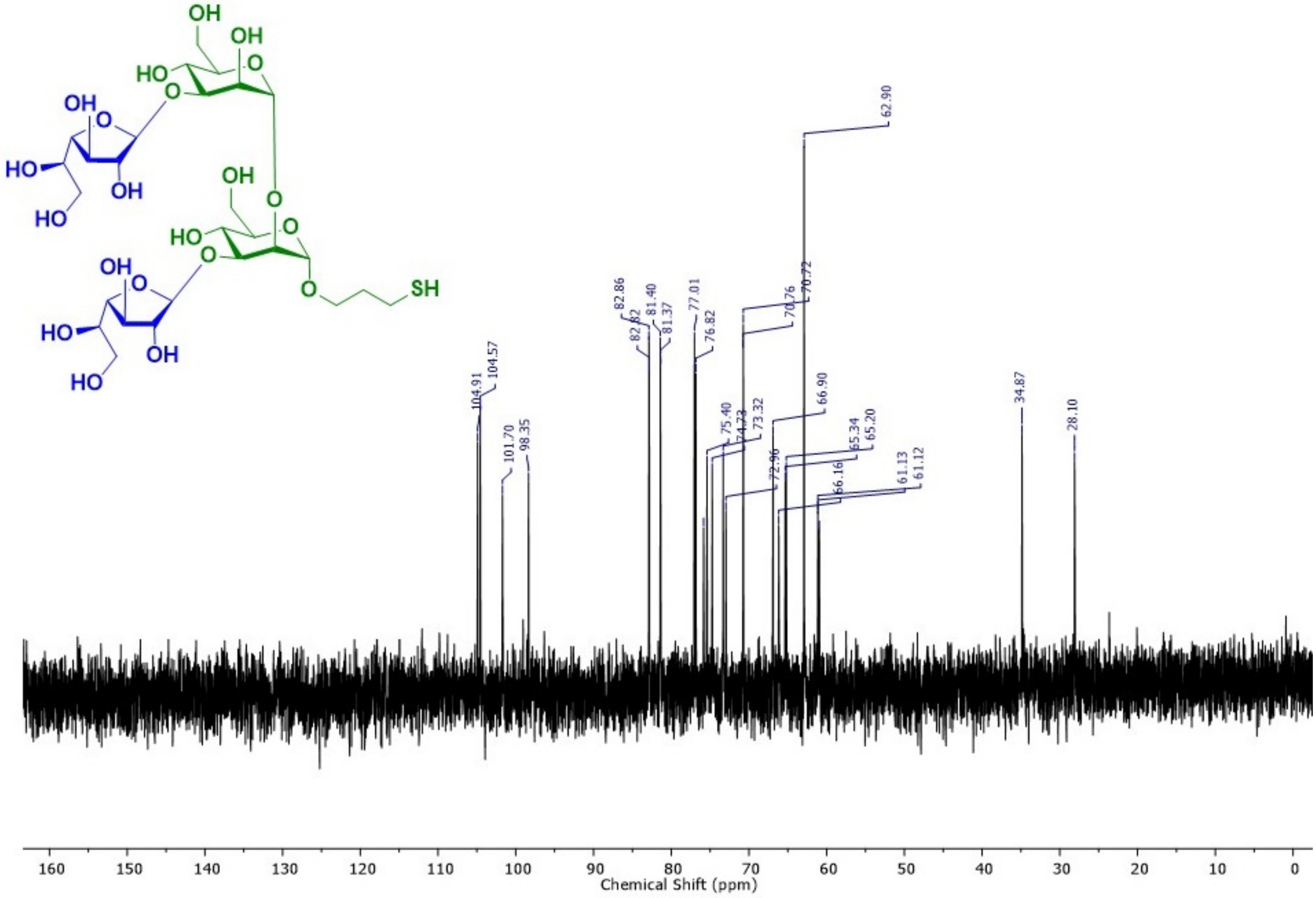

ESI-TOF HR mass spectrum of compounds $G32_{SH}$ and $(G32_S)_2$

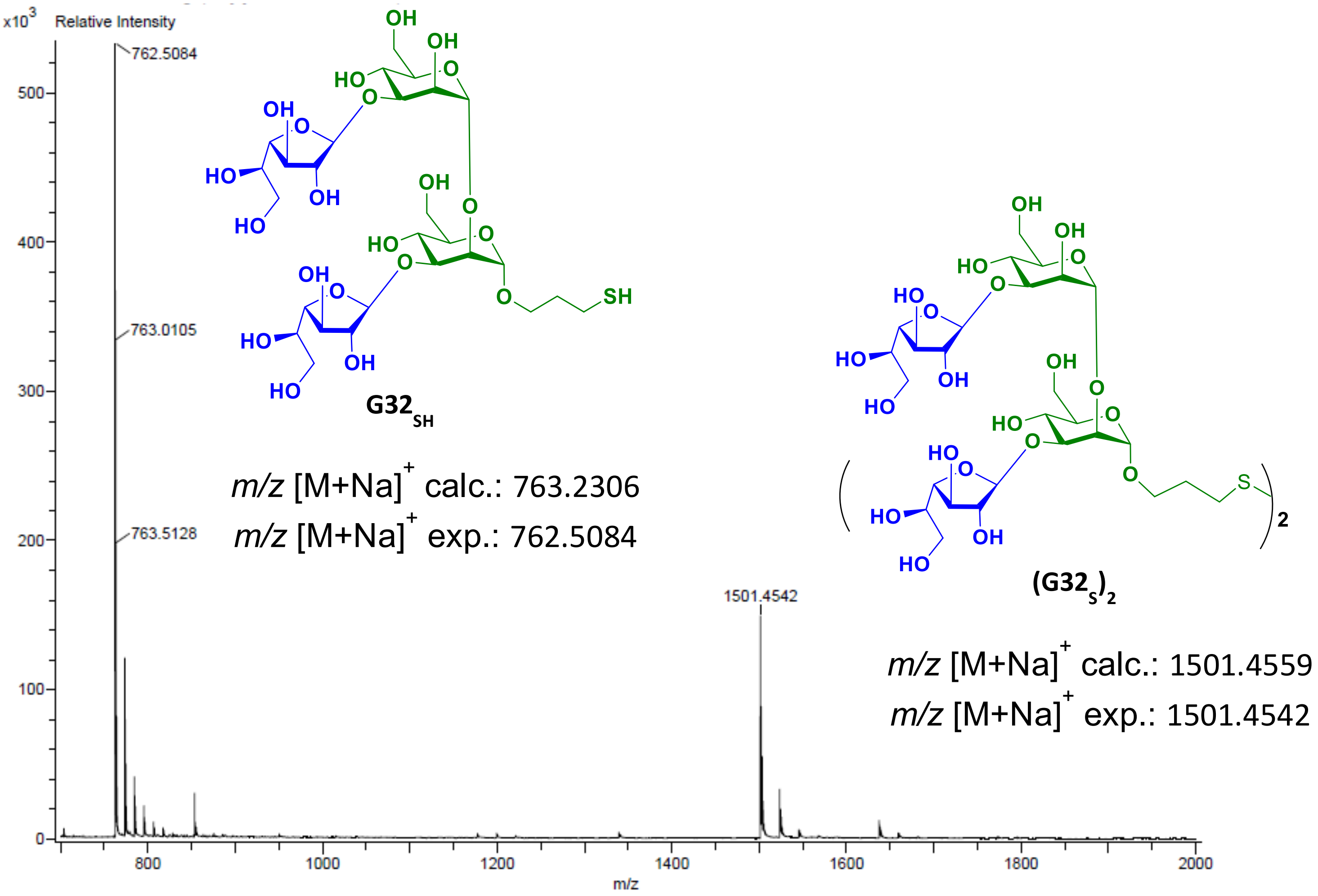

Supplement: Supplementary file 1 [file molecules-27-00411-s001.zip › molecules-1519451-supplementary.pdf]
